# The miR-2110/*TRAF3* axis is associated with endothelial dysfunction and atherosclerosis in coronary heart disease

**DOI:** 10.1016/j.bbrep.2026.102508

**Published:** 2026-02-20

**Authors:** ThanhLoan Tran, Zhong-Yu Wang, Pei-Shan Li, Ying Yang, Yi-Wei Zhang, Shu-Ming Zhang, PhongSon Dinh, NgocLong Le, TrungHieu Pham, Ling Huang, Ning-Yuan Chen, Jun-Hua Peng, Shang-Ling Pan

**Affiliations:** a Department of Pathophysiology, Guangxi Medical University, Nanning, Guangxi, 530021, China; b Department of Immunology and Pathophysiology, Hue University of Medicine and Pharmacy, Hue University, Hue, 520000, Viet Nam; c Department of Internal Medicine, Leshan People's Hospital, Sichuan, 614001, China; d Department of Clinical Pathology, Jingzhou First People's Hospital, Hubei, 434000, China; e College of Medicine and Pharmacy, Duy Tan University, Da Nang, 550000, Viet Nam; f Department of General Medicine, Binh Duong General Hospital, Binh Duong, 820000, Viet Nam; g Department of Urology, Binh Duong General Hospital, Binh Duong, 820000, Viet Nam; h Department of Urology, The First Affiliated Hospital of Guangxi Medical University, Nanning, Guangxi, 530021, China; i Guangxi Colleges and Universities Key Laboratory of Human Development and Disease Research, Guangxi Medical University, Nanning, Guangxi, 530021, China

**Keywords:** miR-2110, Coronary heart disease, Endothelial dysfunction, Atherosclerosis, *TRAF3*, Inflammatory signaling

## Abstract

Coronary heart disease (CHD) is driven by endothelial dysfunction and chronic vascular inflammation. hsa-miR-2110 (miR-2110) has been associated with adverse cardiovascular outcomes, but its mechanistic role in CHD remains unclear. In this study, miR-2110 expression was quantified in peripheral blood from CHD patients and healthy controls. Functional effects were assessed in EA.hy926 endothelial cells following lentiviral overexpression of miR-2110. The target gene *TRAF3* was identified by RNA sequencing, bioinformatic analysis, and validated by dual-luciferase reporter assays, RT-qPCR, and Western blotting. *TRAF3* expression was further evaluated in CHD patient blood samples and in atherosclerotic lesions from ApoE^−/−^ mice fed a high-fat diet. miR-2110 was significantly downregulated in CHD patients. Overexpression of miR-2110 in endothelial cells impaired proliferation and migration, induced S-phase arrest, reduced apoptosis, and promoted cellular senescence. *TRAF3* was confirmed as a direct target of miR-2110. *TRAF3* was significantly upregulated in CHD patients. In ApoE^−/−^ mice, TRAF3 protein expression was increased in atherosclerotic lesions, predominantly within the tunica intima. Pathway enrichment predicted NF-κB–related signaling among the enriched pathways potentially associated with the miR-2110/*TRAF3* axis. Together, our findings suggest that the miR-2110/*TRAF3* axis represents a novel regulatory pathway involved in CHD, potentially relevant to endothelial dysfunction and inflammatory signaling.

## Highlights

• miR-2110 is downregulated in coronary heart disease and is associated with endothelial dysfunction.
• TRAF3 is identified as a direct target of miR-2110 in endothelial cells.
• Dysregulated miR-2110/TRAF3 expression is observed in coronary heart disease.

## Graphical abstract

**Figure:**
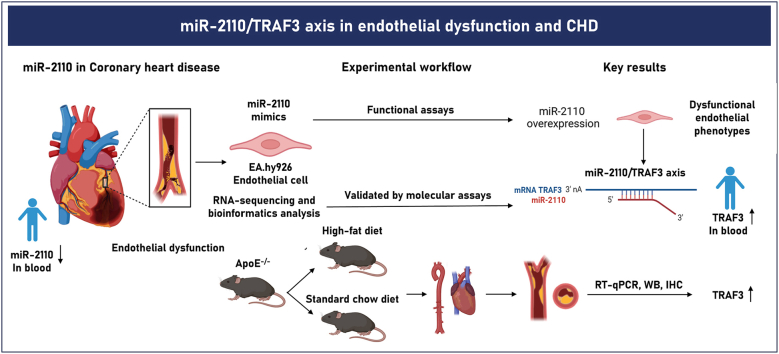

## 1 Introduction

Coronary heart disease (CHD) is primarily driven by atherosclerosis, a chronic progressive process in which lipid accumulation, endothelial injury, and persistent vascular inflammation culminate in the narrowing or occlusion of coronary arteries, leading to myocardial ischemia and hypoxia [1]. When exposed to cardiovascular risk factors, endothelial cells (ECs) lose their protective functions and develop a pro-inflammatory phenotype, promoting leukocyte adhesion, vascular inflammation, and plaque formation. This endothelial dysfunction is both a hallmark and a key driver of CHD progression [2]. Despite advances in prevention, diagnosis, and treatment, CHD remains a leading cause of morbidity and mortality worldwide [3]. These observations underscore the complexity of CHD pathogenesis, which involves not only lipid metabolism and vascular inflammation but also genetic and epigenetic regulation.

MicroRNAs (miRNAs) are small non-coding RNAs, typically around 20 nucleotides in length, that regulate gene expression at the post-transcriptional level through binding to the 3′ untranslated region (3′UTR) of target mRNAs [4]. Accumulating evidence indicates that miRNAs play critical roles in cardiovascular diseases by modulating ECs function, vascular smooth muscle cell phenotype, and inflammatory responses within the vessel wall [[5], [6], [7], [8]]. Altered miRNA expression can contribute to endothelial dysfunction, lipid deposition, and atherogenesis, making miRNAs promising candidates for CHD biomarkers and therapeutic targets [9].

Recent studies have revealed that hsa-miR-2110 (miR-2110) expression is significantly downregulated in patients with acute myocardial infarction (MI) and heart failure compared with those with prior MI [10]. Analysis of whole blood samples from CHD patients has shown further downregulation of this miRNA in individuals experiencing recurrent cardiovascular events [11]. Moreover, miR-2110 was among the five miRNAs most significantly downregulated in patients with post-MI heart failure compared with those with preserved cardiac function [12]. These clinical conditions represent advanced manifestations of coronary heart disease and are characterized by chronic vascular inflammation and endothelial injury. Although direct evidence linking miR-2110 to atherosclerosis or endothelial dysfunction is currently limited, these observations suggest that miR-2110 may be associated with vascular pathophysiology and provide a rationale for exploring its potential role in endothelial dysfunction and CHD. However, the involvement of miR-2110 in CHD has not yet been investigated.

In this study, we first evaluated miR-2110 expression in peripheral blood samples from CHD patients and controls. We then investigated its functional effects and its potential downstream target genes *in vitro* using ECs models. Finally, *in vivo* validation was performed to confirm the relevance of our findings. Together, our work aims to elucidate the potential roles of miR-2110 and its regulatory mechanism in CHD pathogenesis.

## 2 Materials and methods

### 2.1 Study subjects

This study included 44 patients with CHD (age range: 41-84; mean age: 63.16 ± 12.70 years) and 43 age-matched healthy controls (age range: 45-82, mean age: 60.79 ± 8.65 years), recruited from the Department of Cardiology and the Medical Check-up Center of the People's Hospital of Guangxi Zhuang Autonomous Region, respectively. CHD diagnosis was based on angiographically confirmed ≥50% stenosis in at least one major coronary artery, electrocardiographic evidence of myocardial ischemia, color Doppler echocardiography to evaluate cardiac structure and function, and other relevant clinical assessments. Exclusion criteria included diabetes mellitus, major organ dysfunction, malignancies, autoimmune or hematologic disorders, and psychiatric diseases. Healthy controls had no history or evidence of cardiovascular disease and no age-related comorbidities detected during routine health screening. Detailed inclusion and exclusion criteria were previously published [13,14]. All participants provided written informed consent. The study protocol was approved by the Ethics Committee of Guangxi Medical University (Approval no. 2019-SB-060) and conducted in accordance with the 1975 Declaration of Helsinki.

### 2.2 Data and sample collection

Demographic and clinical data, including age, sex, body mass index (BMI), blood pressure, and complete blood count, were recorded. For each participant, 4 mL of venous blood was collected after overnight fasting: 2 mL in EDTA tubes for RNA extraction, and 2 mL in plain tubes (without anticoagulant) for serum biochemical analysis, including total cholesterol (TC), triglycerides (TG), low-density lipoprotein cholesterol (LDL-C), and high-density lipoprotein cholesterol (HDL-C), measured using a Hitachi Automated Biochemical Analyzer LABOSPECT 008 AS (Japan).

### 2.3 RNA extraction and real-time quantitative polymerase chain reaction (RT-qPCR)

miRNAs were extracted from peripheral blood and EA.hy926 cells using the SanPrep Column microRNA Extraction Kit (Sangon Biotech, China, B518811-0050). mRNAs were extracted from EA.hy926 cells, peripheral blood and mice tissues using AxyPrep Total RNA Miniprep Kit (Axygen, China, AP-MN-MS-RNA-250). miRNA First Strand cDNA Synthesis (Tailing Reaction) Kit (Sangon Biotech, China, B532451) was used to reversely transcribe miRNA to cDNA, while mRNA was reversely transcribed to cDNA by HiScript® III RT SuperMix for qPCR Kit (+gDNA wiper) (Vazyme, China, R323-01), following manufacturer's instructions. The relative expression of miRNA and mRNAs were detected by RT-qPCR using U6snRNA and GAPDH as normalization controls, respectively. All RT-qPCR reactions were run in duplicate on a StepOne™ Real-Time PCR System (Applied Biosystems, USA) using 2 × Universal SYBR Green Fast qPCR Mix (ABclonal, China, RK21203) in 20 μL reaction volumes, following manufacturer's protocol. RT-qPCR was performed with distinct thermal profiles: for miRNA - 95 °C for 30 s, then 40 cycles of 95 °C (5 s)/60 °C (34 s); for mRNA - 95 °C for 30 s, then 40 cycles of 95 °C (10 s)/60 °C (30 s). Melting curve analysis (60 °C to 95 °C at 0.3 °C/s) confirmed amplification specificity. Data were analyzed using StepOne™ Software v2.3, with relative quantification *via* 2^−ΔΔCt^ method [15]. Primers (synthesized by Nanning Janis Biotechnology Company, China) were designed using NCBI Primer-BLAST or referenced from published studies; their sequences and sources are provided in Table 1. All in-house-designed primers were validated for specificity and amplification efficiency prior to use.

**Table 1:** Primers for RT-qPCR.

Gene/Target	Species	Primer type	Primer Sequence (5’→3′)	Source/Note
miR-2110	Human	Forward	GGAAACGGCCGCTGAGTGAAAA	Designed in-house
		Reverse	Universal reverse primer	Sangon Biotech, China, B518811-0050
U6	Human	Forward	CAGCACATATACTAAAATTGGAACG	[16]
		Reverse	ACGAATTTGCGTGTCATCC	
TRAF3	Human	Forward	CCCTGTCCCTTTACAGCCAG	Designed in-house
		Reverse	AAAACAGCGACAAGTGCGTC	
GAPDH	Human	Forward	GACAGTCAGCCGCATCTTCT	[17]
		Reverse	CGCCCAATACGACCAAATC	
Traf3	*Mus musculus*	Forward	TCTCCCTCCCTTCTGAGCTT	Designed in-house
		Reverse	GCTTTAGGGGTGGGTTAGGC	
Gapdh	*Mus musculus*	Forward	AGGTCGGTGTGAACGGATTTG	[18]
		Reverse	TGTAGACCATGTAGTTGAGGTCA	

### 2.4 Cell culture and treatment

The EA.hy926 endothelial cell line (experimental model) and 293T cell line (viral packaging) were purchased from Shanghai Cell Bank (Chinese Academy of Sciences). Cells were maintained in complete Dulbecco's modified eagle's medium (DMEM) (Gibco, USA, C11995500BT), supplemented with 10% fetal bovine serum (FBS) (Biological Industries, Israel, 04-001-1ACS) and 1% penicillin/streptomycin solution (Solarbio, China, P1400) under standard conditions (37 °C, 5% CO_2_). Cells were routinely passaged at 80% confluence using trypsin-EDTA solution (Sangon Biotech, China, E607002-0100).

For miR-2110 functional studies, lentiviral particles were generated by co-transfecting 293T cells with miR-2110 mimic plasmids (GeneCopoeia, USA, #HmiR0720-MR03) or mimic negative control (mimic NC) (GeneCopoeia, USA, #CmiR0001-MR03) using the Lenti-Pac™ HIV Packaging Kit (GeneCopoeia, USA, LT002) according to the manufacturer's protocol. EA.hy926 cells at 50-70% confluence were transduced with viral supernatants. After 96 h, stable transfectants were selected with 2 μg/mL puromycin (Solarbio, China, P8230) for 72 h. Transfection efficiency was assessed *via* GFP fluorescence imaging (excitation: 488 nm/emission: 520 nm) using a Leica DMi8 inverted microscope equipped with LAS X software (Leica Microsystems, Germany). miR-2110 expression levels were further validated by RT-qPCR.

### 2.5 Assessment of miR-2110 effects on EA.hy926 cell functions

The two groups of miR-2110-mimic and mimic NC EA.hy926 cells were used to investigate the impact of miR-2110 on cellular behaviors.

- *Proliferation assay:* Cells were seeded at 4 × 10^3^ cells/well in 96-well plates. Cell proliferation was assessed using Cell Counting Kit-8 (CCK-8) reagent (Vazyme, China, A311-01-AA) every 24 h to 96 h to obtain the proliferation curve. After adding 10 μL of CCK-8 solution to each well and incubating for 1h at 37 °C and 5% CO_2_, optical density (OD) at 450 nm was measured using a microplate reader (BIO-RAD, USA).
- *Scratch wound healing assay:* Cells were seeded into 6-well plates at 2 × 10^5^ cells/well and incubated for 24 h. Scratches were made in the cell layer using a 1000 μL pipette tip, and after washing with PBS, cells were cultured in DMEM with 2% FBS. Images of the wound area were taken at 0, 12, 24, and 36 h by Leica DMi8 microscope, and relative migration area was analyzed using ImageJ (version 1.5.3, National Institutes of Health, USA).
- *Transwell migration assay:* 500 μL DMEM with 3% FBS was added to each well of a 24-well plate, followed by a Transwell® chamber (8.0 μm pore size, Costar®, USA 3422) gently placed into each well to avoid air bubbles. A volume of 100 μL DMEM with 2% FBS, containing 2 × 10^4^ cells were added to the Transwell chamber. After 24 h, migrated cells were fixed with absolute methanol for 30 min, stained with 500 μL of 0.5% crystal violet (Solarbio, China, G1062) for 30 min and counted in five randomly selected fields under a microscope using ImageJ (version 1.5.3).
- *Flow cytometry:* Cells were harvested and stained with Cell Cycle Staining Kit (Multi Sciences, China, 70-CCS012), then incubated for 30 min at room temperature in the dark. The distribution of cells in each cell cycle phase (G0/G1, S, G2/M) was analyzed using a BD FACSCalibur flow cytometer (BD Biosciences, USA). For cell apoptosis analysis, cells were double-stained with Annexin V-APC and 7-AAD using the Annexin V-APC/7-AAD Apoptosis Kit (Multi Sciences, China, AP105-100-kit) according to the manufacturer's protocol. Apoptotic cells (Annexin V+/7-AAD− for early apoptosis; Annexin V+/7-AAD + for late apoptosis) were quantified by flow cytometry (BD FACSCalibur, USA). Data analysis was performed using FlowJo™ software (Version 10.8, BD Life Sciences, USA).

### 2.6 RNA-sequencing and bioinformatic analysis for target gene prediction

Total RNA was extracted from miR-2110 mimic and mimic NC groups, followed by mRNA purification. cDNA libraries were constructed and subjected to high-throughput RNA sequencing (RNA-seq) (Illumina platform, performed by Shanghai Tianhao Biotechnology Co.). Raw count data were normalized using the DESeq2 median-of-ratios method to account for library size differences. Differentially expressed genes were identified using a negative binomial generalized linear model. P values were adjusted for multiple testing using the Benjamini–Hochberg false discovery rate (FDR) correction, and genes with adjusted P < 0.05 were considered statistically significant. For target prediction, putative miR-2110 target genes were identified by intersecting TargetScan predictions (v8.0, https://www.targetscan.org/vert_80) and Differentially expressed genes (DEGs) from RNA-seq data (|Log2FC| > 0.5, *P* < 0.05). Functional enrichment analysis of target genes was performed using the “enrichR” package in R Studio (version 4.3.1), querying Gene Ontology (GO) (biological process, cellular component, molecular function) (http://geneontology.org) and Kyoto Encyclopedia of Genes and Genomes (KEGG) (https://www.kegg.jp/kegg/pathway.html) databases with significance thresholds set at P < 0.05.

### 2.7 Dual-luciferase assay

For the luciferase reporter assays, wild-type (WT) and mutated (MT) *TRAF3* 3′UTR sequences were cloned into the pmirGLO dual-luciferase vectors (Promega, USA), which contained both Firefly luciferase (Luc) and Renilla luciferase (Rluc) reporter genes. Cells were co-transfected with either *TRAF3*-WT or *TRAF3*-MT plasmids along with miR-2110 mimic or mimic NC vector, using LipofectamineTM 2000 transfection reagent (Invitrogen, USA). At 48 h post-transfection, luciferase activity was measured using the Dual-Luciferase® Reporter Gene Assay System (Promega, USA) on a Lumat LB 9508 microplate luminometer (Berthold Technologies, Germany). Relative luciferase activity was calculated as the ratio of Luc to Rluc, with Rluc as the internal normalization.

### 2.8 Western blotting analysis

Cells or mice tissues were lysed in ice-cold RIPA buffer (Beyotime Biotechnology, China, P0013B), supplemented with protease inhibitors and phosphatase inhibitors (Beyotime Biotechnology, China, P1045), and incubated on ice for 15 min. Tissue samples were mechanically homogenized prior to lysis. After centrifugation at 12,000 rpm for 10 min at 4 °C, proteins in the supernatants were quantified with BCA protein assay kit (Beyotime Biotechnology, China, P0010S), then separated by 12.5% SDS-PAGE (Enzymatic Biotechnology, China) and subsequently transferred to 0.22 μm PVDF membrane (Merck Millipore, Germany, ISEQ00010). After blocking with 5% skimmilk in PBS, the membranes were immunoblotted overnight at 4 °C with antibodies against TRAF3 (1:2000, Proteintech, USA, 18099-1-AP) and GAPDH (1:50,000, Proteintech, USA, 10494-1-AP), followed by HRP-conjugated goat Anti-rabbit IgG (H + L) (1:10000, Servicebio, China, GB23303). The target proteins were detected with ECL reagent (Beyotime Biotechnology, China, P0018S) and captured using the SageCapture Mini Chemiluminescence Imaging System (Sage Creation Science, China, SC-1002) with automatic exposure optimization. Band intensities were quantified using ImageJ v1.53 and normalized to GAPDH levels.

### 2.9 Atherosclerosis mouse model and tissue collection

Male ApoE^−/−^ mice (C57BL/6JNifdc strain, 8 weeks old, 20 ± 2 g) were obtained from Charles River Laboratory (Beijing, China) and maintained under specific pathogen-free conditions (temperature 22–24 °C; humidity 50–60%; 12 h light/dark cycle) in ventilated cages. After a 1-week acclimatization period, body weight and general health status of all mice were assessed to ensure comparable baseline characteristics between groups. Mice were then randomly assigned to two groups (n = 6 per group): Experimental group, fed a high-fat diet (HFD; 20% protein, 40% carbohydrates, 40% fat; Beijing Huafukang Biotechnology Co., China, H10540) to induce atherosclerosis; and Control group, fed standard chow (20% protein, 70% carbohydrate, 10% fat; Beijing Huafukang Biotechnology Co., China, D12450J) *ad libitum*. Diets were maintained for 12 weeks, and body weight was recorded weekly (Supplementary Table 1). At the study endpoint, mice were anesthetized *via* intraperitoneal injection of pentobarbital sodium (150 mg/kg; Shanghai Yuanye BioTech, China, S24063, CAS 57-33-0). Blood was collected by retro-orbital puncture after confirming absence of the pedal reflex, and death was ensured by cervical dislocation.

The entire aorta, from the aortic root to the iliac bifurcation, was carefully dissected. Hearts were excised separately for subsequent analyses. Tissues were either snap-frozen in liquid nitrogen for RNA/protein extraction or fixed in 4% paraformaldehyde (Solarbio, China, P1110) for histological analyses.

All experimental procedures were approved by the Animal Care and Welfare Committee of Guangxi Medical University (Approval No. 202410097) and complied with the Guideline for Ethical Review of Animal Welfare (China National Standard GB/T 35892-2018).

### 2.10 Atherosclerosis lesion observation with Oil Red O and H&E staining

For *en face* plaque visualization, the entire aorta, with selected portions of major branch vessels preserved to show lesion distribution, was stained *en bloc* with Oil Red O (Beyotime, China, C0157S), then opened longitudinally and pinned flat under a Nikon SMZ745T stereo microscope (Nikon, Japan) to visualize lipid-rich atherosclerotic plaques. The heart tissues were dissected and fixed in 4% paraformaldehyde solution for 96 h at room temperature, dehydrated with graded alcohol, followed by xylene (Sinopharm, China, 10023418) clearing and embedding in paraffin (Leica, Germany, 39601095) with a Leica Histocore Arcadia embedding station (Leica, Germany). Serial 5-μm sections were cut using a Leica RM2235 rotary microtome (Leica, Germany), and stained with hematoxylin (Solarbio, China, H8070) and eosin (Solarbio, China, G1120) for general morphological assessment.

### 2.11 Immunohistochemistry

For immunohistochemical analysis, sections underwent standard deparaffinization in xylene and rehydration through graded ethanol, followed by heat-induced epitope retrieval in Tris-EDTA buffer (Servicebio, China, G0002-500 ML) (pH 9.0, 95 °C, 20 min). Endogenous peroxidase activity was quenched with 3% H_2_O_2_ for 10 min at room temperature, followed by blocking with 5% normal goat serum (Solarbio, China, SL038) in PBS for 1 h. Sections were incubated overnight at 4 °C with rabbit anti-TRAF3 monoclonal antibody (1:200, HUABIO, China, PSH01-30), then probed with HRP-conjugated goat anti-rabbit IgG (1:500, Servicebio, China, GB23303) for 1 h at room temperature. Signal development was performed using DAB chromogen (Servicebio, China, G1211-200T), with hematoxylin counterstaining. All slides were imaged using an Olympus DP80 brightfield microscope (Olympus, Japan).

### 2.12 Statistical analysis

All data were analyzed using SPSS v24.0 (IBM, USA), visualized by GraphPad Prism 8.0 (GraphPad Software, USA) and RStudio v4.3.1 (R Foundation, Austria). Data distribution was first assessed for normality prior to statistical testing using the Shapiro–Wilk test. Based on data distribution, normally distributed variables are presented as mean ± SD and compared using unpaired two-tailed Student's t-tests. Non-normally distributed variables are presented as median [interquartile range, IQR] and compared using Mann–Whitney U tests. Categorical variables (e.g., sex distribution) were expressed as counts and analyzed using *chi-square* tests. All *in vitro* experiments were repeated independently three times. *In vivo* experiments included n = 6 mice per group. P < 0.05 was considered statistically significant.

## 3 Results

### 3.1 Clinical characteristics of study participants

There were 44 CHD patients (aged 63.16 ± 12.70 years) and 43 age-matched healthy controls (aged 60.79 ± 8.65 years) participated in the study. As shown in Table 2, CHD patients had significantly higher white blood cell (WBC) counts (*P* = 0.0005) and neutrophil (NEU) counts (*P* = 0.0018), and significantly lower HDL-C levels (*P* < 0.0001) compared with healthy controls.

**Table 2:** Clinical parameters of the CHD patients and healthy controls.

Clinical parameters	Healthy Controls (n = 43)	CHD Patients (n = 44)	t/χ^2^	*P* value
Age (years)	60.79 ± 8.65	63.16 ± 12.70	1.015	0.3132
Sex (M/F)	26/17	29/15	0.277	0.5986
SBP (mmHg)	133.00 ± 20.86	135.90 ± 17.70	0.708	0.4811
DBP (mmHg)	78.95 ± 11.69	82.86 ± 13.92	1.417	0.1600
BMI (kg/m2)	23.71 ± 2.96	24.35 ± 3.49	0.914	0.3635
WBC (109/L)	6.17 ± 1.37	7.70 ± 2.35	3.635	0.0005
NEU (109/L)	3.46 ± 0.91	4.52 ± 1.73	3.246	0.0018
RBC (1012/L)	4.80 ± 0.67	4.62 ± 0.53	1.379	0.1717
HGB (g/L)	137.40 ± 14.36	138.9 ± 15.36	0.465	0.6436
HCT (%)	41.35 ± 4.16	42.34 ± 3.76	1.048	0.2983
PLT (109/L)	238.60 ± 57.00	249.90 ± 58.33	0.891	0.3756
FBG (mmol/L)	5.07 ± 0.73	4.90 ± 0.99	0.886	0.3781
TC (mmol/L)	4.83 ± 0.91	4.51 ± 1.40	1.219	0.2262
TG (mmol/L)	1.49 ± 1.02	1.54 ± 0.91	0.248	0.8049
HDL-C (mmol/L)	1.41 ± 0.38	1.10 ± 0.29	4.162	<0.0001
LDL-C (mmol/L)	3.02 ± 0.76	2.73 ± 1.07	1.417	0.1603

**Note:** Continuous variables are expressed as mean ± SD and compared using the independent-samples *t*-test. Categorical variables (gender) were compared using the chi-square test. Abbreviations: SBP, systolic blood pressure; DBP, diastolic blood pressure; BMI, body mass index; WBC, white blood cell; NEU, neutrophile; RBC, red blood cell; HGB, hemoglobin; HCT, hematocrit; PLT, platelet; FBG, fasting blood glucose; TC, total cholesterol; TG, triglyceride; HDL-C, high-density lipoprotein-cholesterol; LDL-C, low-density lipoprotein-cholesterol.

### 3.2 miR-2110 expression is downregulated in patients with coronary heart disease

**Fig. 1:**
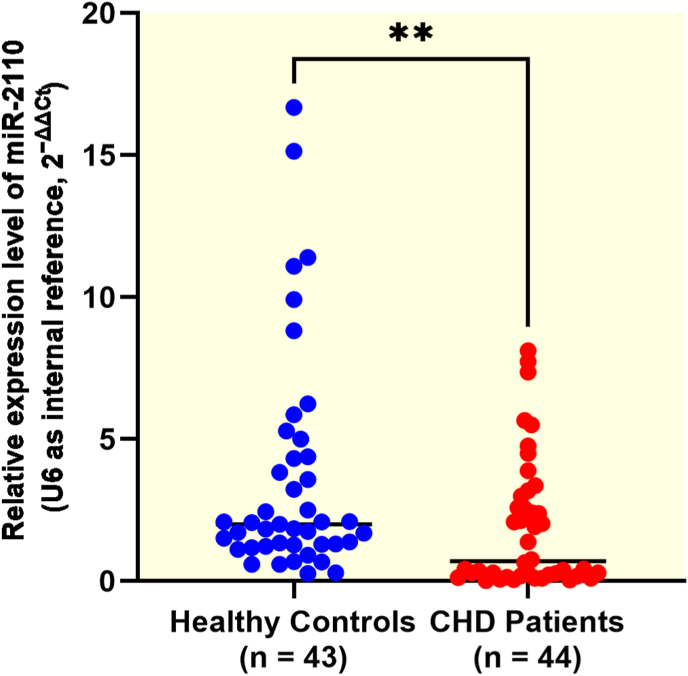
Expression of miR-2110 in patients with CHD and healthy controls Relative expression levels of miR-2110 were quantified by qRT-PCR. Data were presented as median (interquartile range) and were analyzed using the Mann–Whitney *U* test. ∗∗*P* < 0.01.

Quantitative RT-PCR analysis revealed the expression level of miR-2110 was significantly lower in patients with CHD than in age-matched healthy controls (median [IQR]: 0.7020 [0.1962–2.888] vs. 2.000 [1.266–4.377], *P* = 0.0064) (Fig. 1).

### 3.3 miR-2110 overexpressed in EA.hy926 endothelial cell inhibits cell proliferation and migration, arrests cells in S phase, decreases apoptosis rate and increase senescence

**Fig. 2:**
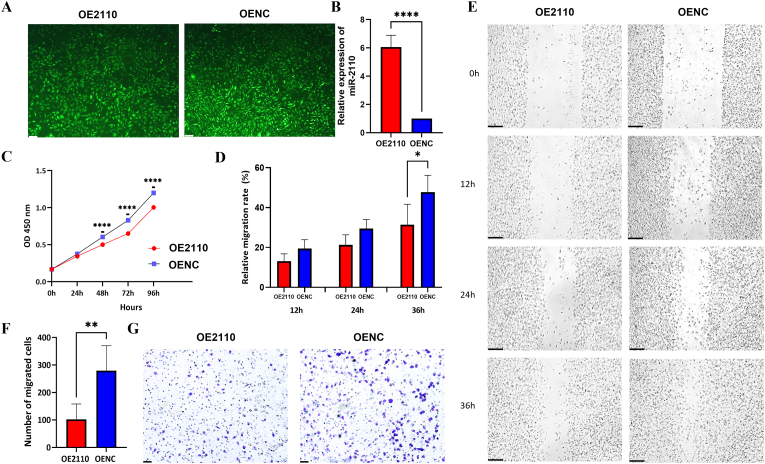
Transfection efficiency of miR-2110 and its effects on proliferation and migration of EA.hy926 cells (**A**) Fluorescence microscopy images showing green fluorescent protein expression in cells infected with lentivirus (100 × , scale bar = 10 μm). (**B**) miR-2110 expression levels in cells transfected with miR-2110 overexpression lentivirus (OE2110) or empty vector control (OENC) were determined by RT-qPCR. (**C**) Cell proliferation rate measured using the CCK-8 assay at OD 450 nm. (**D**) Quantification of relative migration area. (**E**) Representative images of scratch wound healing at 0h, 12h, 24h, and 36h (40 × , scale bar = 50 μm). (**F**) Quantification of migrated cells to the underside of the membrane, crystal violet staining was used for cell visualization. (**G**) Representative images from the Transwell migration assay (100 × , scale bar = 10 μm). ∗*P* < 0.05, ∗∗*P* < 0.01, ∗∗∗∗*P* < 0.0001. Data were presented as mean ± SD from three independent biological experiments (N = 3) using EA.hy926 cells. Statistical comparisons were performed using unpaired two-tailed Student's t-tests.

Given the observed association between reduced circulating miR-2110 levels and CHD, we next examined the functional role of miR-2110 in endothelial cells using an *in vitro* model, given the established contribution of endothelial dysfunction to CHD pathogenesis. We transfected the human vascular endothelial cell line EA.hy926 - a widely used *in vitro* model for vascular studies in CHD - with miR-2110 mimics and mimics negative control (NC). The expression of miR-2110 was first assessed in EA.hy926 cells to confirm baseline expression. RT-qPCR analysis showed that miR-2110 was endogenously detectable in these cells when normalized to U6 (Supplementary Fig. S1). Successful transfection was confirmed by fluorescence microscopy (Fig. 2A) and RT-qPCR (Fig. 2B), which showed an approximately 6-fold increase in miR-2110 expression in miR-2110-overexpressed cells (OE2110) compared to the negative controls (OENC) (*P* < 0.0001). The high transfection efficiency ensured the reliability of subsequent assays.

**Fig. 3:**
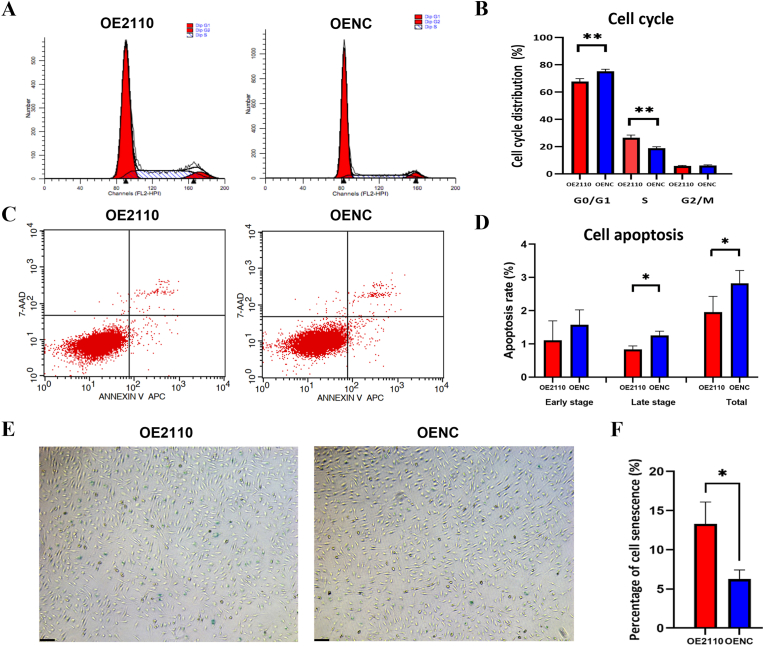
Effects of miR-2110 overexpression on cell cycle distribution, apoptosis, and senescence in EA.hy926 cells (**A**) Quantitative comparison of cell cycle phase distribution between OE2110 and OENC. (**B**) Representative flow cytometry histograms: G0/G1 phase (first red peak), S phase (second broader peak with blue diagonal shading), and G2/M phase (rightmost red peak). (**C**) Representative apoptosis scatter plots: x-axis (Annexin V–APC), y-axis (7-AAD). Quadrants: Q1 (upper left), necrotic/damaged cells; Q2 (upper right), late apoptotic cells; Q3 (lower left), viable cells; Q4 (lower right), early apoptotic cells. (**D**) Quantitative analysis of apoptosis at different stages between OE2110 and OENC. (**E**) Representative images of β-galactosidase–positive (blue) senescent cells under light microscopy (100 × , scale bar = 10 μm). (**F**) Quantitative analysis of senescent cells by β-galactosidase staining. ∗*P* < 0.05, ∗∗*P* < 0.01. Data were presented as mean ± SD from three independent biological experiments (N = 3) using EA.hy926 cells. Statistical comparisons were performed using unpaired two-tailed Student's t-tests.

Functional assays revealed that overexpression of miR-2110 significantly inhibited EA.hy926 cell proliferation compared to the NC group (*P* < 0.0001) (Fig. 2C). Cell migration was assessed using scratch wound healing (Fig. 2D and E) and Transwell migration assay (Fig. 2F and G), both of which showed that overexpression of miR-2110 markedly reduced the migration ability of EA.hy926 cells compared to negative controls (*P* = 0.036). Cell cycle analysis indicated that miR-2110 induced cell cycle arrest at the S phase, as evidenced by a decrease in the proportion of cells in the G0/G1 phase and an increase in the S-phase population (*P* = 0.0086) (Fig. 3A and B). Additionally, miR-2110 overexpression significantly reduced the percentage of cell undergoing late apoptosis and total apoptosis compared to the NC group (*P* = 0.0412) (Fig. 3C and D). β-galactosidase staining revealed a significantly higher proportion of senescent cells in the miR-2110 mimic group compared to the negative control (*P* = 0.0209) (Fig. 3E and F). Collectively, these results demonstrate that miR-2110 suppresses endothelial proliferation and migration, induces S-phase cell cycle arrest, reduces apoptosis, and promotes cellular senescence in EA.hy926 cell. These regulatory effects on endothelial cell behavior, together with the observed downregulation of miR-2110 in CHD patients, suggest a potential role of miR-2110 in the pathogenesis of coronary heart disease.

### 3.4 miR-2110 directly regulates the expression of *TRAF3* in the NF-κB signaling pathway

#### 3.4.1 RNA-sequencing and bioinformatic analysis to identify potential miR-2110's target genes

**Fig. 4:**
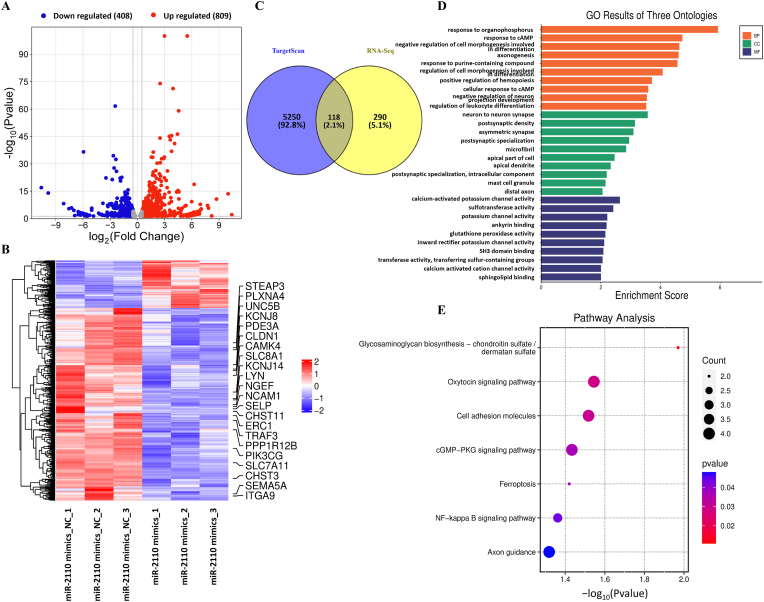
Transcriptomic profiling and functional enrichment analysis of miR-2110-regulated genes in EA.hy926 cells (**A**) Volcano plot of DEGs between OE2110 and OENC groups. Red, upregulated DEGs; blue, downregulated DEGs; grey, non-DEGs. Thresholds: |Log_2_FC| > 0.5, *P* < 0.05. (**B**) Heatmap of all DEGs across three biological replicates per group. Red, upregulation; blue, downregulation; Highlighted gene IDs indicate representative genes involved in significantly enriched GO and KEGG pathways identified in downstream functional analyses. (**C**) Venn diagram showing the overlap between DEGs and predicted miR-2110 target genes from TargetScan, identifying 118 common genes. (**D**) GO enrichment analysis of the 118 overlapping genes, grouped into biological process (BP), cellular component (CC), and molecular function (MF). Enrichment score reflects the degree of association. (**E**) KEGG pathway enrichment analysis of overlapping genes, highlighting significantly enriched pathways such as NF-κB signaling, oxytocin signaling, cGMP-PKG signaling. Circle size represents the number of genes; color represents the *P*-value.

To elucidate the molecular mechanisms by which miR-2110 modulates endothelial function, we conducted RNA-sequencing to identify downstream target genes and signaling pathways potentially regulated by miR-2110. Transcriptomic profiles were compared between endothelial cells transfected with miR-2110 mimics and mimics NC (Supplementary Table 2). Differential expression analysis using |Log_2_FC| > 0.5 and *P* < 0.05 identified 1217 DEGs, including 809 upregulated and 408 downregulated in the miR-2110 mimics group (Fig. 4A). A heatmap of all DEGs showed clear clustering and separation between groups, with widespread downregulation of gene expression in miR-2110-overexpressing cells (Fig. 4B), consistent with miRNA-mediated post-transcriptional repression.

To identify putative direct targets of miR-2110, we intersected the DEGs with predicted miR-2110 targets from the TargetScan database. This analysis yielded 118 overlapping genes (Fig. 4C), which were subjected to Gene Ontology (GO) and Kyoto Encyclopedia of Genes and Genomes (KEGG) enrichment analyses.

GO enrichment analysis (Fig. 4D) revealed that these genes were involved in biological processes such as response to organophosphorus compounds, axonogenesis, leukocyte differentiation, and cell morphogenesis. Cellular component terms were enriched in synapse, apical cell part, and microfibril, while molecular functions included calcium-activated potassium channel activity, sphingolipid binding, and transferase activity.

KEGG pathway enrichment analysis identified several significantly enriched pathways, including glycosaminoglycan biosynthesis, oxytocin signaling, cell adhesion molecules, cGMP-PKG signaling, ferroptosis, NF-κB signaling, and axon guidance (Fig. 4E). Among those, several have well-established links to CHD, including the cGMP-PKG, oxytocin, and NF-κB signaling pathways, highlighting potential mechanisms by which miR-2110 contributes to CHD pathogenesis. DEGs within these pathways—such as TNF receptor-associated factor 3 (*TRAF3*), phosphoinositide-3-kinase gamma (*PIK3CG*), solute carrier family 8 member 1 (*SLC8A1*), calcium/calmodulin-dependent protein kinase IV (*CAMK4*), etc., were visualized in the heatmap (Fig. 4B). Their KEGG pathway associations were detailed in Supplementary Figs. S2, S3, S4 and Supplementary Table 3.

#### 3.4.2 TRAF3 mRNA expression level is significantly reduced in the miR-2110 overexpressing endothelial cells

**Fig. 5:**
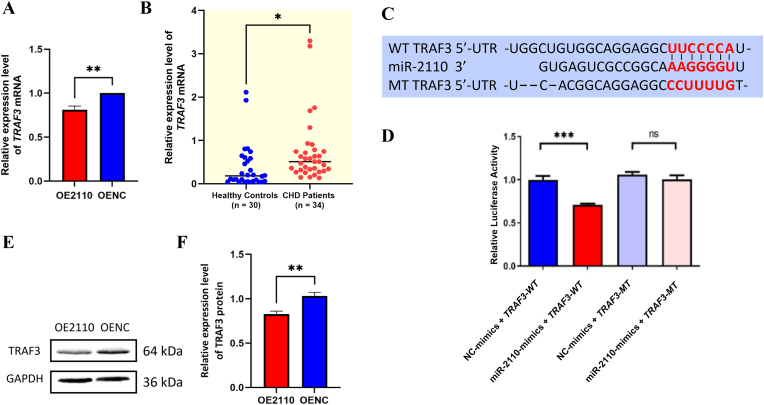
Validation of *TRAF3* as a direct target of miR-2110 by RT-qPCR, luciferase reporter assay, and Western blot analysis (**A**) RT-qPCR analysis of *TRAF3* mRNA levels in OE2110 and OENC cells, calculated using the 2^−ΔΔCt^ method, normalized to GAPDH, and expressed relative to the control group (set to 1). (**B**) *TRAF3* mRNA levels in peripheral blood samples from healthy controls (n = 30) and CHD patients (n = 34), normalized to GAPDH. (**C**) Schematic representation of the predicted binding sites between miR-2110 and the wild-type (WT) 3′-UTR of *TRAF3*, and the corresponding mutated (MT) sequence used for luciferase assays. (**D**) Dual-luciferase reporter assay showing decreased luciferase activity in cells co-transfected with miR-2110 mimics and *TRAF3*-WT but not *TRAF3*-MT. (**E, F**) Western blot analysis and quantification of *TRAF3* protein expression in OE2110 and OENC, with GAPDH as internal reference. ∗*P* < 0.05, ∗∗*P* < 0.01, ∗∗∗*P* < 0.001, ns: not significant. Panels A, D, and F: Data are presented as mean ± SD from three independent biological experiments (N = 3) using EA.hy926 cells. Panel E: Representative blots are shown. Statistical comparisons were performed using unpaired two-tailed Student's t-tests. Panel B (patient samples): Data were presented as median (interquartile range) and were analyzed using the Mann–Whitney *U* test.

RT-qPCR was used to evaluate the expression levels of candidate target genes identified from RNA-sequencing analysis. Notably, among these, *TRAF3* was prioritized for further investigation because it exhibited one of the most pronounced reductions in expression in the miR-2110 mimics group compared with the mimics NC group (P = 0.0068) and is a well-recognized regulator of inflammatory signaling pathways, which are closely implicated in endothelial dysfunction and atherosclerosis (Fig. 5A).

#### 3.4.3 TRAF3 mRNA expression level is significantly increased in patients with CHD

Given that miR-2110 levels were decreased in CHD patients (Fig. 1), we next assessed *TRAF3* mRNA expression in peripheral blood samples. RT-qPCR results showed that *TRAF3* expression was significantly upregulated in CHD patients compared to healthy controls (*P* = 0.0352) (Fig. 5B). These findings align with the reduced *TRAF3* expression observed in miR-2110-overexpressing endothelial cells, supporting an inverse relationship between miR-2110 and *TRAF3* in the context of CHD.

#### 3.4.4 TRAF3 is a direct target of miR-2110 confirmed by dual-luciferase assay and western blot

To verify *TRAF3* as a direct target of miR-2110, we performed a dual-luciferase reporter assay. The wild-type (WT) and mutant (MT) 3′UTR sequences of *TRAF3* (NCBI RefSeq: NM_145725.3) were cloned into the pmirGLO vector and co-transfected with miR-2110 mimics or mimics NC into 293T cells. Luciferase activity was significantly reduced in the miR-2110 + *TRAF3*-WT group compared to NC (*P* = 0.0002), whereas no difference was observed in the miR-2110 + *TRAF3*-MT group (Fig. 5C and D), confirming specific binding.

Consistently, Western blot analysis showed that TRAF3 protein levels were significantly decreased in miR-2110-overexpressing endothelial cells compared to the control (*P* = 0.0078) (Fig. 5E and F). Together, these results confirm that *TRAF3* is a direct post-transcriptional target of miR-2110.

### 3.5 *TRAF3* is upregulated in atherosclerotic mice, supporting the involvement of the mir-2110/*TRAF3* axis in CHD

*In vitro*, we demonstrated that miR-2110 directly represses *TRAF3*, potentially modulating endothelial cell function *via* NF-κB signaling and thereby contributing to CHD pathogenesis. To extend these findings *in vivo*, we employed ApoE^−/−^ mice fed a high-fat diet (HFD) — a widely used atherosclerosis and CHD model that closely recapitulates endothelial dysfunction, vascular inflammation, and plaque formation in human CHD [19]. Given that miR-2110 is primate-specific (according to miRBase https://www.mirbase.org/; accessed June 2025) and no annotated homologous sequence has been reported in mice or other laboratory animals, direct *in vivo* assessment is unfeasible. Therefore, we examined *TRAF3*, which was validated as the direct target of miR-2110, as a downstream molecule relevant to atherosclerosis, independent of miR-2110 regulation *in vivo*. Hypothesically, if *Traf3* is upregulated in murine atherosclerotic lesions, it suggests that a similar elevation may occur in human CHD and indicates that TRAF3 is subject to disease-associated dysregulation. In this context, TRAF3 upregulation is compatible with reduced miR-2110 activity as one of several regulatory inputs in human setting, consistent with our previous findings, and supports the biological relevance of the miR-2110/TRAF3 relationship in CHD.

#### 3.5.1 ApoE^−/−^ HFD mice showed clear atherosclerosis plaque formation inside vascular of aorta and heart tissue

**Fig. 6:**
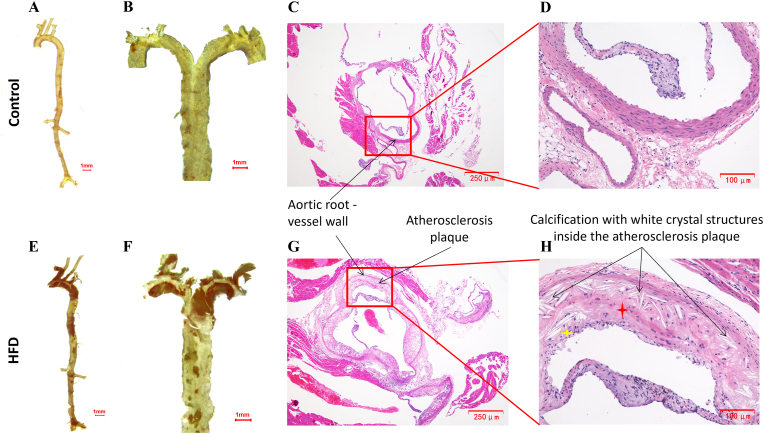
Oil Red O and histological staining confirm atherosclerotic lesions in ApoE^−/−^ mice fed a high-fat diet (**A, B**) Oil Red O staining of the whole aorta and *en face* preparations from control mice showing smooth, lesion-free vascular surfaces. (**C, D**) Hematoxylin and eosin (H&E) staining of the aortic valve region in control mice showing preserved vascular architecture without pathological changes (C, 40 × ; D, 100 × ). (**E, F**) Oil Red O staining of the whole aorta and *en face* preparations from ApoE^−/−^ HFD mice demonstrating extensive lipid-rich plaques, particularly in the aortic arch and thoracic regions. (**G, H**) H&E staining of the aortic valve region in ApoE^−/−^ HFD mice revealing advanced atherosclerotic lesions with marked intimal thickening, inflammatory cell infiltration (yellow asterisk), fibrosis (red asterisk), and calcification characterized by white crystalline deposits within the plaque (black arrows) (G, 40 × ; H, 100 × ). Data represent n = 6 mice per group.

To validate the atherosclerosis model, vascular pathology was assessed in control and ApoE^−/−^ HFD mice. Oil Red O staining, a lipid-specific dye used to identify lipid-rich areas and visualize atherosclerotic plaques, revealed clean and intact vascular surfaces without visible lesions in the entire aorta (Fig. 6A) and *en face* staining of control mice (Fig. 6B). H&E staining of the aortic valve region confirmed preserved vascular architecture without pathological changes (Fig. 6C and D). In contrast, ApoE^−/−^ HFD mice developed extensive lipid-rich plaques in the aortic arch and thoracic regions, as demonstrated by Oil Red O staining of the whole aorta (Fig. 6E) and *en face* images (Fig. 6F). H&E staining of the aortic valve area revealed classical features of advanced atherosclerosis, including marked intimal thickening, inflammatory cell infiltration, fibrosis, and prominent vascular calcification characterized by crystalline deposits (Fig. 6G and H). These histopathological findings confirmed the successful establishment of advanced atherosclerotic lesions in the ApoE^−/−^ HFD mice model.

#### 3.5.2 TRAF3 increased expression in cardiac and vascular tissues of ApoE^−/−^ HFD mice

**Fig. 7:**
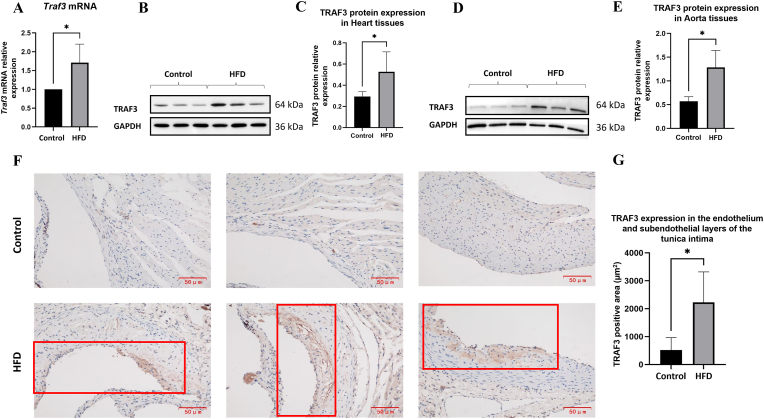
Increased *TRAF3* expression in cardiac and aortic tissues of ApoE^−/−^ mice fed a high-fat diet (**A**) Relative *Traf3* mRNA expression in the aortic root and adjacent heart tissue quantified by RT-qPCR, calculated using the 2^−ΔΔCt^ method, normalized to GAPDH, and expressed relative to the control group (set to 1). (**B, C**) Western blot analysis and quantification of TRAF3 protein expression in heart tissues. (**D, E**) Western blot analysis and quantification of TRAF3 protein expression in aortic tissues. (**F**) Representative immunohistochemical staining of TRAF3 in aortic root sections from control and ApoE^−/−^ HFD mice, showing predominant localization in the tunica intima, including the endothelial layer and subendothelial regions containing vascular smooth muscle cells and infiltrating leukocytes (200 × ). Red boxes highlight regions with enhanced staining in HFD mice. (G) Quantification of TRAF3-positive area in the tunica intima. ∗*P* < 0.05. Data represent mean ± SD; n = 6 mice per group. Statistical comparisons were performed using unpaired two-tailed Student's t-tests.

Total RNA was extracted from the aortic root region, including adjacent upper cardiac tissue, which was dissected *en bloc* from the hearts of ApoE^−/−^ HFD mice and control mice. This region was selected because it is a well-established site for atherosclerotic lesion development in murine models of CHD. RT-qPCR analysis revealed a 1.7-fold increase in *TRAF3* mRNA levels in the ApoE^−/−^ HFD group compared with controls (*P* = 0.0213; Fig. 7A). Western blotting demonstrated significantly elevated TRAF3 protein levels in both heart (Fig. 7B and C) and aorta tissues (Fig. 7D and E) of ApoE^−/−^ HFD mice (*P* = 0.0491 and P = 0.0409, respectively).

Immunohistochemistry demonstrated increased TRAF3 expression predominantly localized in the tunica intima, encompassing the endothelial layer and subendothelial regions containing vascular smooth muscle cells and infiltrating leukocytes, with weaker staining observed in the tunica media (Fig. 7F). Quantification confirmed a significantly greater TRAF3-positive area in the HFD group (*P* = 0.0249; Fig. 7G).

Taken together, these observations are consistent with the hypothesis that *TRAF3* expression is upregulated in atherosclerotic lesions, supporting the potential involvement of the miR-2110/*TRAF3* regulatory axis in the pathogenesis of CHD.

## 4 Discussion

Coronary heart disease remains a major cause of morbidity and mortality worldwide, emphasizing the urgent need to identify novel molecular biomarkers and therapeutic targets. In this study, we provided evidence supporting a previously unrecognized role of miR-2110 as a potential regulator of endothelial cell function with relevance to atherosclerosis. Our data demonstrate that miR-2110 is significantly downregulated in CHD patients and directly targets TRAF3, a multifunctional adaptor protein involved in inflammatory signaling [20]. The inverse regulation of miR-2110 and *TRAF3* was validated *in vitro* and observed in human samples. In the experimental atherosclerosis model, only *TRAF3* upregulation could be assessed, which is compatible with reduced miR-2110 activity as one of several regulatory inputs but cannot be directly tested because miR-2110 lacks a murine homolog. Together, these findings suggest that this axis may be involved in endothelial dysfunction and may contribute to the pathogenesis of CHD.

It is important to note that *TRAF3* upregulation in atherosclerotic conditions may arise from multiple upstream mechanisms, including transcriptional activation, inflammatory signaling, or other post-transcriptional regulation. Accordingly, elevated *TRAF3* levels cannot be attributed solely to the downregulation of miR-2110. Nevertheless, given that miR-2110 directly targets and suppresses *TRAF3* expression, reduced miR-2110 availability may constitute one regulatory layer that permits or facilitates *TRAF3* accumulation under disease conditions. In this context, the concurrent observation of decreased miR-2110 and increased *TRAF3* in CHD patients is more consistent with a coordinated dysregulation of the miR-2110/*TRAF3* regulatory axis rather than a direct one-to-one causal relationship. Although loss-of-function or rescue experiments would provide additional causal evidence, the combination of direct target validation, reverse expression patterns in human samples, and consistent *TRAF3* upregulation in atherosclerotic lesions supports the relevance of the miR-2110/*TRAF3* regulatory relationship in CHD. Alternative explanations, such as transcriptional activation of *TRAF3* by inflammatory stimuli or regulation by other miRNAs, cannot be excluded and likely coexist in the complex atherosclerotic milieu.

The expression of miR-2110 was assessed in peripheral blood, which served as a practical surrogate when direct endothelial sampling is not feasible. However, circulating miRNAs represent a heterogeneous pool derived from multiple cellular sources, including leukocytes, platelets, erythrocytes, and extracellular vesicles [21], and therefore primarily provide associative clinical information rather than cell-type–specific mechanistic insight. In the present study, blood-based miR-2110 profiling was used to identify a clinically relevant alteration associated with CHD, without implying direct endothelial origin or uptake *in vivo*. Based on the well-established contribution of endothelial dysfunction to CHD pathogenesis, we subsequently investigated whether miR-2110 itself is capable of regulating endothelial cell behavior under controlled experimental conditions. The observed effects of miR-2110 modulation on endothelial proliferation, migration, apoptosis, cell-cycle progression, and senescence demonstrate that miR-2110 has the capacity to regulate key endothelial phenotypes *in vitro*, independent of assumptions regarding its cellular source in the circulation.

Our findings indicate that miR-2110 may exerts a context- and dosage-dependent regulatory effect on endothelial cell behavior. Under controlled *in vitro* conditions, forced overexpression of miR-2110 led to reduced proliferative and migratory activity, S-phase cell-cycle arrest, decreased apoptosis, and enhanced cellular senescence, reflecting a shift toward a low-proliferative, senescence-prone endothelial phenotype with diminished regenerative capacity. An apparent paradox arises from these findings in that supraphysiological overexpression of miR-2110 in endothelial cells induced a dysfunctional phenotype, whereas circulating miR-2110 levels were reduced in CHD patients, which may indicate a protective role of miR-2110 in CHD. However, it is important to notice that these experimental conditions were designed to interrogate the functional consequences of excessive miR-2110 activity and do not model the endogenous regulation of miR-2110 in CHD. These observations suggest that endothelial homeostasis may depend on a finely tuned regulatory range of miR-2110 activity, in which deviations in either direction can disrupt cellular balance and function. This discrepancy underscores the context-dependent and non-linear nature of miRNA-mediated regulation. Importantly, reduced circulating miR-2110 levels in CHD should be interpreted as a disease-associated biomarker rather than a direct indicator of endothelial miR-2110 activity *in vivo*.

Such a dual outcome can help explain this apparent paradox and underscores the importance of dosage and cellular context in miRNA-mediated regulation. Similar biphasic effects have been described for other endothelial miRNAs, which can either promote or inhibit vascular repair depending on their expression levels. For instance, miR-155 can either promote or inhibit angiogenesis depending on its abundance [[22], [23], [24], [25]], miR-21 exhibits both protective and pathogenic roles in regulating endothelial survival and inflammation [26], miR-126 can promote vascular integrity at physiological levels but drive pathological remodeling when dysregulated [27], and miR-34a is upregulated in atherosclerotic endothelium, where it promotes apoptosis and senescence *via* targeting of HDAC1, yet its inhibition has been shown to restore endothelial viability and suppress apoptosis [28,29]. By analogy, miR-2110 appears to operate within a narrow functional window to preserve endothelial homeostasis by maintaining a balance between survival, proliferation, and senescence. Disruption of this balance—whether by loss or excess—may contribute to vascular dysfunction and atherosclerosis progression.

RNA-seq and pathway enrichment analysis provided insights into the potential downstream effects of miR-2110. Beyond *TRAF3*, predicted pathways included the cGMP–PKG signaling pathway, which is cardioprotective and clinically targeted by drugs such as sacubitril/valsartan [30], and the oxytocin signaling pathway, previously shown to reduce infarct size and promote cardiac recovery after ischemia–reperfusion [31]. These associations expand the potential influence of miR-2110 on stress and inflammation-related signaling. While enrichment of other cardioprotective pathways was observed, their functional significance in the context of miR-2110 requires further validation. However, given the established role of NF-κB in driving atherosclerotic inflammation [32], and the known regulation of this pathway by *TRAF3* [20,33], we propose that the miR-2110/*TRAF3* axis may be associated with NF-κB–related inflammatory signaling in the context of CHD. Direct assessment of NF-κB activation would further strengthen the mechanistic interpretation; therefore, NF-κB signaling is discussed here as a biologically plausible downstream context rather than a directly validated pathway.

Functional validation later confirmed *TRAF3* as a direct target of miR-2110 by dual-luciferase reporter assay and Western blotting. TRAF3 is a TNF receptor–associated adaptor protein that participates in innate and inflammatory signaling cascades. In the context of vascular injury and atherosclerosis, TRAF3 expression is consistently elevated in atherosclerosis lesions and has been linked to chronic inflammatory responses [34]. For instance, studies have reported *TRAF3* upregulation in atherosclerotic plaques and showed that *TRAF3* deletion reduced infarct size and inflammation through inhibition of NF-κB and JNK signaling [35]. Consistent with these reports, *TRAF3* was markedly increased in aortic lesions of our ApoE^−/−^ mice. Together with our molecular data demonstrating miR-2110 targeting of *TRAF3*, these observations support a model in which miR-2110 downregulation permits *TRAF3* accumulation, potentially contributing to endothelial activation and sustained vascular inflammation through NF-κB–related signaling in CHD. Further clarification of this regulatory pathway will require targeted functional studies, for instance, endothelial-specific *TRAF3* knockdown in mice, direct modulation of miR-2110 in suitable *in vivo* models, and assessment of NF-κB-related signaling.

In line with the concept that *TRAF3* is subject to complex post-transcriptional regulation, several other miRNAs have also been reported to directly target *TRAF3* in different pathological contexts. For example, miR-361-5p has been shown to bind *TRAF3* and modulate cell proliferation and apoptosis through the NF-κB pathway in ovarian cancer, as validated by dual-luciferase reporter assays and expression analyses [36]. Similarly, miR-1307-5p was identified as a direct regulator of *TRAF3* in lung adenocarcinoma, where miR-1307-5p–mediated *TRAF3* repression led to activation of NF-κB/MAPK signaling and promoted tumor growth [37]. These observations suggest that *TRAF3* may act as a shared regulatory node for multiple miRNAs, with context-dependent functional consequences. In this context, the miR-2110/TRAF3 interaction identified in the present study should be viewed as one regulatory component within a broader miRNA-mediated network rather than an exclusive mechanism controlling *TRAF3* expression in CHD. Accordingly, the endothelial phenotypes observed following miR-2110 modulation may reflect the combined effects of multiple downstream targets and broader transcriptional changes under *in vitro* conditions, rather than a single linear pathway.

Several limitations should be acknowledged. First, patient samples were recruited from a single center, which may introduce selection bias and limit the generalizability of our findings. In addition, detailed information on medication use, smoking status, and disease severity was not available, which may influence miR-2110 and *TRAF3* expression *via* vascular inflammation and endothelial dysfunction. Second, circulating miR-2110 originates from multiple cellular sources; thus, its reduction may be clinically relevant but does not imply direct endothelial regulation *in vivo*. Third, limitations inherent to the murine model should be acknowledged. Lesions in ApoE^−/−^ mice predominantly occur in the aortic root rather than the coronary arteries, thus only partially recapitulating human CHD. Moreover, miR-2110 is a primate-specific miRNA without a direct homolog in mice, restricting functional modeling in standard murine systems. Consequently, our *in vivo* data primarily capture *TRAF3* dysregulation rather than the direct function of miR-2110. Fourth, although our results highlight a potential role for the miR-2110/*TRAF3* axis in CHD pathogenesis, loss-of-function approaches, functional rescue experiments and longitudinal studies are still needed to establish causality and therapeutic relevance. In addition, direct assessment of downstream NF-κB activation was not performed in this study and therefore NF-κB involvement is discussed based on pathway analysis and prior knowledge. Finally, normalization of RT-qPCR data relied on single internal controls (U6 for miRNA and *GAPDH* for mRNA), which could introduce measurement bias; inclusion of additional reference genes would strengthen the robustness of gene expression analyses.

Despite these limitations, the study has several strengths. It combines clinical observations with transcriptomic analysis, mechanistic *in vitro* experiments in endothelial cells, and *in vivo* assessment of *TRAF3* expression in an experimental atherosclerosis model, thereby reducing reliance on any single experimental approach. In addition, the identification of *TRAF3* as a direct target of miR-2110 provides a mechanistic link between altered miRNA expression and inflammatory signaling relevant to endothelial dysfunction. Finally, by considering the context- and dosage-dependent effects of miR-2110, the study adopts a cautious interpretation of miRNA function in CHD.

Several aspects should be addressed in future studies. Endothelial‐specific loss‐ and gain‐of‐function approaches, including miR-2110 inhibition or rescue experiments, will help clarify the role of endogenous miR-2110 in endothelial biology and atherosclerosis. Validation in primary human endothelial cells, as well as in alternative experimental systems such as humanized *in vivo* models, would further support the physiological relevance of the present findings. In addition, direct assessment of downstream inflammatory signaling, particularly NF-κB–related pathways, will be important to strengthen the mechanistic link between miR-2110 dysregulation, TRAF3 accumulation, and endothelial dysfunction. Finally, larger and longitudinal clinical cohorts with detailed phenotypic characterization are needed to determine whether circulating miR-2110 has diagnostic or prognostic value across different stages of CHD.

## 5 Conclusions

Collectively, our findings suggest that the miR-2110/*TRAF3* interaction may constitute a previously unrecognized regulatory axis in CHD, potentially relevant to endothelial dysfunction and disease pathogenesis.

## Ethics statement

All participants provided written informed consent. The study protocol was approved by the Ethics Committee of Guangxi Medical University (Approval No. 2019-SB-060) and conducted in accordance with the 1975 Declaration of Helsinki. All experimental procedures involving animals were approved by the Animal Care and Welfare Committee of Guangxi Medical University (Approval No. 202410097) and complied with the Guideline for Ethical Review of Animal Welfare (China National Standard GB/T 35892-2018).

## Funding

This research was funded by the 10.13039/501100001809National Natural Science Foundation of China, grant numbers 82360286, 81960266, and 32060188.

## CRediT authorship contribution statement

**ThanhLoan Tran:** Conceptualization, Data curation, Formal analysis, Investigation, Methodology, Writing – original draft, Writing – review & editing. **Zhong-Yu Wang:** Methodology, Writing – original draft. **Pei-Shan Li:** Conceptualization, Methodology. **Ying Yang:** Conceptualization, Methodology. **Yi-Wei Zhang:** Data curation, Software. **Shu-Ming Zhang:** Investigation, Validation. **PhongSon Dinh:** Visualization. **NgocLong Le:** Data curation. **TrungHieu Pham:** Formal analysis. **Ling Huang:** Project administration. **Ning-Yuan Chen:** Project administration. **Jun-Hua Peng:** Supervision. **Shang-Ling Pan:** Funding acquisition, Project administration, Supervision, Writing – review & editing.

## Declaration of competing interest

The authors declare that they have no known competing financial interests or personal relationships that could have appeared to influence the work reported in this paper.

## Data Availability

The processed RNA-seq data supporting the findings of this study are available in the Zenodo repository at https://doi.org/10.5281/zenodo.18032014.
