# Supplementary material for: The miR-2110/TRAF3 axis is associated with endothelial dysfunction and atherosclerosis in coronary heart disease
Source: Biochem Biophys Rep. 2026 Feb 20;45:102508. doi: 10.1016/j.bbrep.2026.102508 (PMC12937024; doi:10.1016/j.bbrep.2026.102508)
Supplement: Multimedia component 1 [file mmc1.docx]

**Supplementary Table 1. Weekly body weight of mice.**

| **ApoE-/- Mouse** | **Mice Weight (g)** | | | | | | | | | | | | | | | | | | | |
| --- | --- | --- | --- | --- | --- | --- | --- | --- | --- | --- | --- | --- | --- | --- | --- | --- | --- | --- | --- | --- |
|  | **At beginning** | **Week 0** | **Mean ± SD (week 0)** | **t** | ***P* value** | **Week 1** | **Week 2** | **Week 3** | **Week 4** | **Week 5** | **Week 6** | **Week 7** | **Week 8** | **Week 9** | **Week 10** | **Week 11** | **Week 12** | **Mean ± SD (week 12)** | **t** | ***P* value** |
| HFD 1 | 24.8 | **25.2** | 24.20 ± 0.597 | 0.2154 | 0.8338 | 26.7 | 27.7 | 29 | 29.3 | 30.4 | 29.9 | 30 | 30.1 | 29.6 | 30.2 | 30.8 | **31.2** | 31.43 ± 2.169 | 0.3215 | 0.7544 |
| HFD 2 | 23.9 | **23.6** |  |  |  | 24.4 | 26.2 | 27.8 | 29.8 | 31 | 32 | 32 | 31.9 | 31.8 | 33.1 | 33 | **32.4** |  |  |  |
| HFD 3 | 23 | **23.7** |  |  |  | 24.3 | 24.5 | 25.1 | 26.1 | 27.1 | 27 | 27.8 | 27.2 | 26.7 | 27.9 | 26.5 | **27.2** |  |  |  |
| HFD 4 | 24.3 | **24.4** |  |  |  | 24.8 | 25.9 | 26.9 | 28.3 | 30.1 | 30.1 | 31.3 | 30.5 | 30 | 31.8 | 32.9 | **32.9** |  |  |  |
| HFD 5 | 22.7 | **23.9** |  |  |  | 25.9 | 26.9 | 27.5 | 28.5 | 29.7 | 30.1 | 30.9 | 31.1 | 31.2 | 31.8 | 32.2 | **32** |  |  |  |
| HFD 6 | 23.7 | **24.4** |  |  |  | 25.2 | 26.1 | 28.4 | 29 | 29.6 | 30.5 | 30.5 | 30 | 29.7 | 30.7 | 30.5 | **32.9** |  |  |  |
| Control 1 | 23.6 | **24.7** | 24.12 ± 0.736 |  |  | 25.6 | 27.2 | 28.9 | 30.7 | 31.4 | 32.2 | 33 | 33.1 | 33.7 | 34.9 | 35.8 | **35.6** | 31.87 ± 2.488 |  |  |
| Control 2 | 24.9 | **25.1** |  |  |  | 26.4 | 27.3 | 27.3 | 27.8 | 28.3 | 28.3 | 29.7 | 29.8 | 30.4 | 30.2 | 30.4 | **31.1** |  |  |  |
| Control 3 | 23.3 | **23.9** |  |  |  | 24.7 | 26.4 | 27.3 | 27.9 | 28.9 | 29.1 | 30.6 | 30.8 | 31.3 | 31.2 | 31.1 | **32** |  |  |  |
| Control 4 | 22.6 | **23** |  |  |  | 23.9 | 25.9 | 27.8 | 28.9 | 30.5 | 30.1 | 31.1 | 31 | 31.6 | 31.6 | 32.8 | **33.8** |  |  |  |
| Control 5 | 23.4 | **23.8** |  |  |  | 24.2 | 25.7 | 26.3 | 26.8 | 27.8 | 27.2 | 27.9 | 27.2 | 27.3 | 27.8 | 28 | **29.4** |  |  |  |
| Control 6 | 23.7 | **24.2** |  |  |  | 24.9 | 25.9 | 26.3 | 26.7 | 27.2 | 27.8 | 28.8 | 28.5 | 27.8 | 28.2 | 29.1 | **29.3** |  |  |  |

- At the baseline, after 1 week of acclimatation (Week 0): the HFD group weighed 24.20 ± 0.597 g, while the Control group weighed 24.12 ± 0.736 g. The *P* value = 0.8338 indicates that the difference was not statistically significant.
🡺 This confirms that the two groups were homogeneous in terms of body weight at the start of the experiment, eliminating initial bias.

- At the endpoint (Week 12): the HFD group weighed 31.43 ± 2.169 g, while the Control group weighed 31.87 ± 2.488 g. The *P* value = 0.7544 indicates that the difference in final body weight was not statistically significant. Both groups gained weight to a nearly identical extent.
🡺 The lack of a significant difference in body weight between the two groups allows us to exclude body weight or obesity as a confounding factor. Therefore, any observed differences in atherosclerotic plaque development can be directly attributed to the effects of the high-fat diet on lipid metabolism.

**Supplementary Table 2. Complete RNA-seq mRNA expression dataset. (Excel file)**

**Supplementary Table 3. Genes and signaling pathways from KEGG enrichment analysis.**

| **GENE IDs** | **Log2FC** | ***P* value** | **Pathway** | **Protein name in the KEGG pathway** |
| --- | --- | --- | --- | --- |
| KCNJ8 | -6.530086122 | 0.000159334 | cGMP-PKG signaling pathway | mKATP |
| ITGA9 | -5.959052187 | 0.002554994 | Cell adhesion molecules | Integrin α9 |
| NCAM1 | -2.854953622 | 0.033838687 | Cell adhesion molecules | NCAM |
| SLC8A1 | -2.261848246 | 2.60188E-06 | cGMP-PKG signaling pathway | NCX |
| SEMA5A | -1.765680034 | 0.000180572 | Axon guidance | Sema5A |
| LYN | -1.346811349 | 4.45364E-12 | NF-kappa B signaling pathway | Lyn |
| CHST11 | -1.330436053 | 0.013938466 | Glycosaminoglycan biosynthesis - chondroitin sulfate / dermatan sulfate | C4ST |
| PDE3A | -1.075546804 | 6.62201E-05 | cGMP-PKG signaling pathway | PDE3 |
| PPP1R12B | -1.025916768 | 0.036938869 | Oxytocin signaling pathway | MLCP |
| SLC7A11 | -0.964442521 | 1.84906E-08 | Ferroptosis | xCT |
| PLXNA4 | -0.946133283 | 0.002303698 | Axon guidance | Plexin-A4 |
| NGEF | -0.917132501 | 0.004475304 | Axon guidance | Ephexin1 (NGEF) |
| TRAF3 | -0.854116178 | 0.017553616 | NF-kappa B signaling pathway | TRAF3 |
| UNC5B | -0.846657638 | 8.72472E-05 | Axon guidance | Unc5B |
| SELP | -0.75897767 | 0.047248183 | Cell adhesion molecules | P-selectin |
| CHST3 | -0.671359461 | 1.31798E-07 | Glycosaminoglycan biosynthesis - chondroitin sulfate / dermatan sulfate | C6ST |
| STEAP3 | -0.636487059 | 0.006774526 | Ferroptosis | STEAP3 |
| PIK3CG | -0.605483755 | 0.021537201 | Oxytocin signaling pathway | PI3Kγ |
| PIK3CG | -0.605483755 | 0.021537201 | cGMP-PKG signaling pathway | PI3Kγ |
| KCNJ14 | -0.604304174 | 0.00346922 | Oxytocin signaling pathway | GIRK |
| ERC1 | -0.576279 | 0.011786671 | NF-kappa B signaling pathway | ELKS |
| CAMK4 | -0.575261718 | 0.018172226 | Oxytocin signaling pathway | CaMKIV |
| CLDN1 | -0.507763399 | 0.001256806 | Cell adhesion molecules | Claudin-1 |
