# Supplementary material for: The miR-2110/TRAF3 axis is associated with endothelial dysfunction and atherosclerosis in coronary heart disease
Source: Biochem Biophys Rep. 2026 Feb 20;45:102508. doi: 10.1016/j.bbrep.2026.102508 (PMC12937024; doi:10.1016/j.bbrep.2026.102508)
Supplement: Multimedia component 3 [file mmc3.docx]

**SUPPLEMENTARY FIGURES**


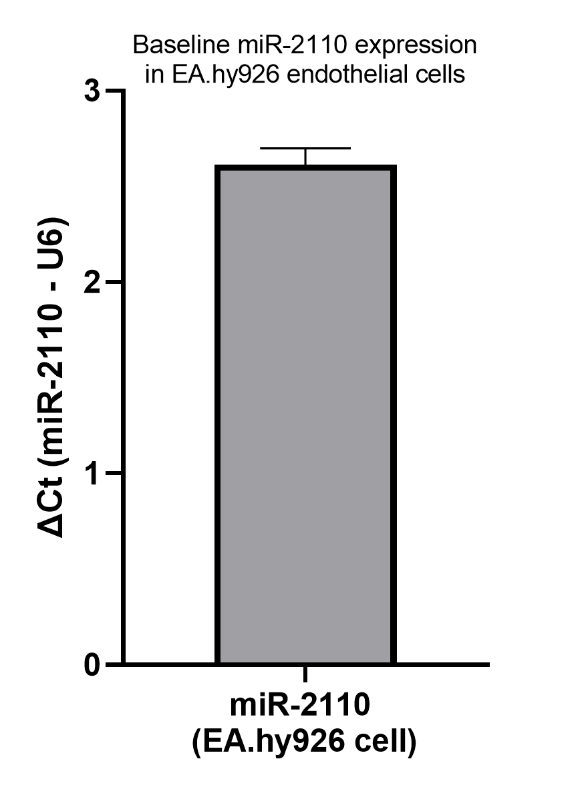


**Supplementary Figure S1.** Baseline expression of miR-2110 in EA.hy926 endothelial cells. miR-2110 levels were measured by RT-qPCR and normalized to U6 using the ΔCt method. Data are presented as mean ± SD from three independent biological replicates.

As can be seen from the figure, miR-2110 exhibited a baseline ΔCt value of approximately 2.6 relative to U6, indicating moderate endogenous expression in EA.hy926 cells.

**
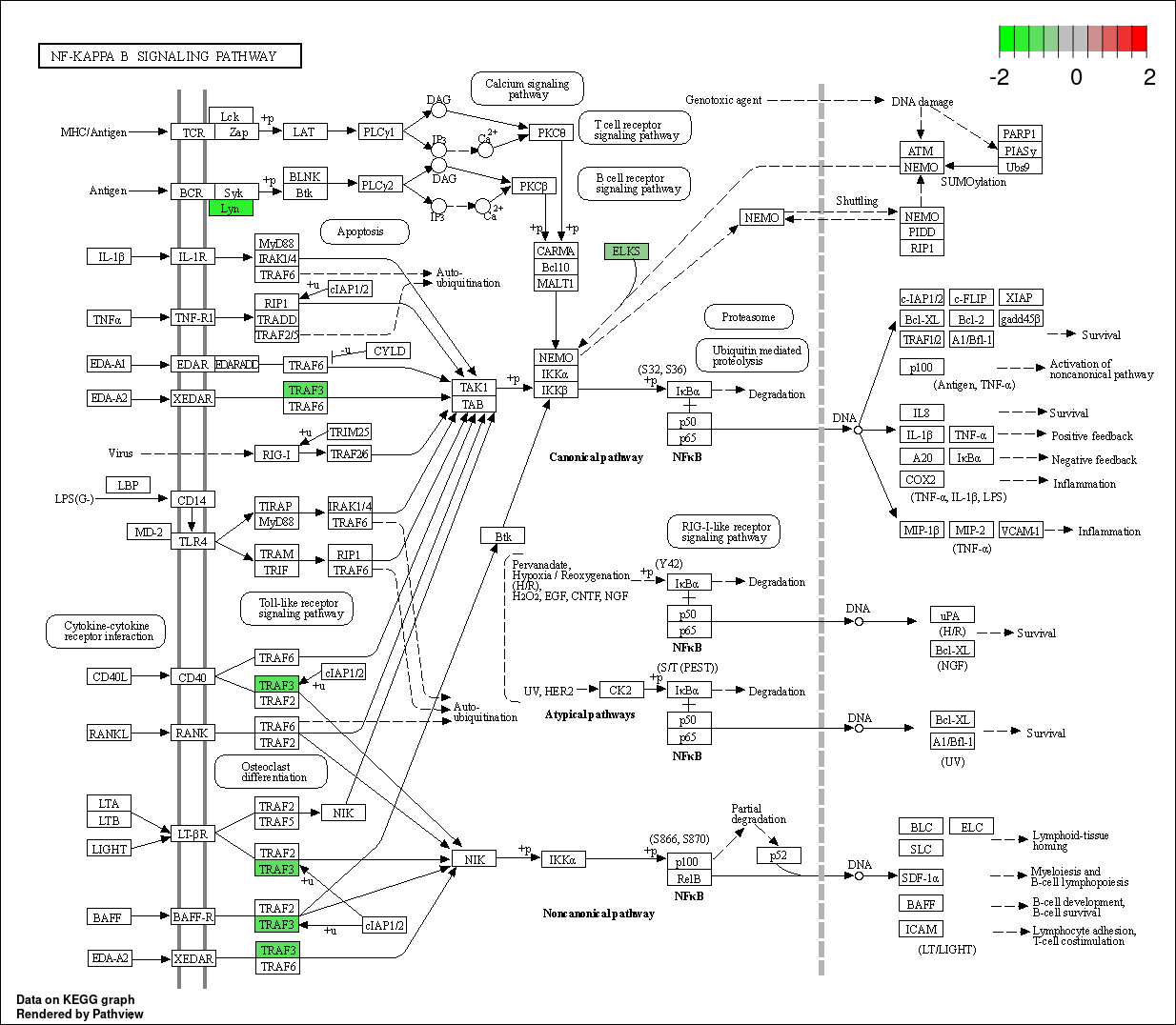
Supplementary Figure 2.** The NF – κB signaling pathway

**Note:** Protein encoded by the potential target genes in the signaling pathways are highlighted. The intensity of the colors represents the level of differential expression of genes. Green color represents lower expression levels or downregulation.


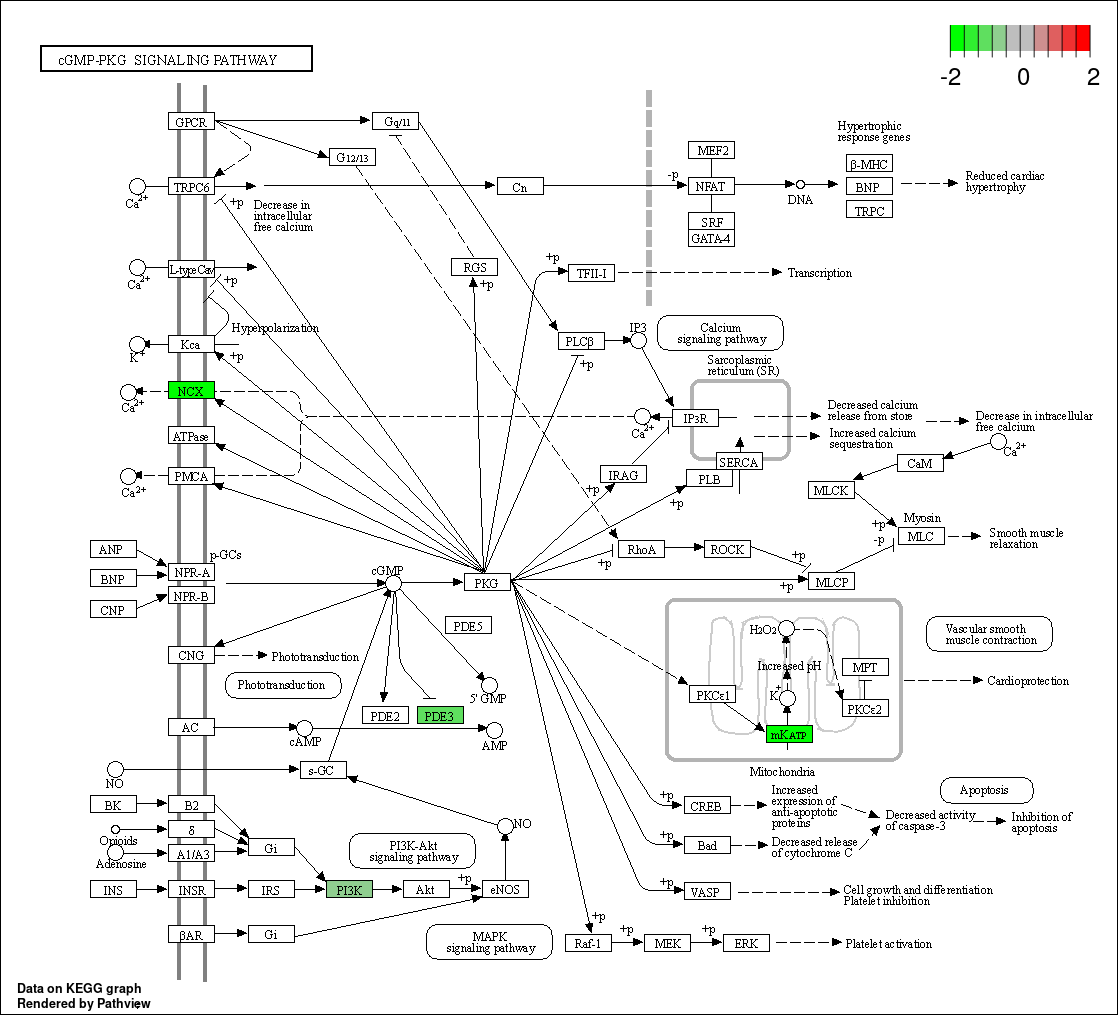


**Supplementary Figure 3.** The cGMP-PKG signaling pathway

**Note:** Protein encoded by the potential target genes in the signaling pathways are highlighted. The intensity of the colors represents the level of differential expression of genes. Green color represents lower expression levels or downregulation.

**
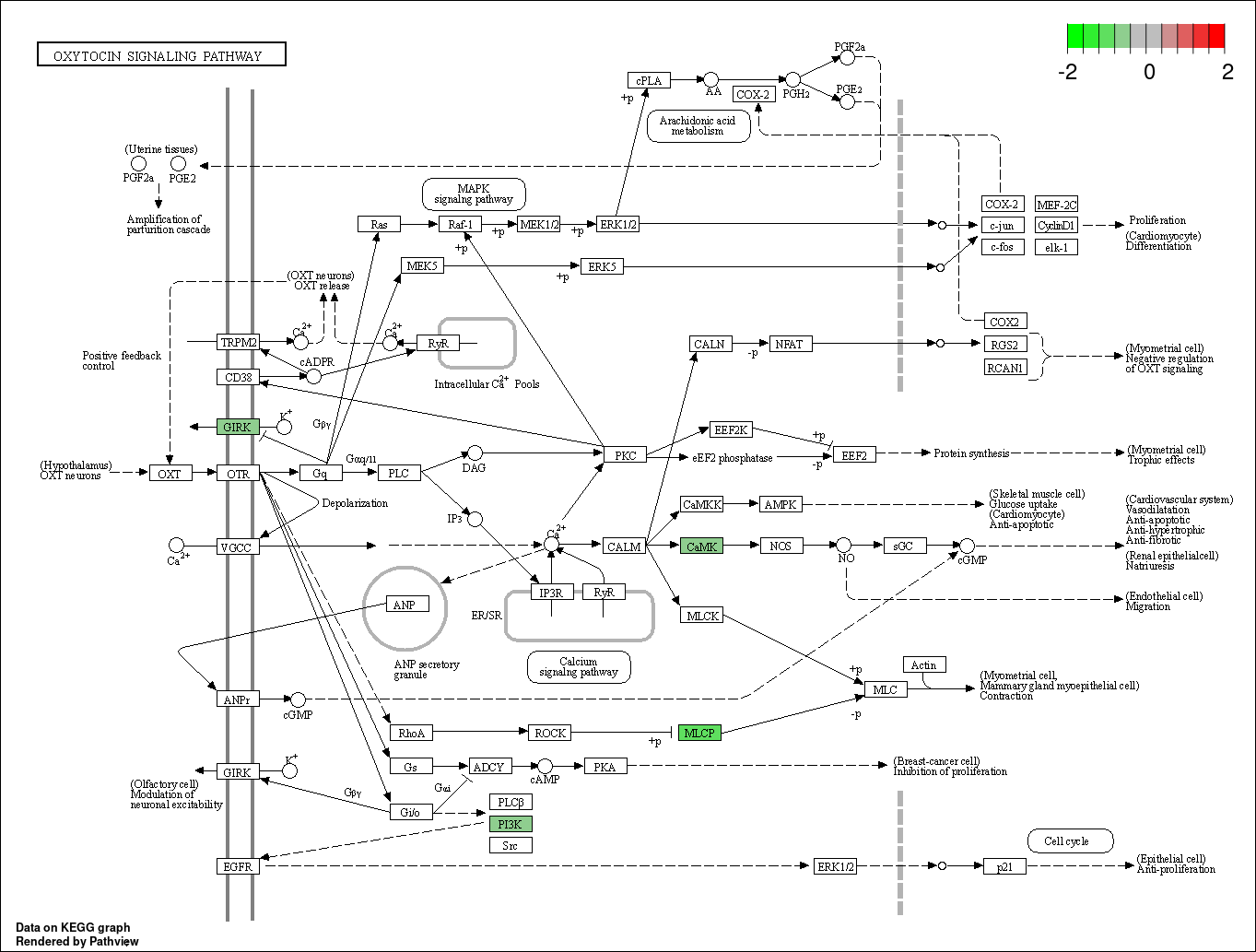
Supplementary Figure 4.** The Oxytocin signaling pathway

**Note:** Protein encoded by the potential target genes in the signaling pathways are highlighted. The intensity of the colors represents the level of differential expression of genes. Green color represents lower expression levels or downregulation.
