# Supplementary figures and images for: The miR-2110/TRAF3 axis is associated with endothelial dysfunction and atherosclerosis in coronary heart disease
Source: Biochem Biophys Rep. 2026 Feb 20;45:102508. doi: 10.1016/j.bbrep.2026.102508 (PMC12937024; doi:10.1016/j.bbrep.2026.102508)

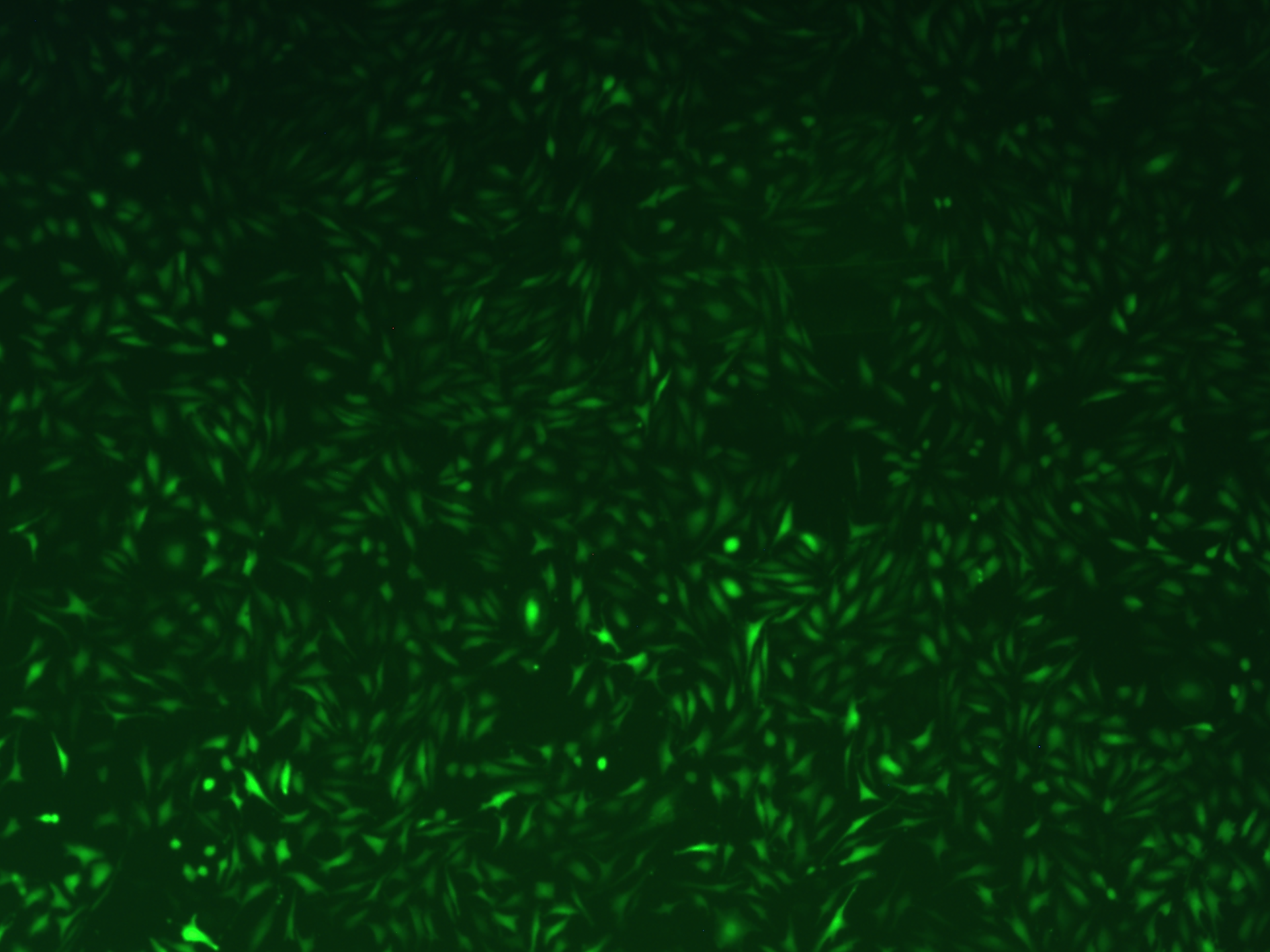

Supplement: Multimedia component 4 [file mmc4.zip › Original Figures/Figure 2. Microscopy images/Figure 2A. Fluorescence OE 2110.tif]

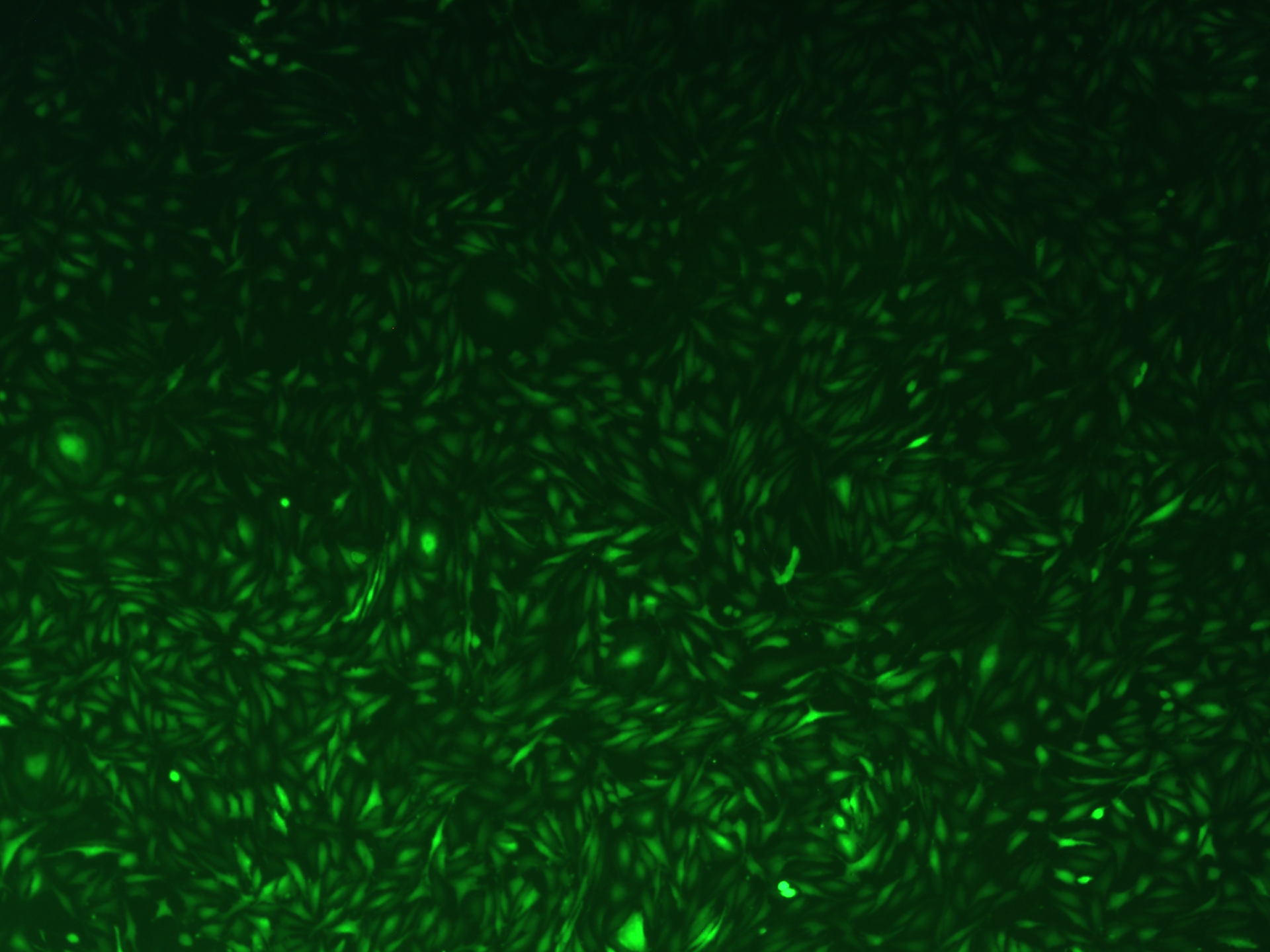

Supplement: Multimedia component 4 [file mmc4.zip › Original Figures/Figure 2. Microscopy images/Figure 2A. Fluorescence OE NC.tif]

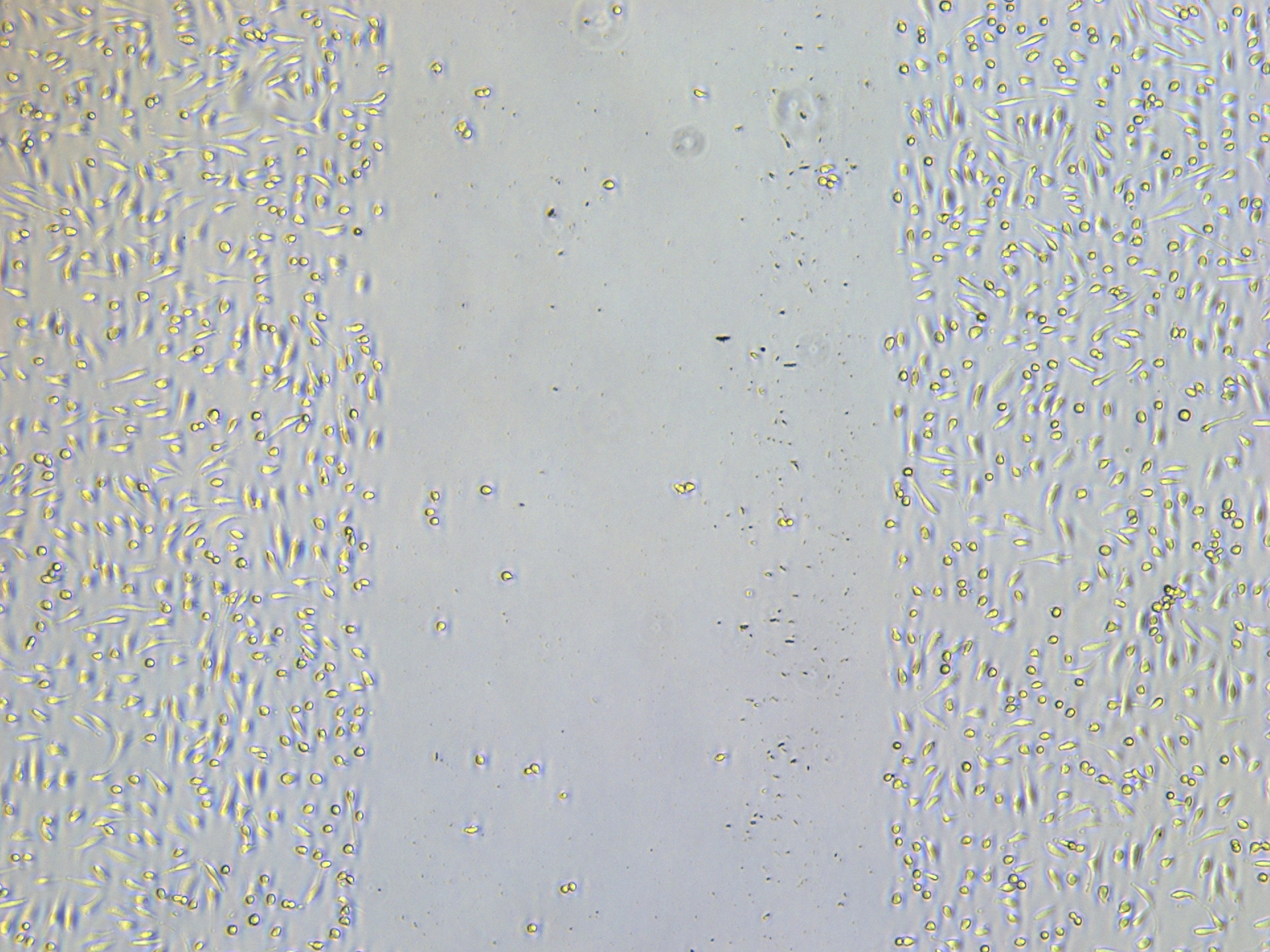

Supplement: Multimedia component 4 [file mmc4.zip › Original Figures/Figure 2. Microscopy images/Figure 2E. Scratch test - OE2110 - 0h.jpg]

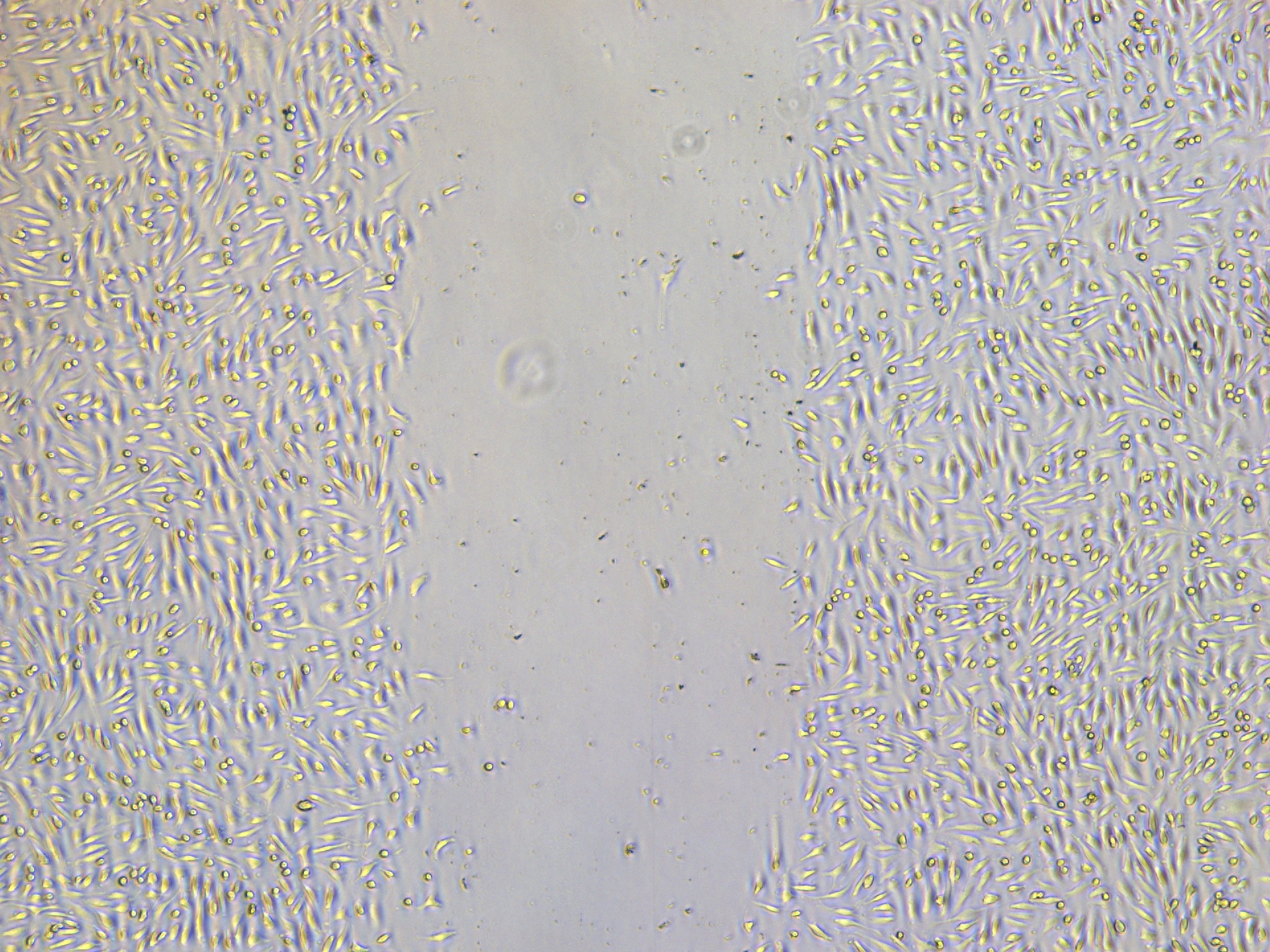

Supplement: Multimedia component 4 [file mmc4.zip › Original Figures/Figure 2. Microscopy images/Figure 2E. Scratch test - OE2110 - 12h.jpg]

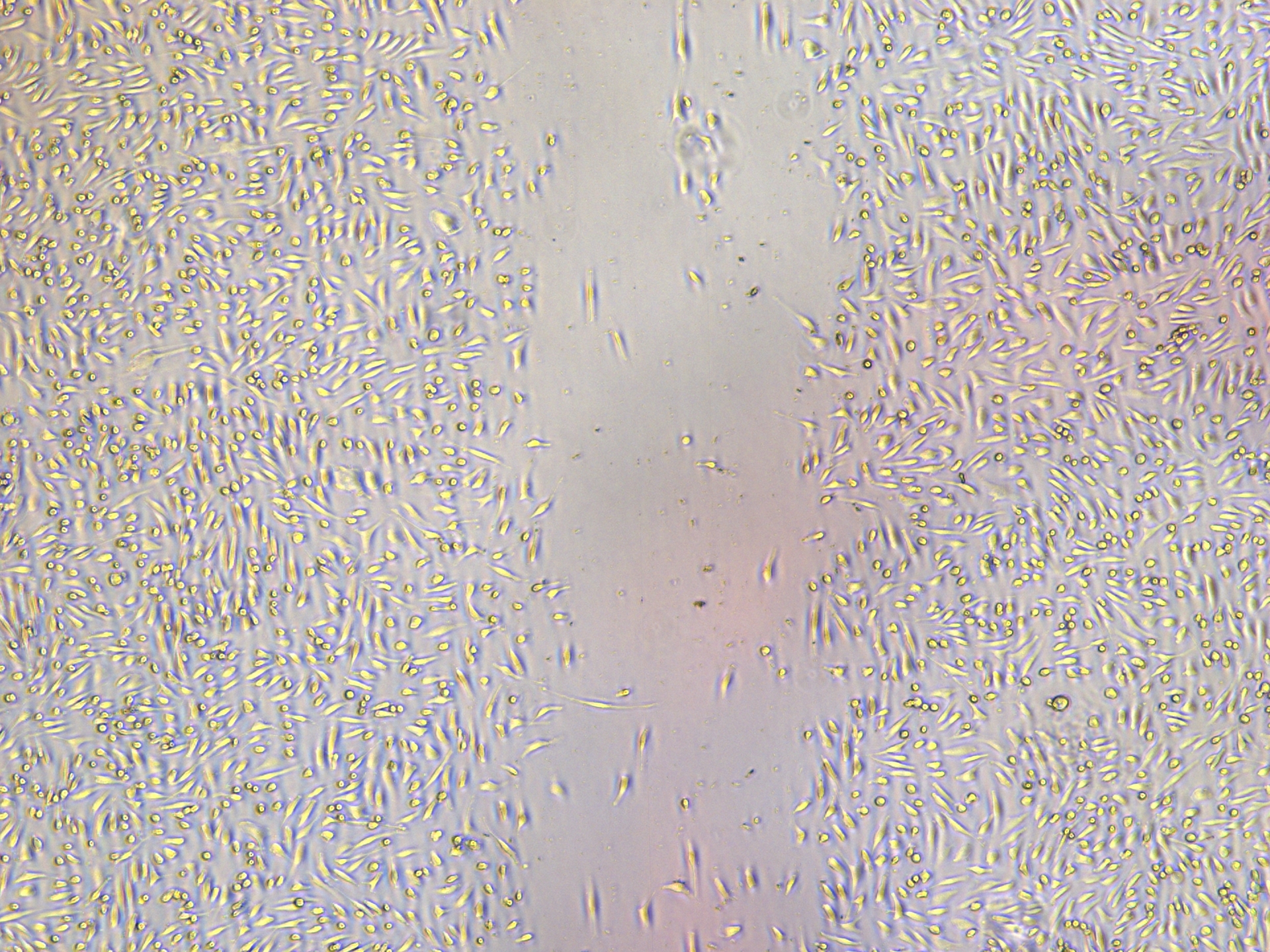

Supplement: Multimedia component 4 [file mmc4.zip › Original Figures/Figure 2. Microscopy images/Figure 2E. Scratch test - OE2110 - 24h.jpg]

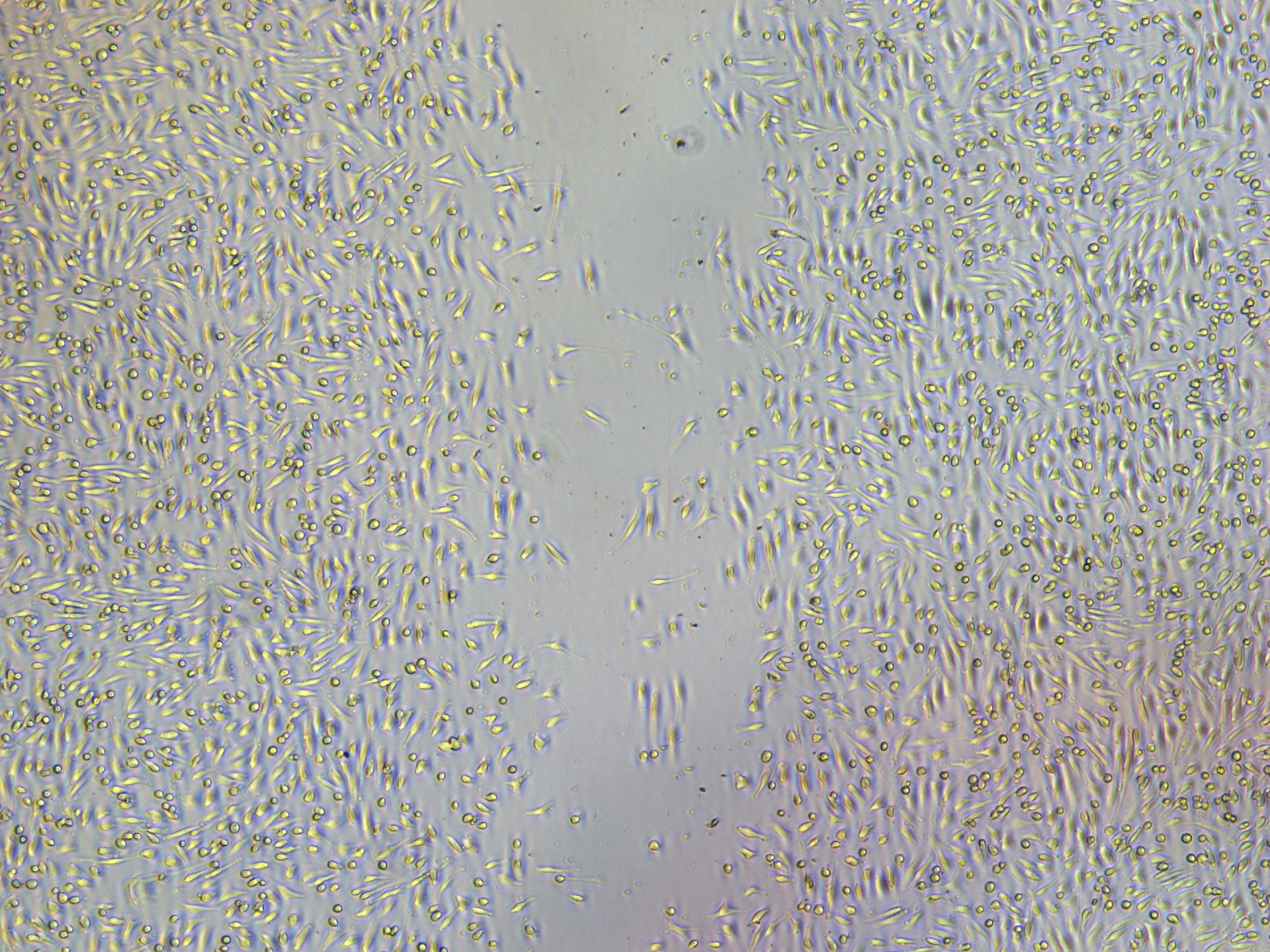

Supplement: Multimedia component 4 [file mmc4.zip › Original Figures/Figure 2. Microscopy images/Figure 2E. Scratch test - OE2110 - 36h.jpg]

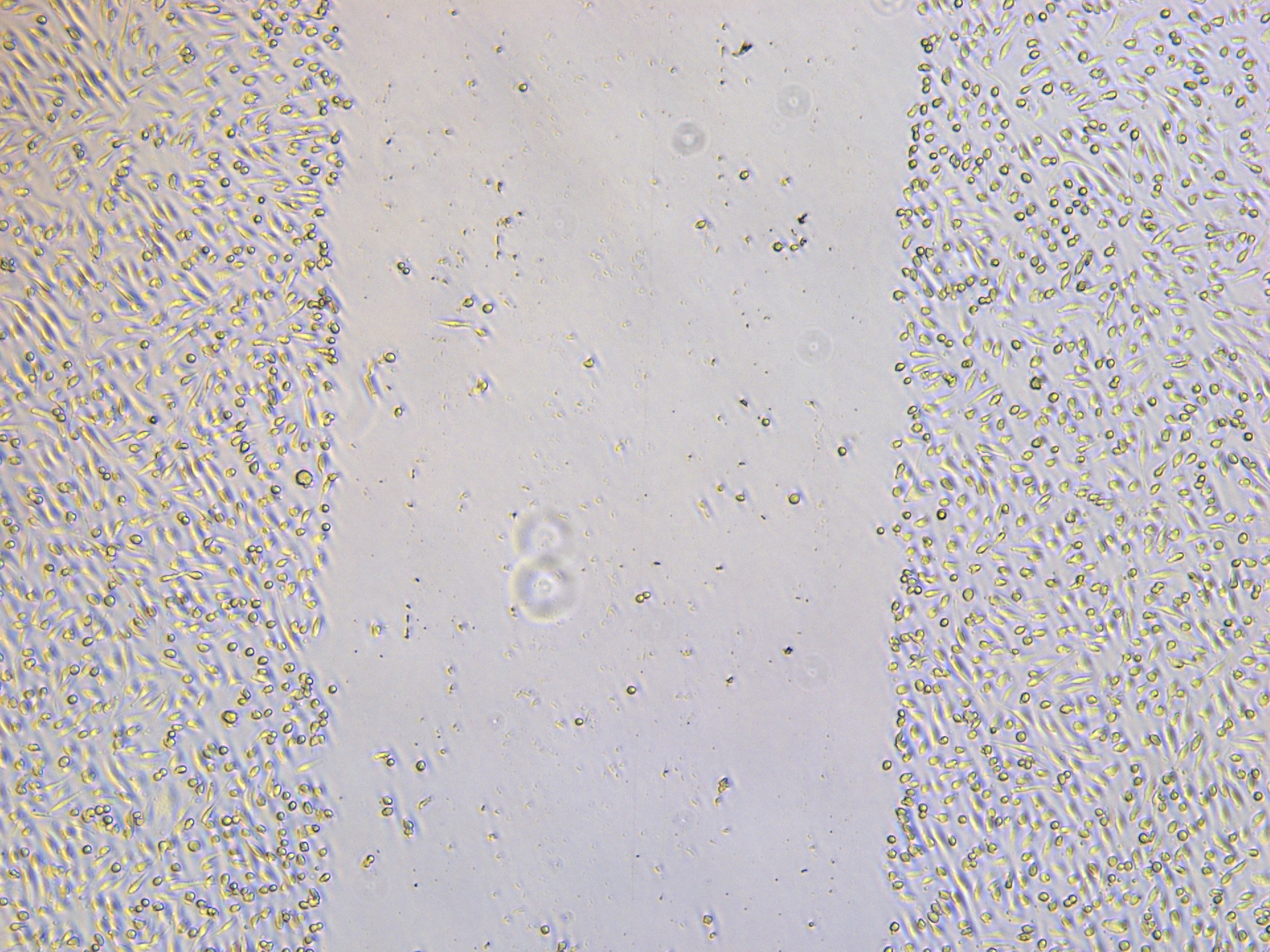

Supplement: Multimedia component 4 [file mmc4.zip › Original Figures/Figure 2. Microscopy images/Figure 2E. Scratch test - OENC - 0h.jpg]

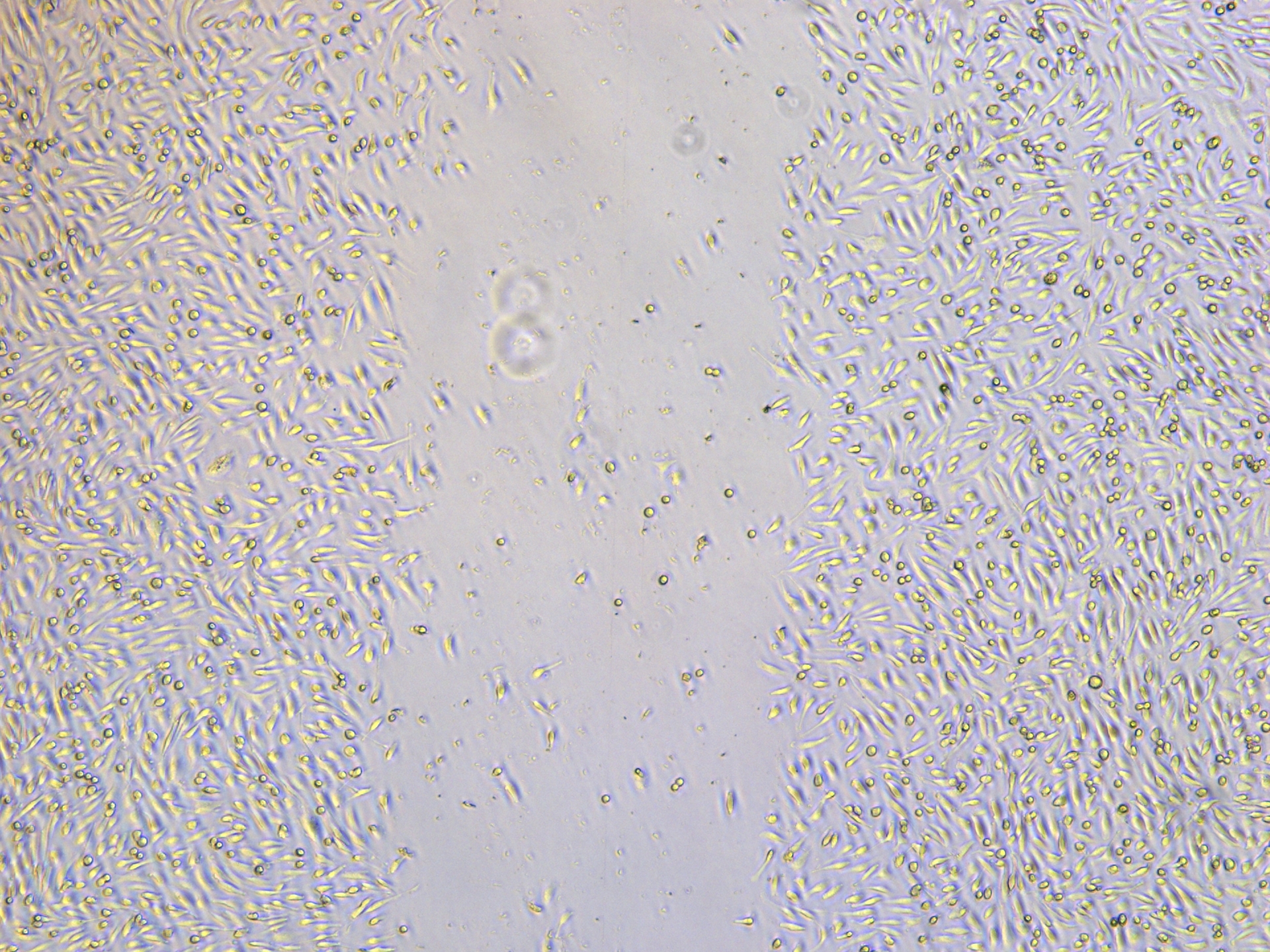

Supplement: Multimedia component 4 [file mmc4.zip › Original Figures/Figure 2. Microscopy images/Figure 2E. Scratch test - OENC - 12h.jpg]

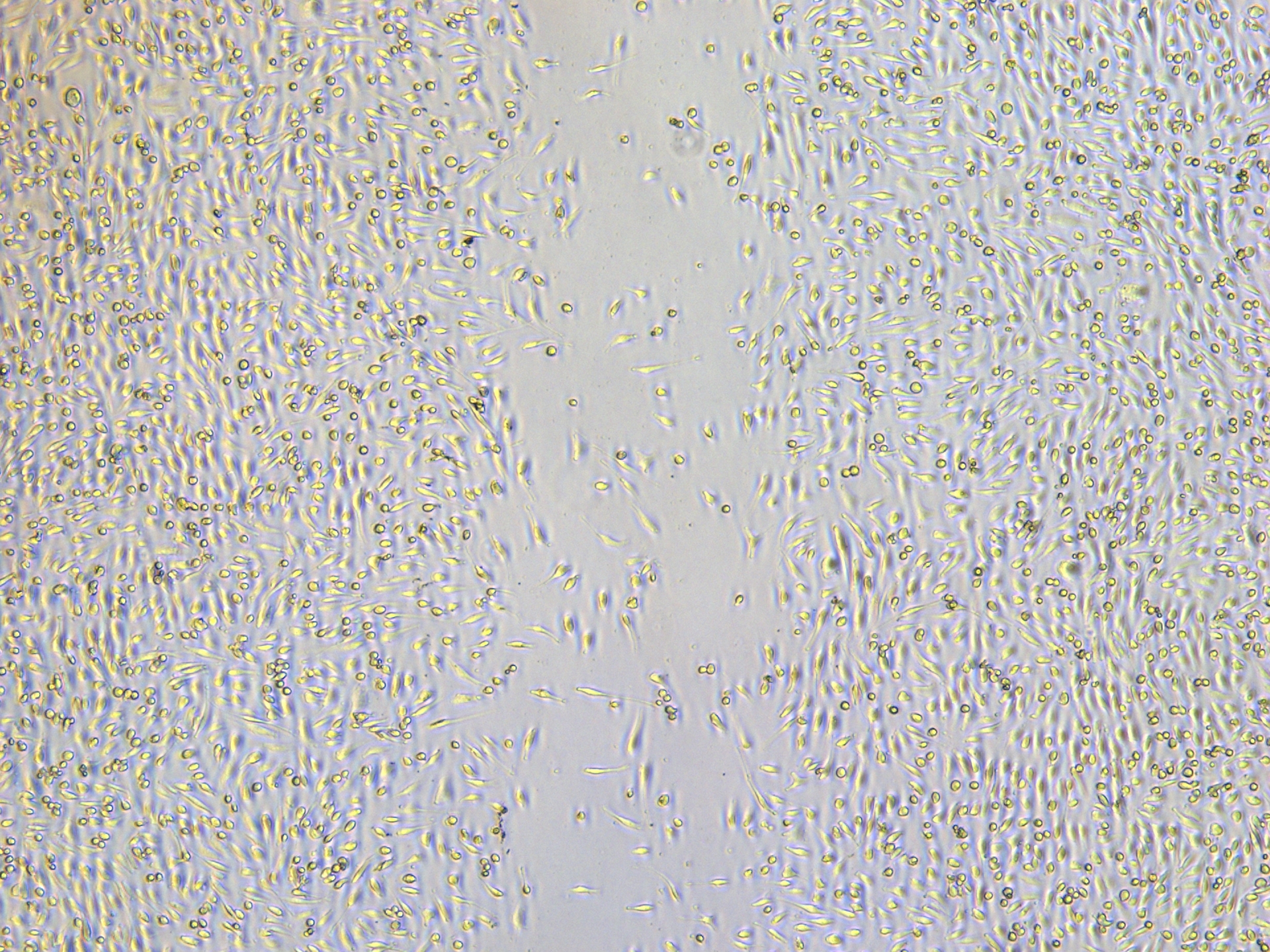

Supplement: Multimedia component 4 [file mmc4.zip › Original Figures/Figure 2. Microscopy images/Figure 2E. Scratch test - OENC - 24h.jpg]

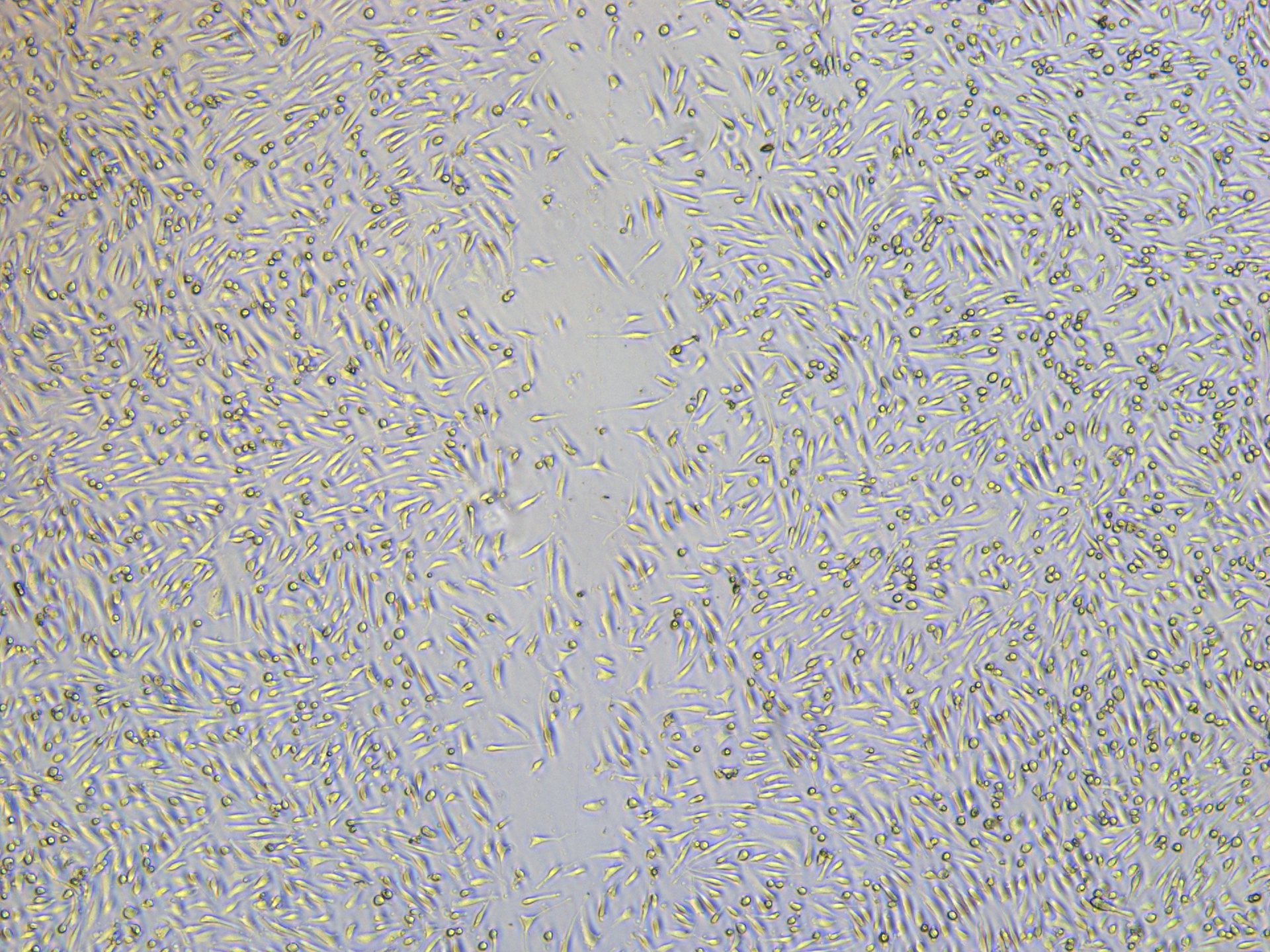

Supplement: Multimedia component 4 [file mmc4.zip › Original Figures/Figure 2. Microscopy images/Figure 2E. Scratch test - OENC - 36h.jpg]

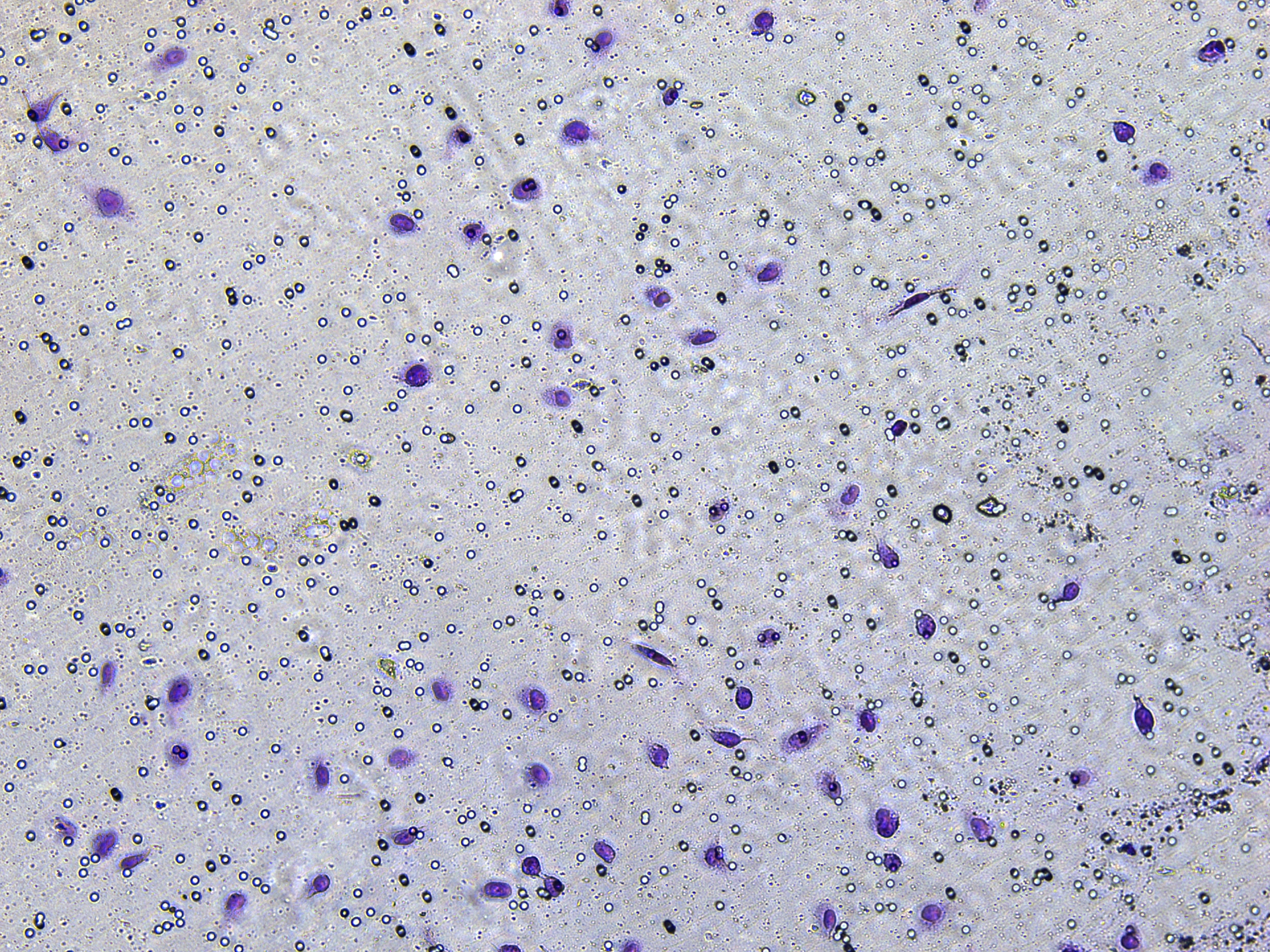

Supplement: Multimedia component 4 [file mmc4.zip › Original Figures/Figure 2. Microscopy images/Figure 2G. Transwell - OE2110.tif]

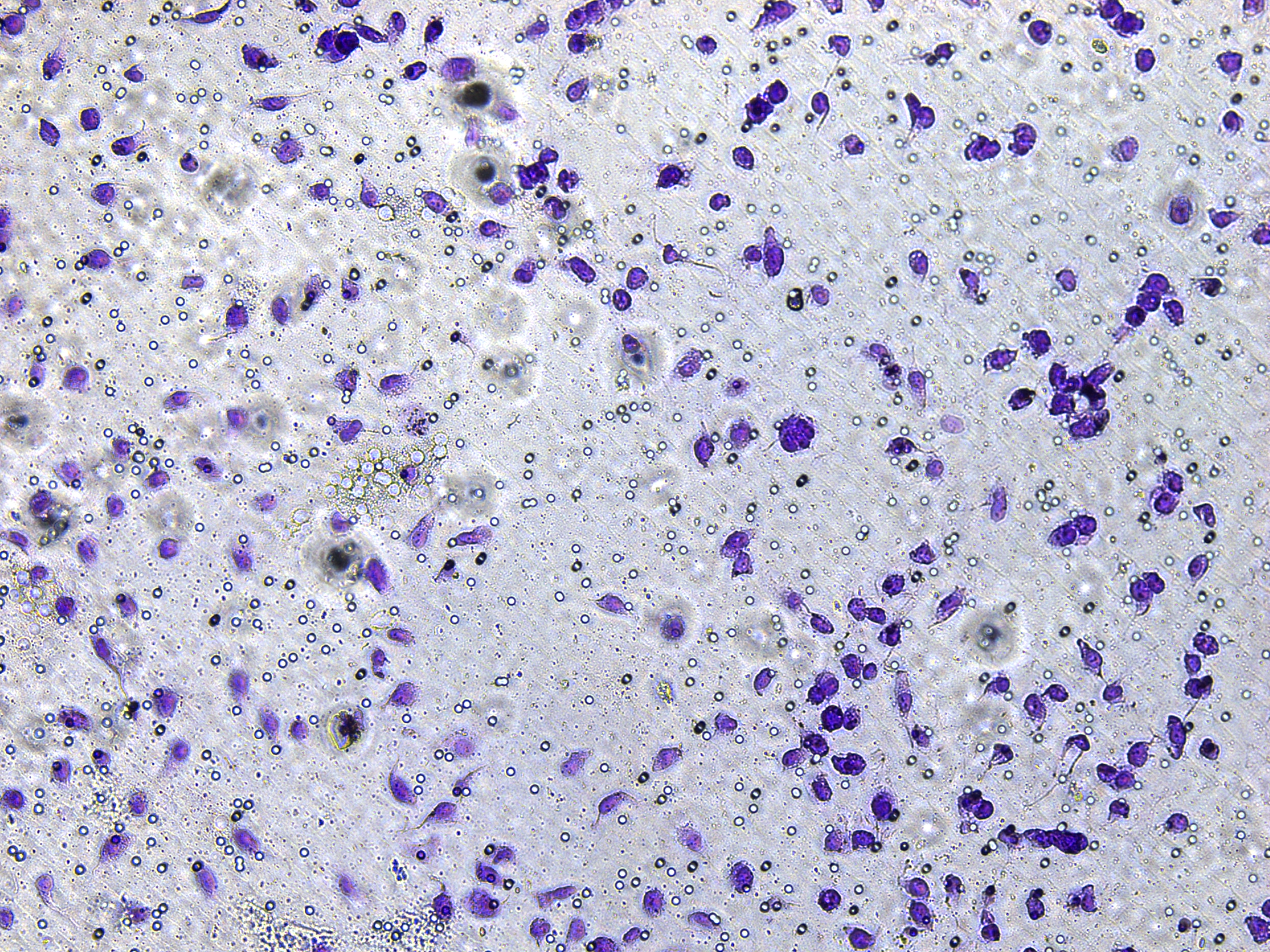

Supplement: Multimedia component 4 [file mmc4.zip › Original Figures/Figure 2. Microscopy images/Figure 2G. Transwell - OENC.tif]

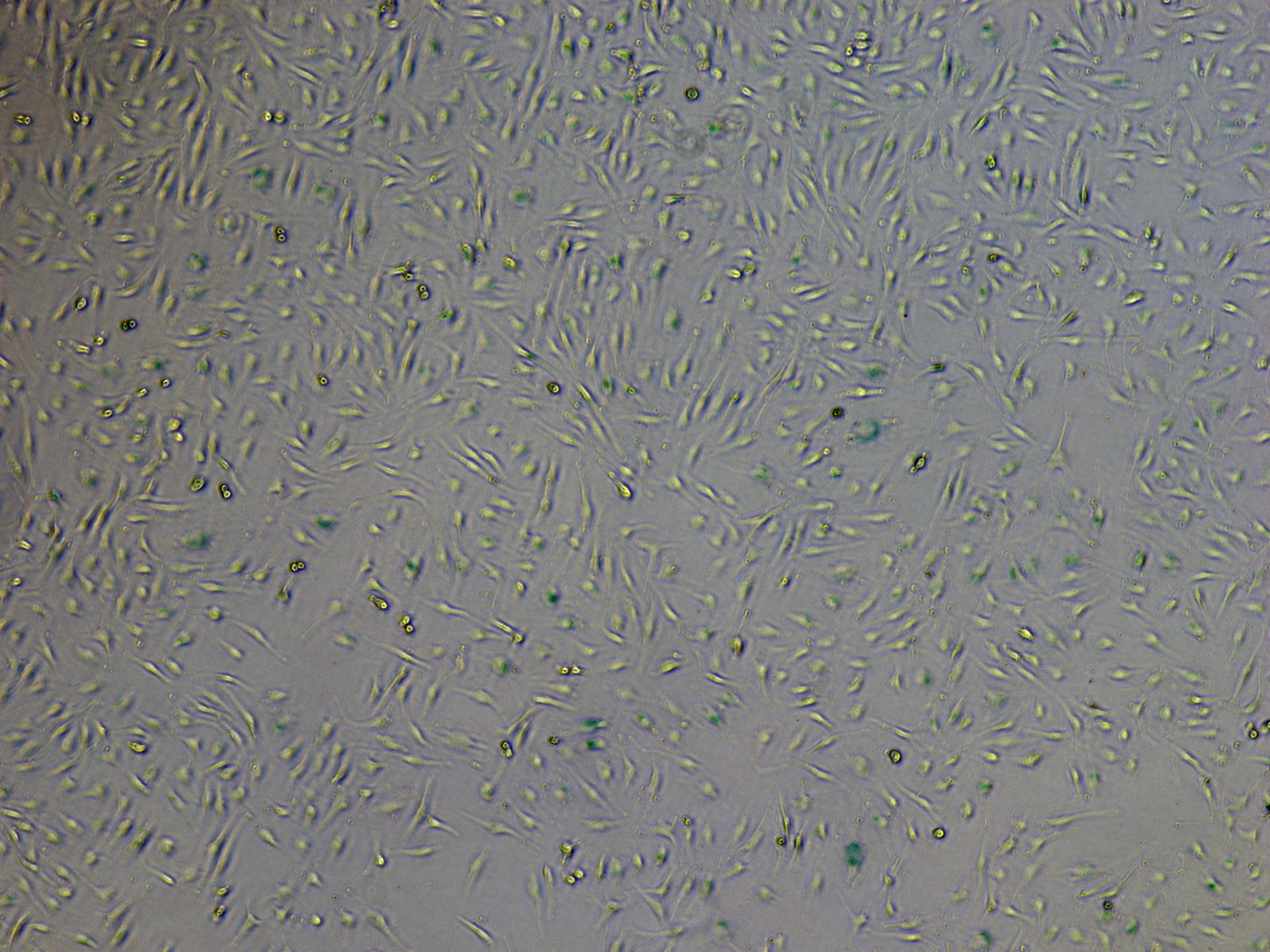

Supplement: Multimedia component 4 [file mmc4.zip › Original Figures/Figure 3. Microscopy images/Figure 3E. B-Galactosidease test - OE2110.tif]

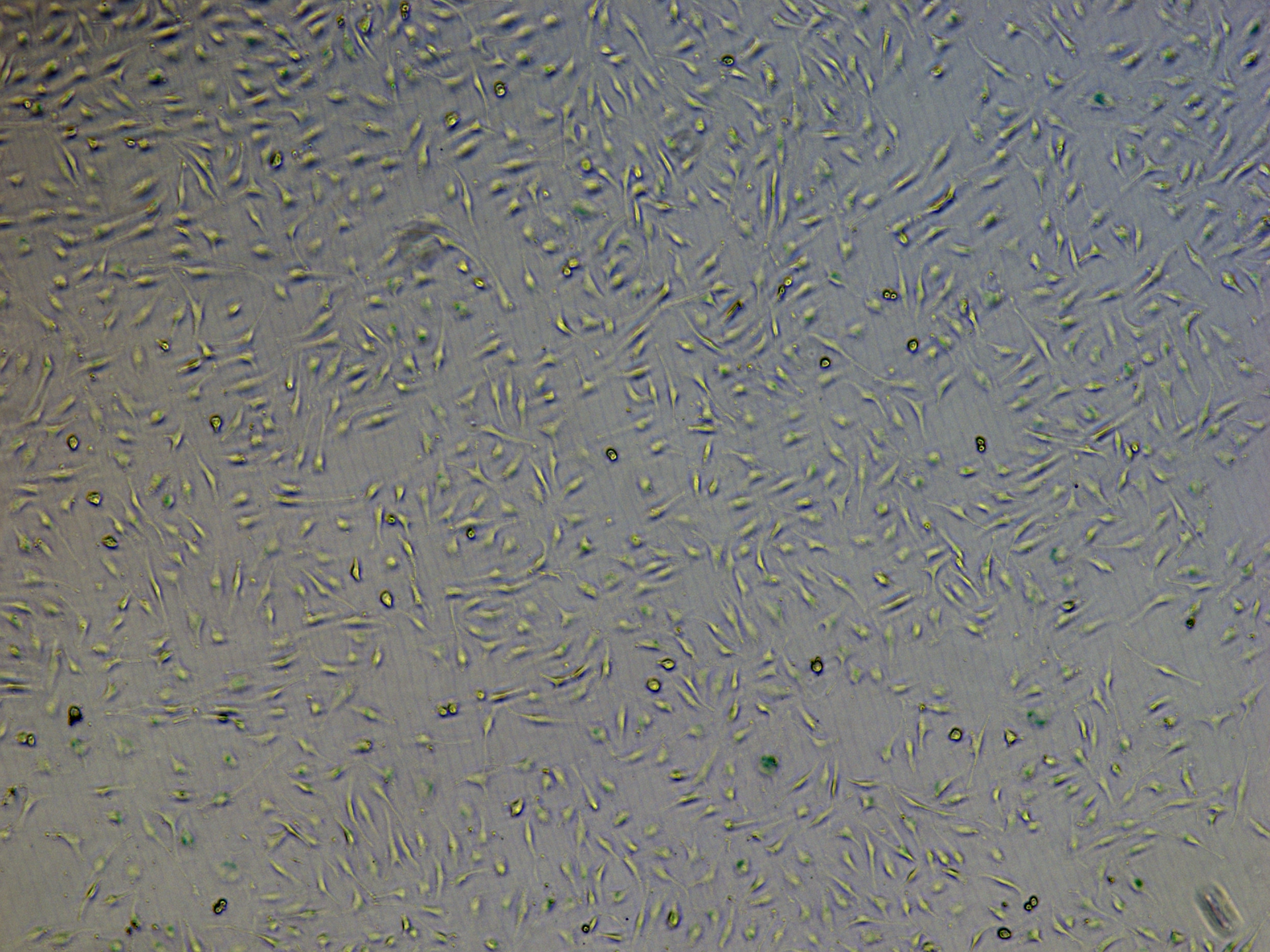

Supplement: Multimedia component 4 [file mmc4.zip › Original Figures/Figure 3. Microscopy images/Figure 3E. B-Galactosidease test - OENC.tif]

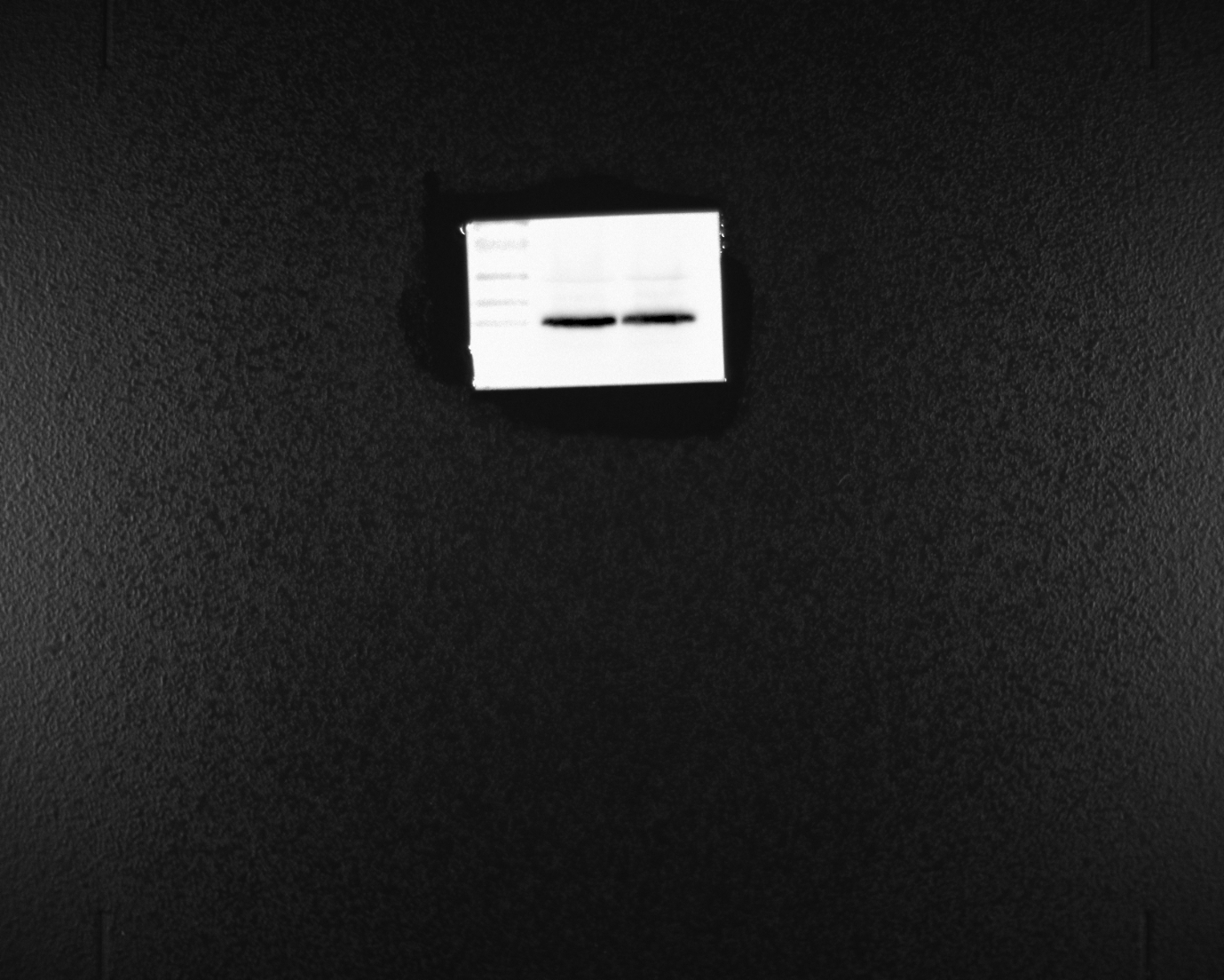

Supplement: Multimedia component 4 [file mmc4.zip › Original Figures/Figure 5. WB original images/GAPDH - OE2110 vs OENC.tif]

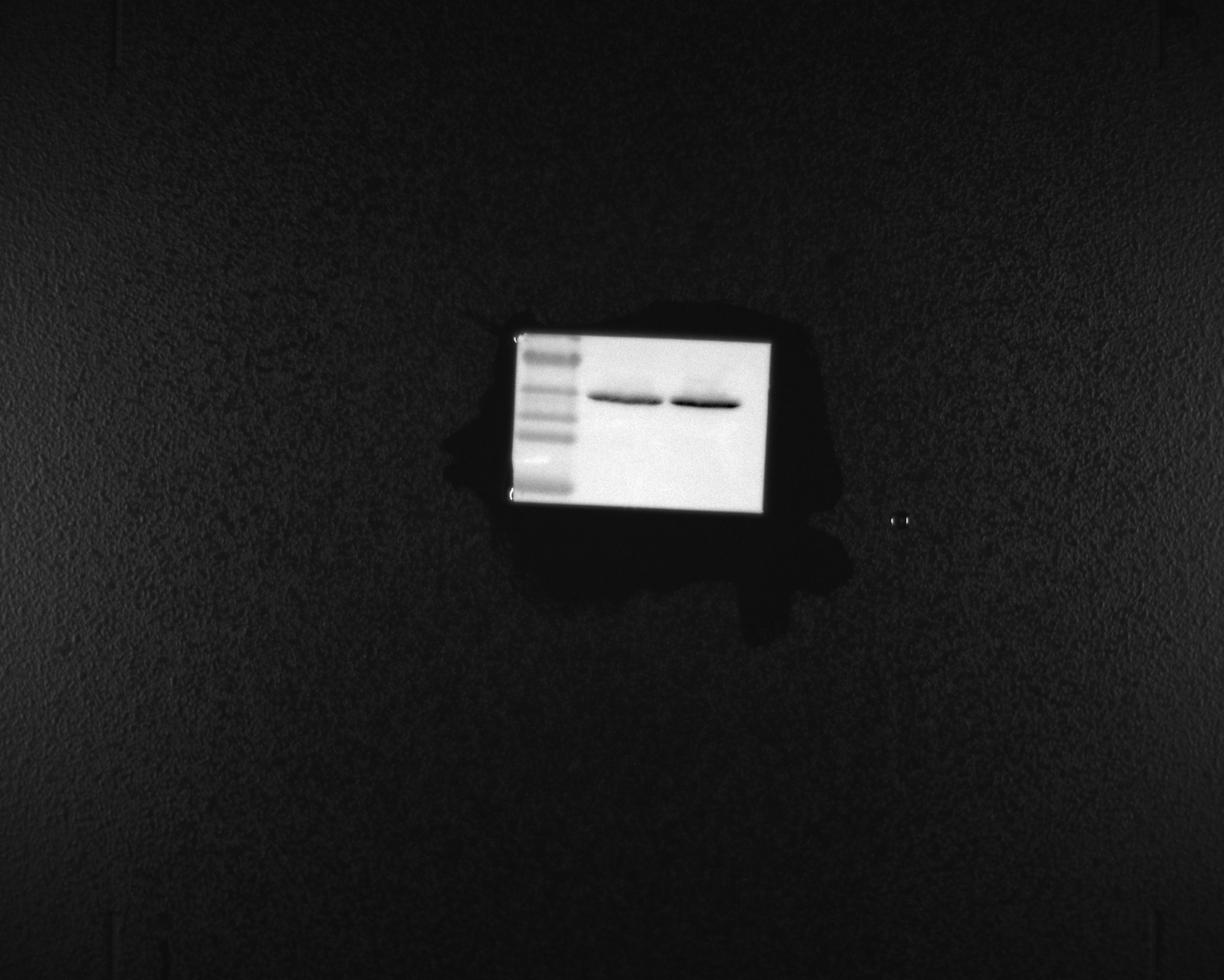

Supplement: Multimedia component 4 [file mmc4.zip › Original Figures/Figure 5. WB original images/TRAF3 - OE2110 vs OENC.tif]

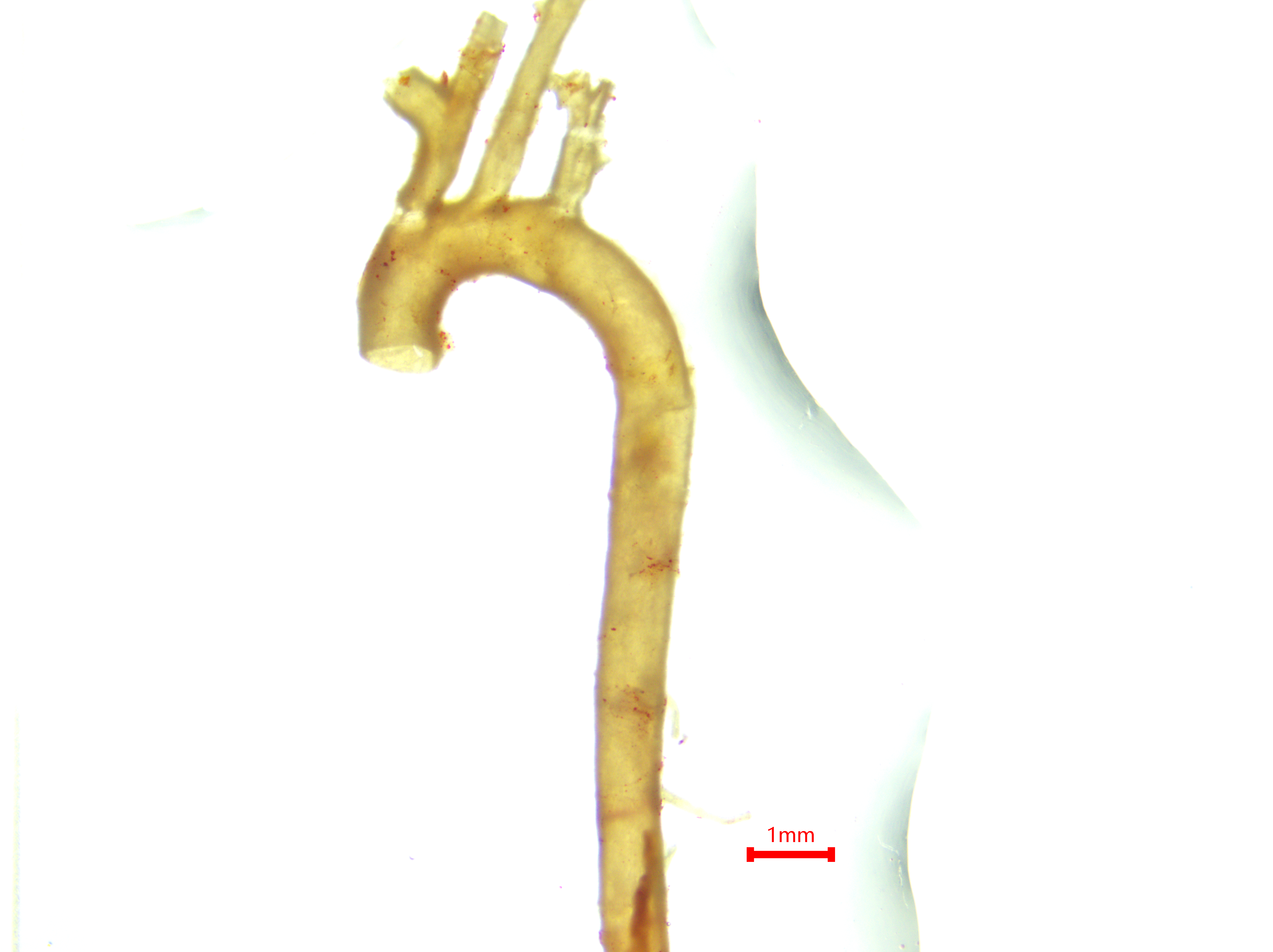

Supplement: Multimedia component 4 [file mmc4.zip › Original Figures/Figure 6. Microscopy images/Figure 6A. Part 1.tif]

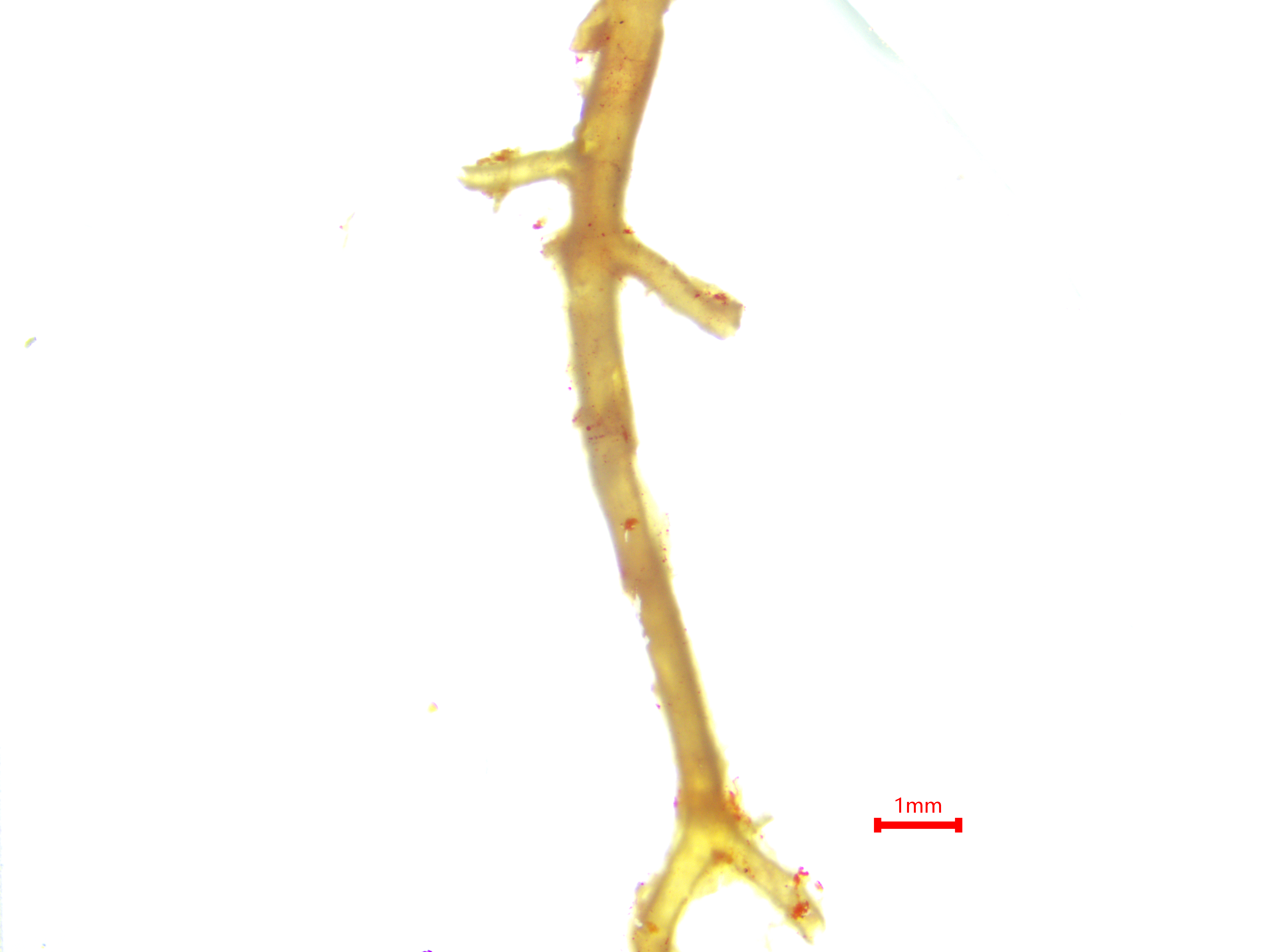

Supplement: Multimedia component 4 [file mmc4.zip › Original Figures/Figure 6. Microscopy images/Figure 6A. Part 2.tif]

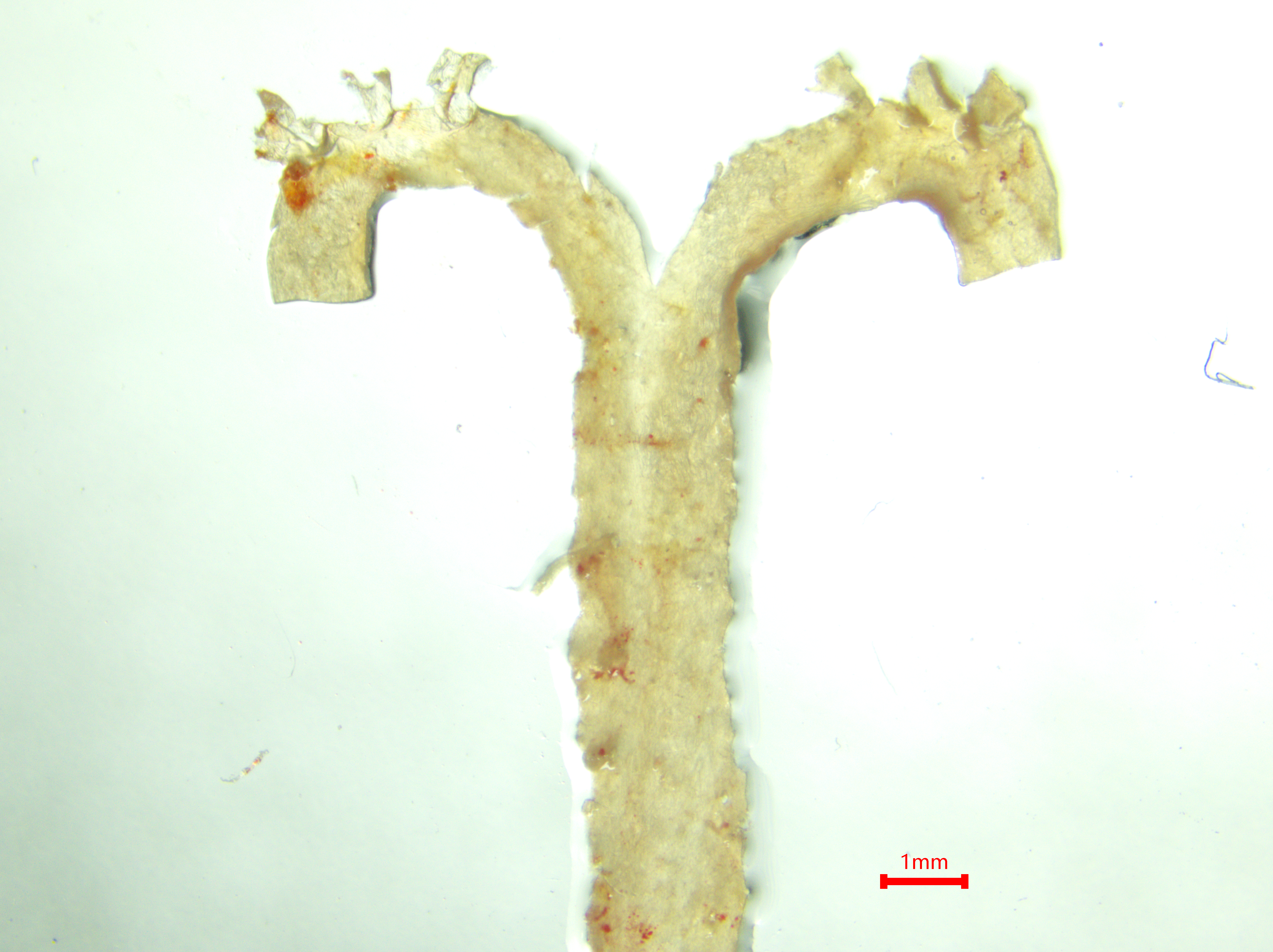

Supplement: Multimedia component 4 [file mmc4.zip › Original Figures/Figure 6. Microscopy images/Figure 6B.tif]

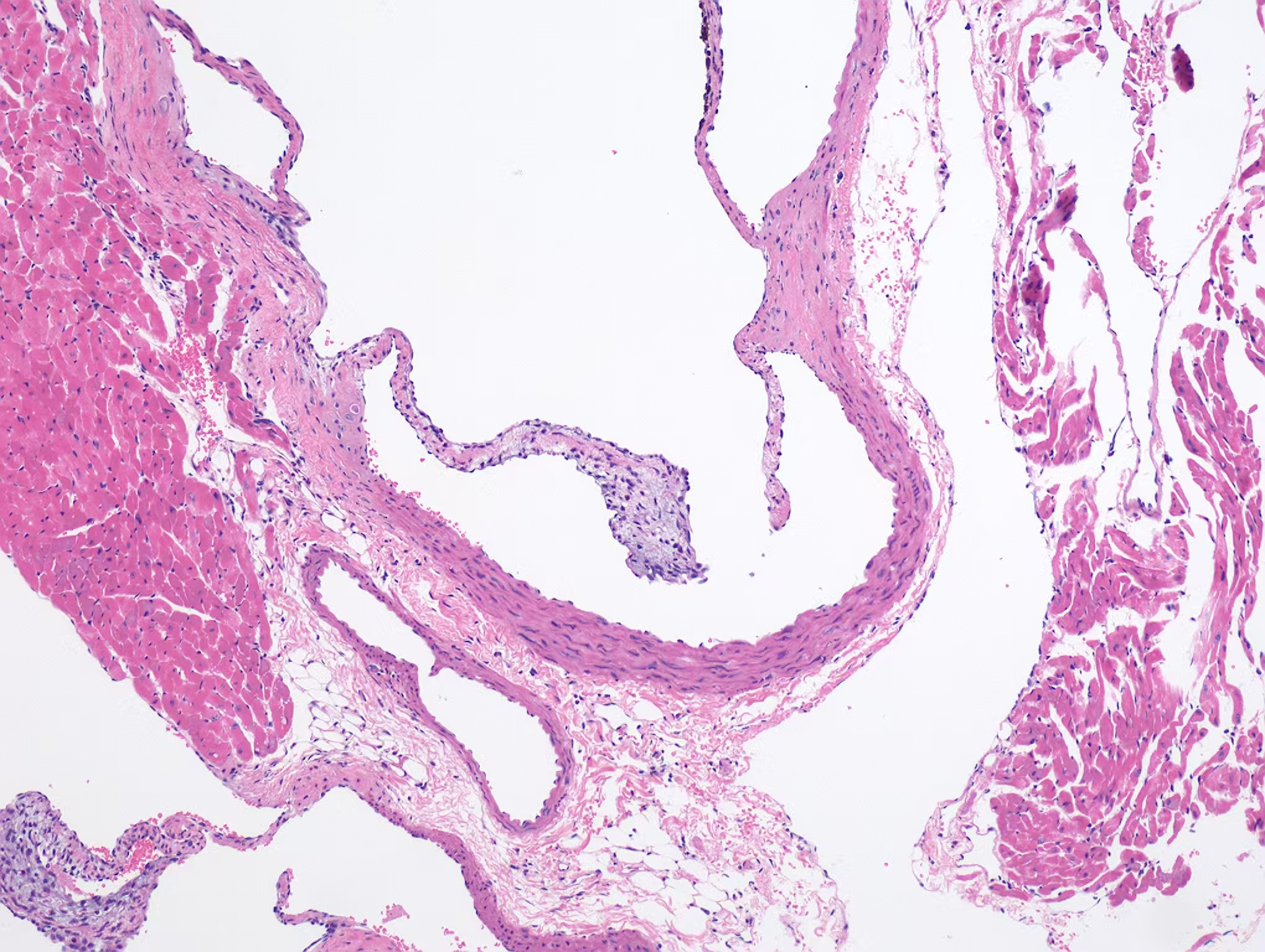

Supplement: Multimedia component 4 [file mmc4.zip › Original Figures/Figure 6. Microscopy images/Figure 6C.jpg]

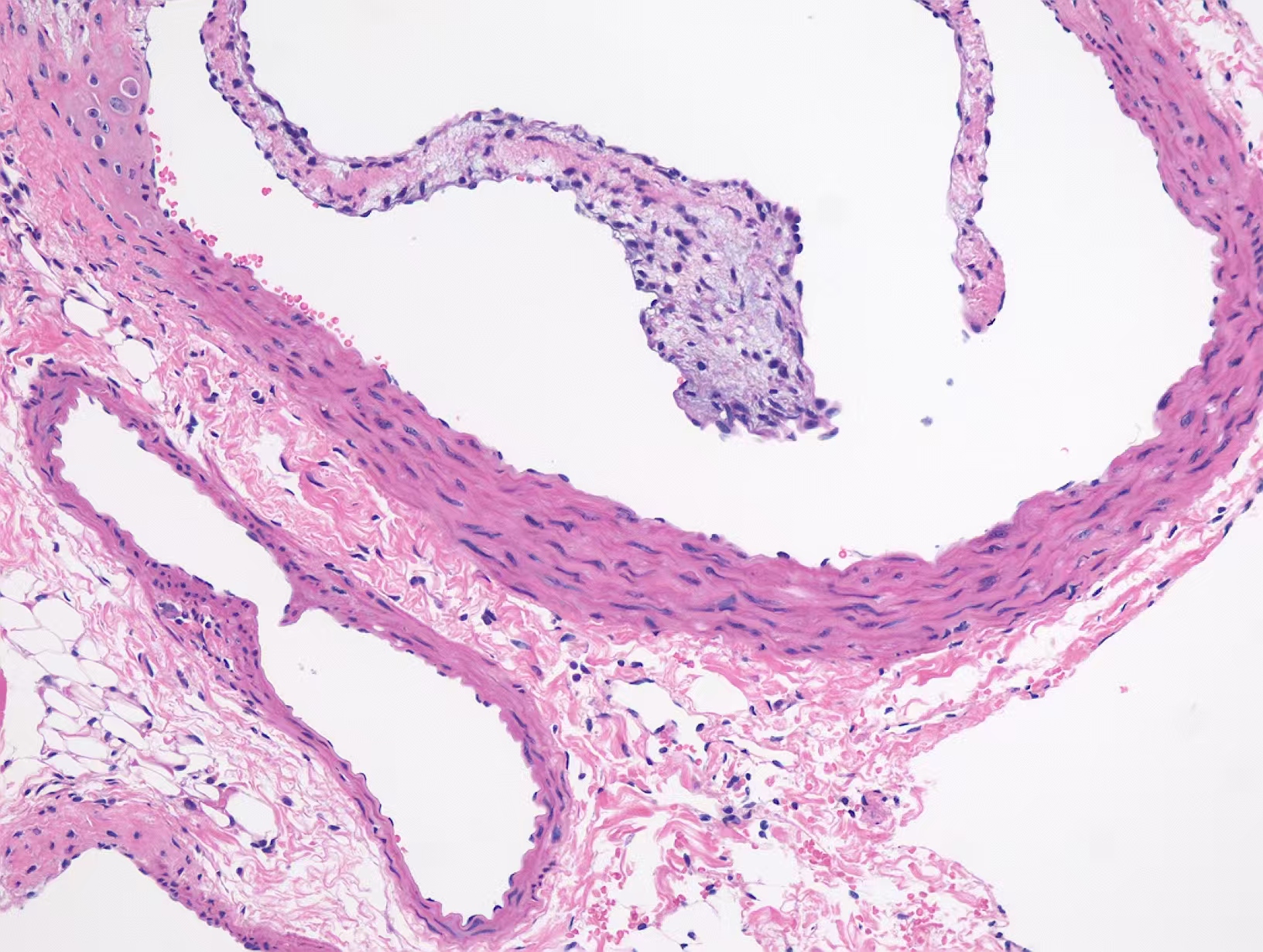

Supplement: Multimedia component 4 [file mmc4.zip › Original Figures/Figure 6. Microscopy images/Figure 6D.jpg]

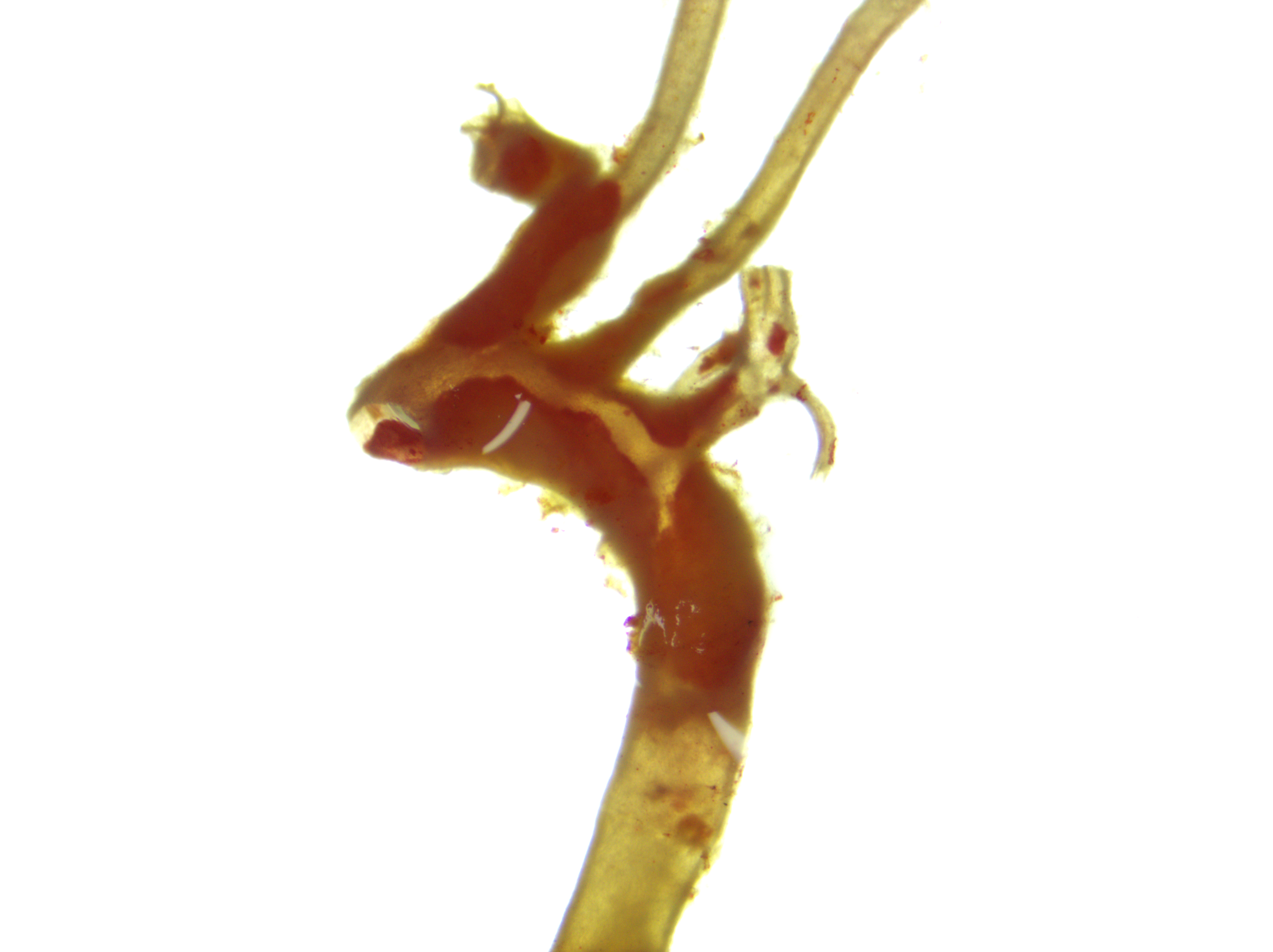

Supplement: Multimedia component 4 [file mmc4.zip › Original Figures/Figure 6. Microscopy images/Figure 6E. Part 1.tif]

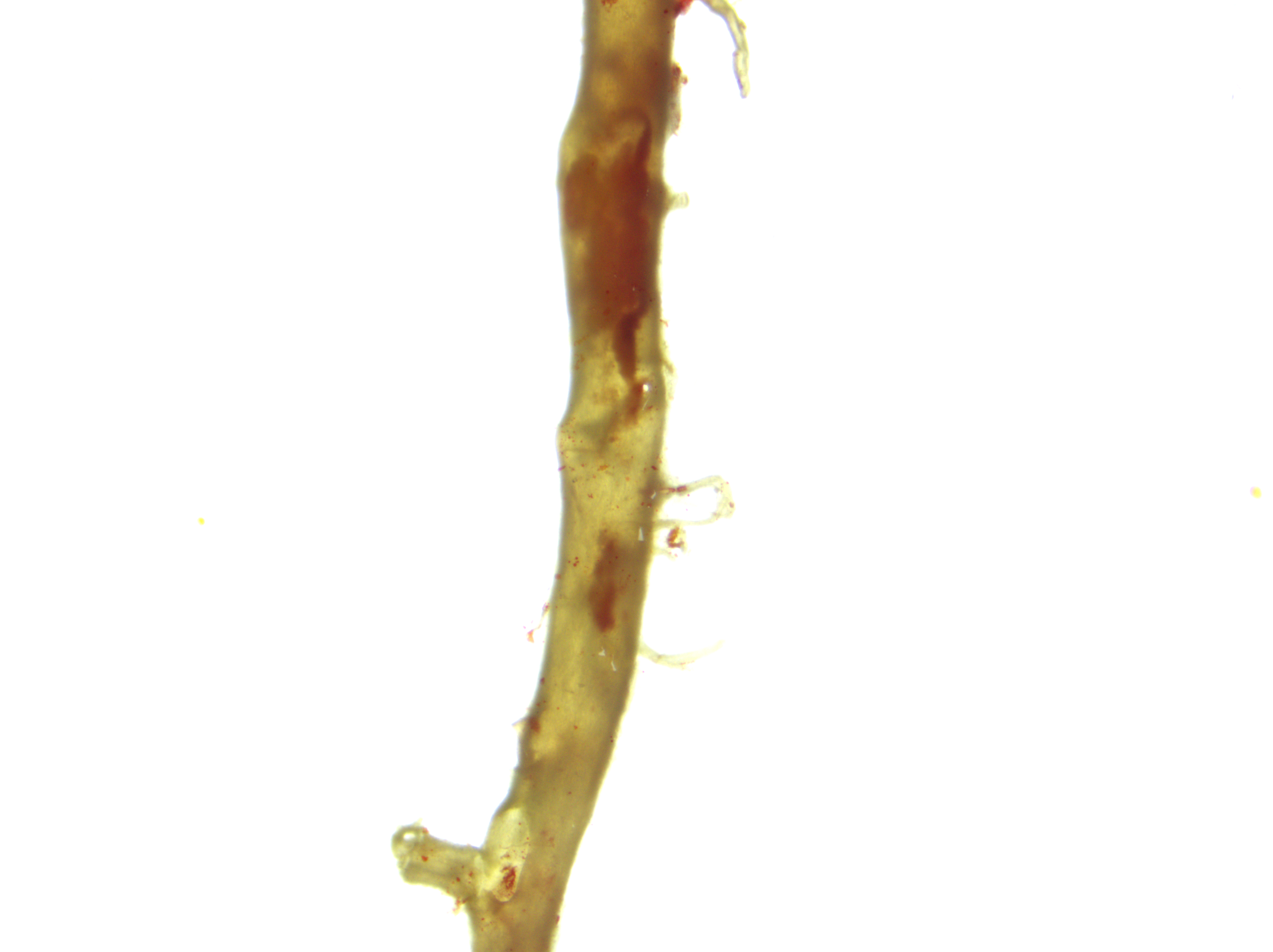

Supplement: Multimedia component 4 [file mmc4.zip › Original Figures/Figure 6. Microscopy images/Figure 6E. Part 2.tif]

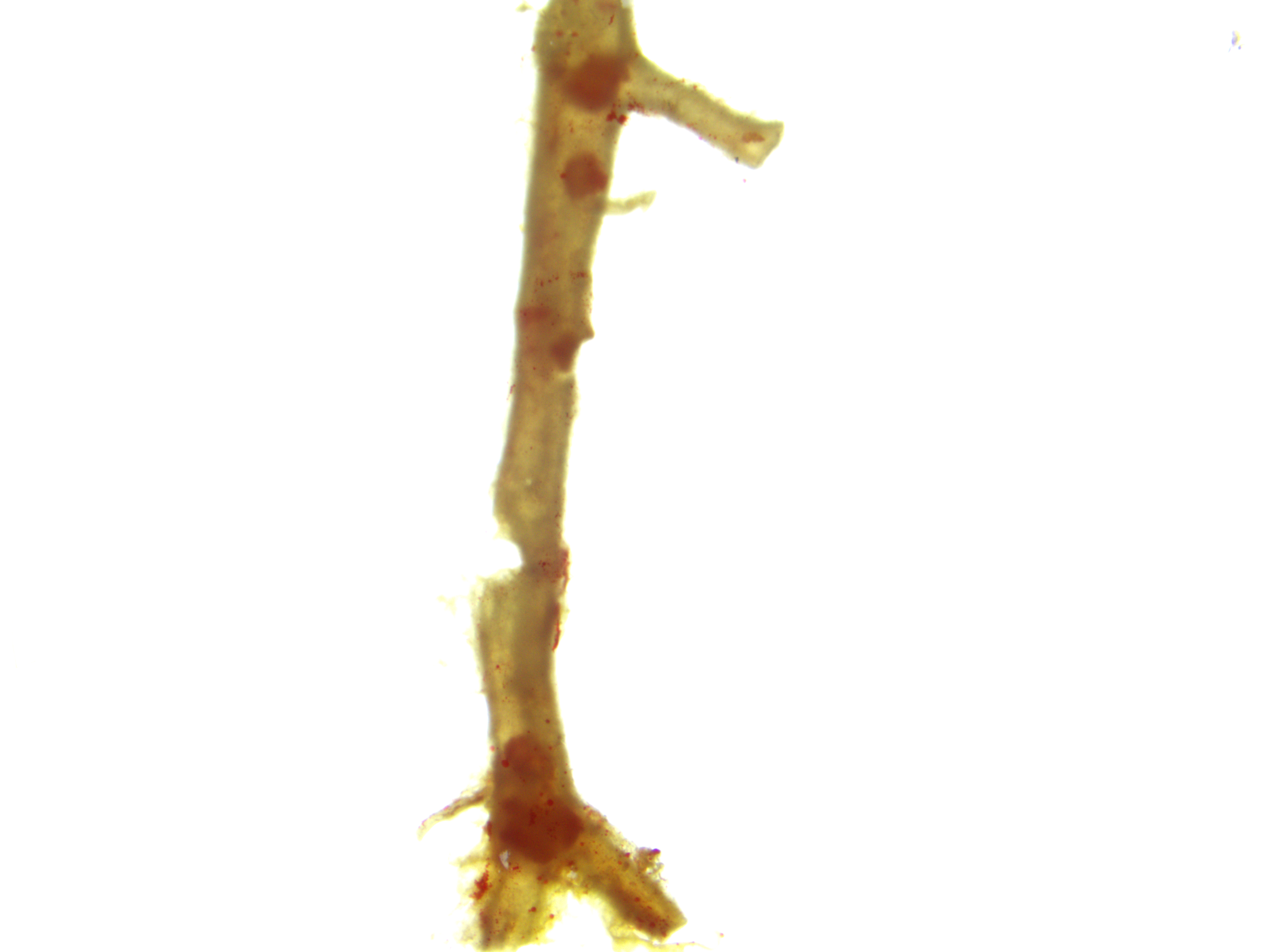

Supplement: Multimedia component 4 [file mmc4.zip › Original Figures/Figure 6. Microscopy images/Figure 6E. Part 3.tif]

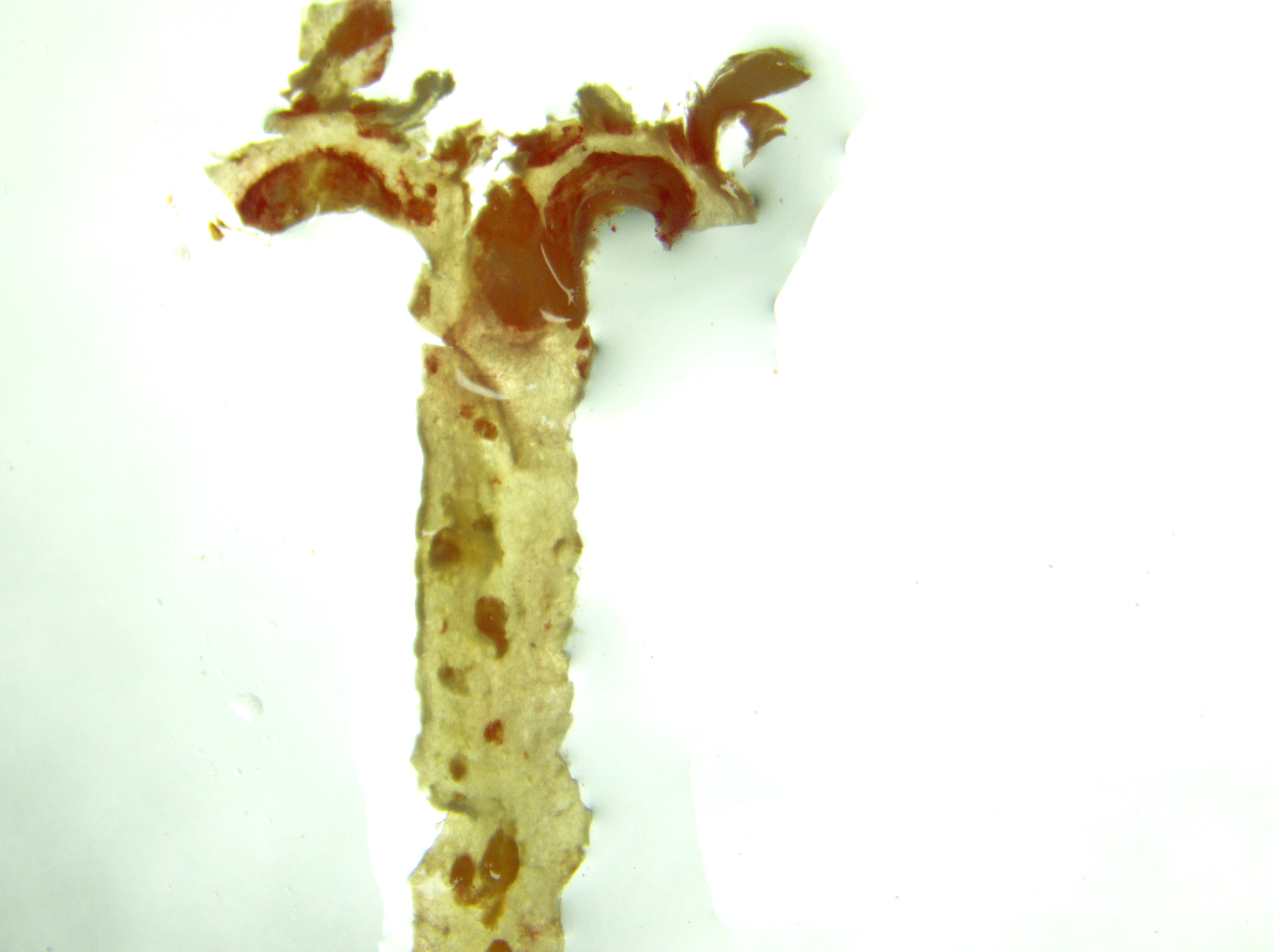

Supplement: Multimedia component 4 [file mmc4.zip › Original Figures/Figure 6. Microscopy images/Figure 6F.tif]

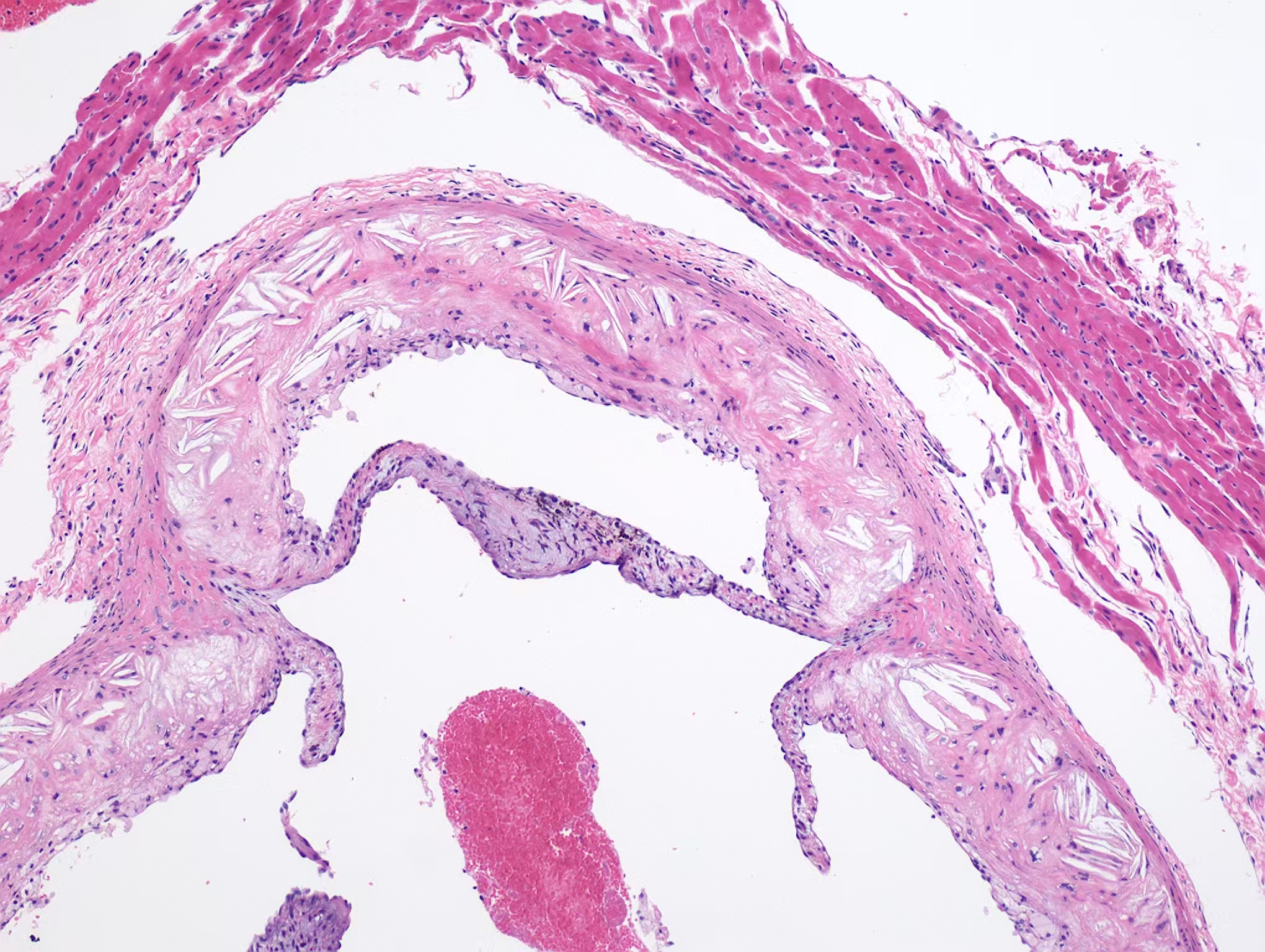

Supplement: Multimedia component 4 [file mmc4.zip › Original Figures/Figure 6. Microscopy images/Figure 6G.jpg]

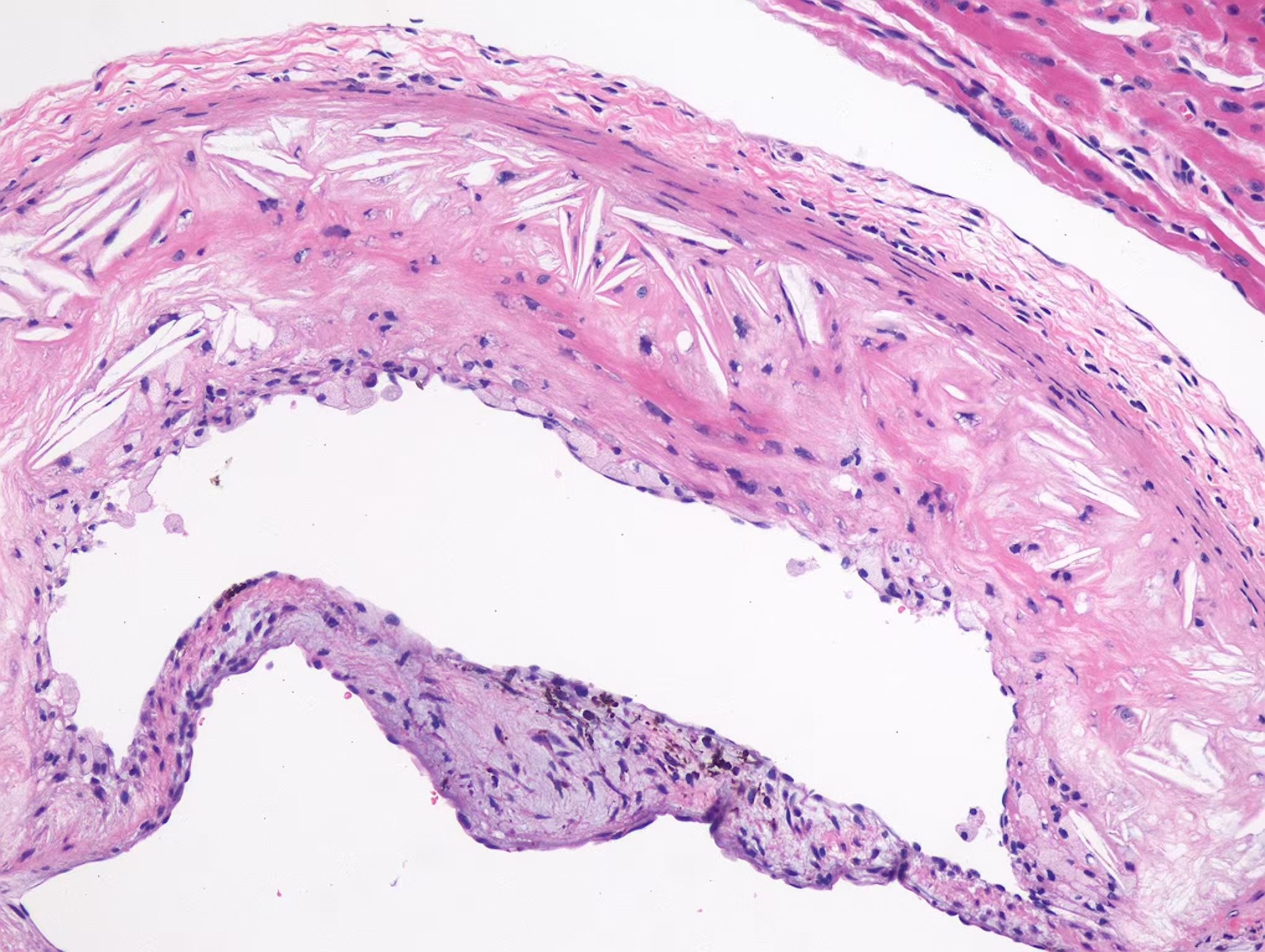

Supplement: Multimedia component 4 [file mmc4.zip › Original Figures/Figure 6. Microscopy images/Figure 6H.jpg]

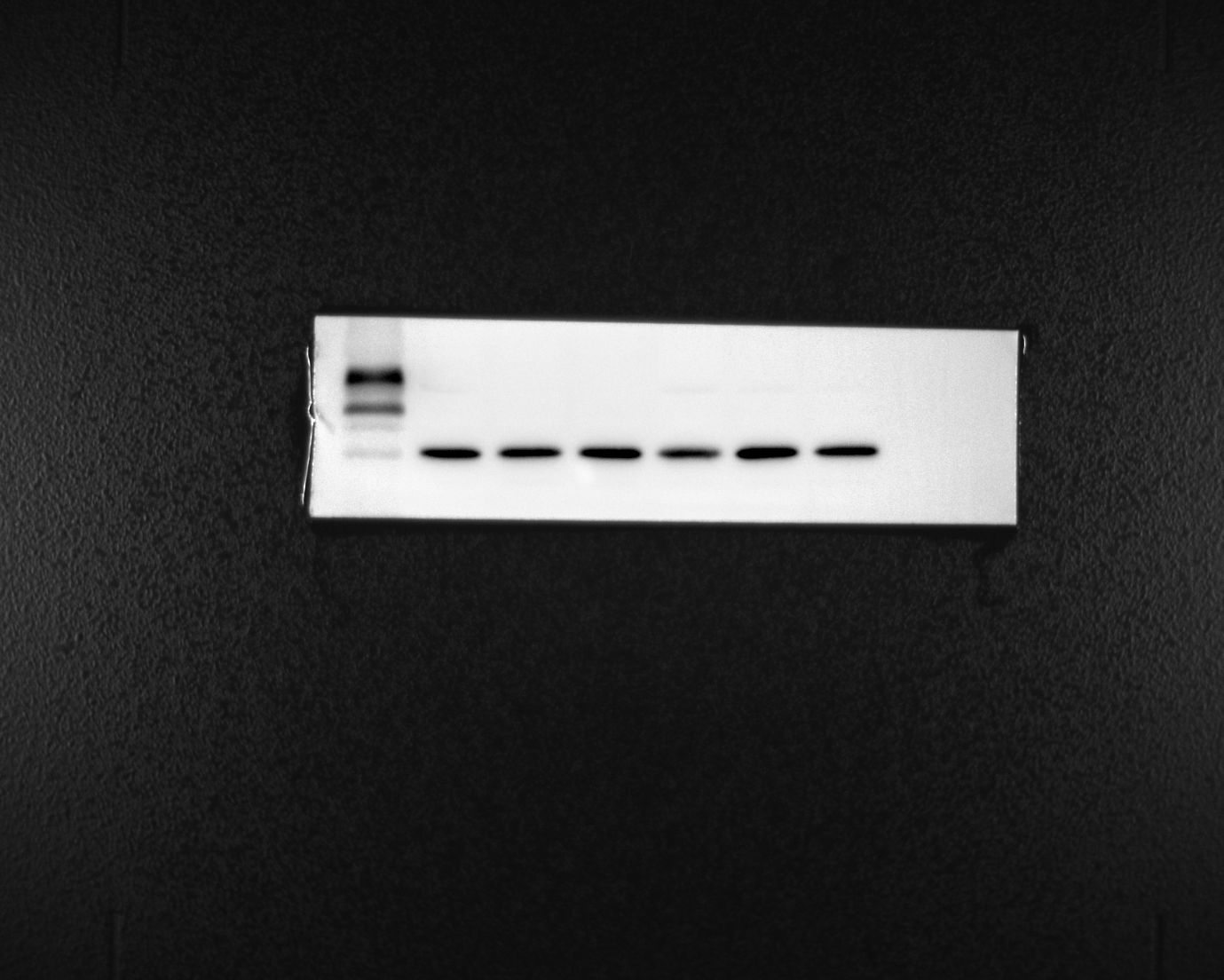

Supplement: Multimedia component 4 [file mmc4.zip › Original Figures/Figure 7. IHC and WB original images/Figure 7B. Heart - WB GAPDH (36kDa) - (3 controls vs 3 HFD).tif]

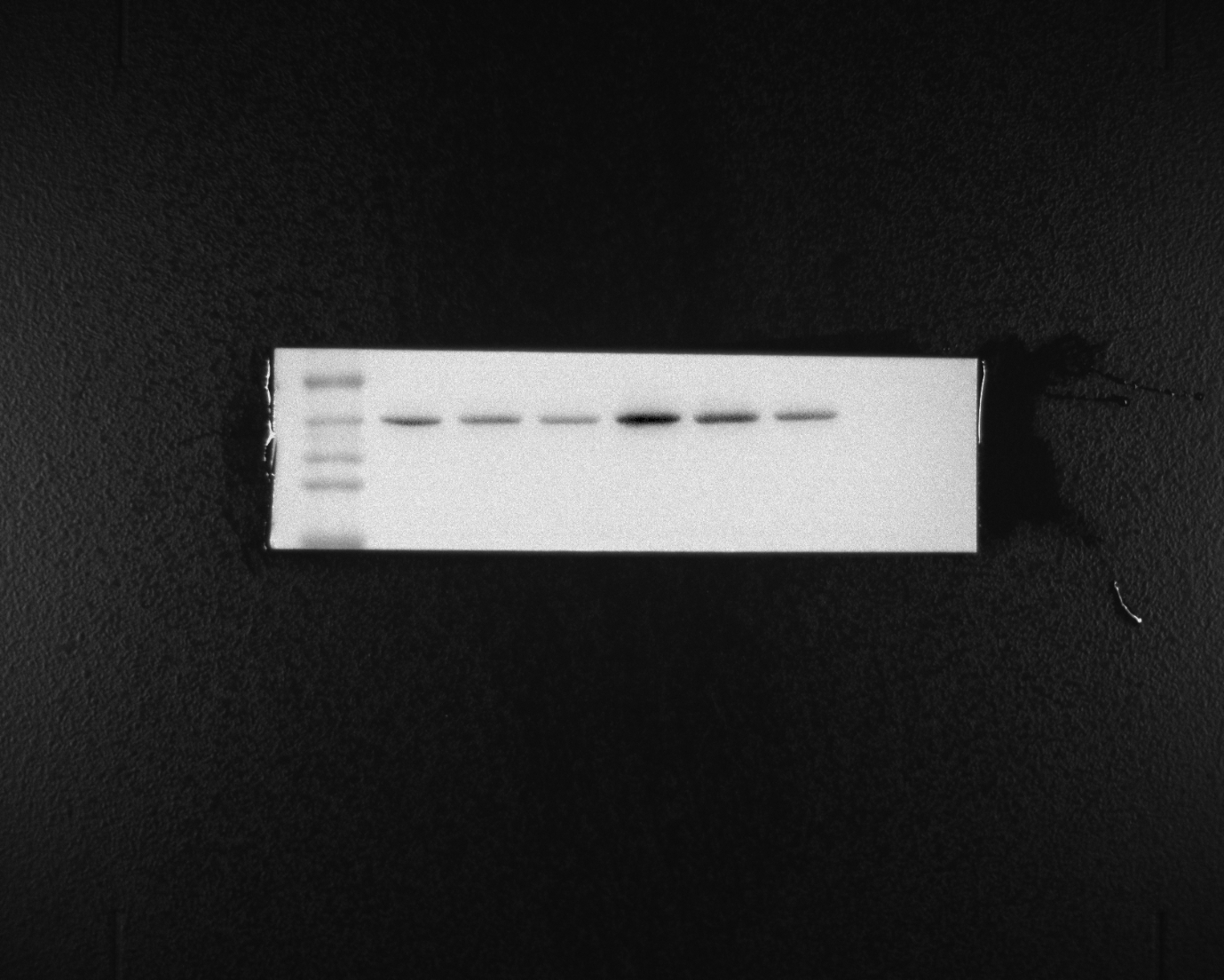

Supplement: Multimedia component 4 [file mmc4.zip › Original Figures/Figure 7. IHC and WB original images/Figure 7B. Heart - WB TRAF3 (64kDa) - (3 controls vs 3 HFD).tif]

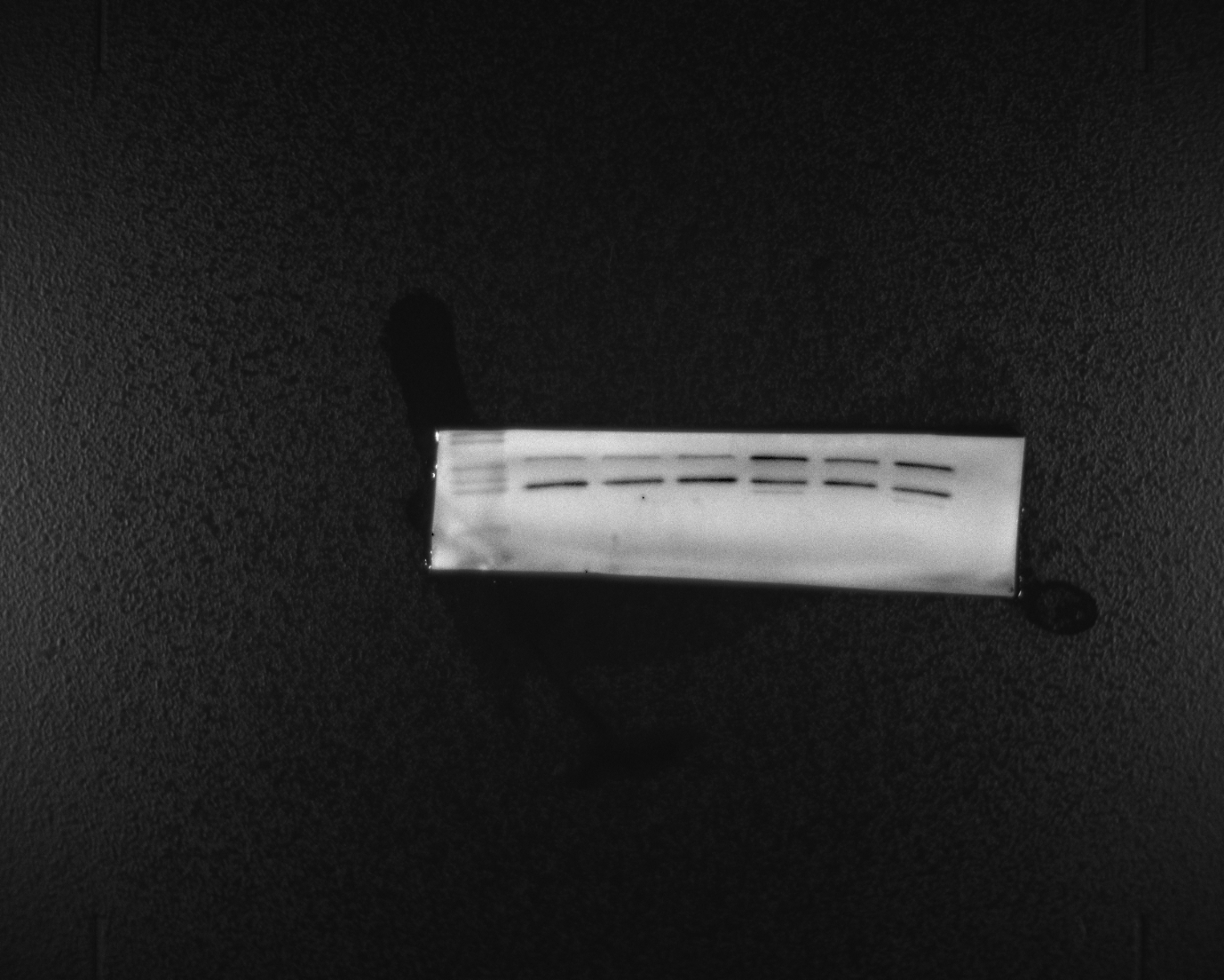

Supplement: Multimedia component 4 [file mmc4.zip › Original Figures/Figure 7. IHC and WB original images/Figure 7D. Aorta - WB TRAF3 (64kDa) and GAPDH (36kDa) - (3 controls vs 3 HFD).tif]

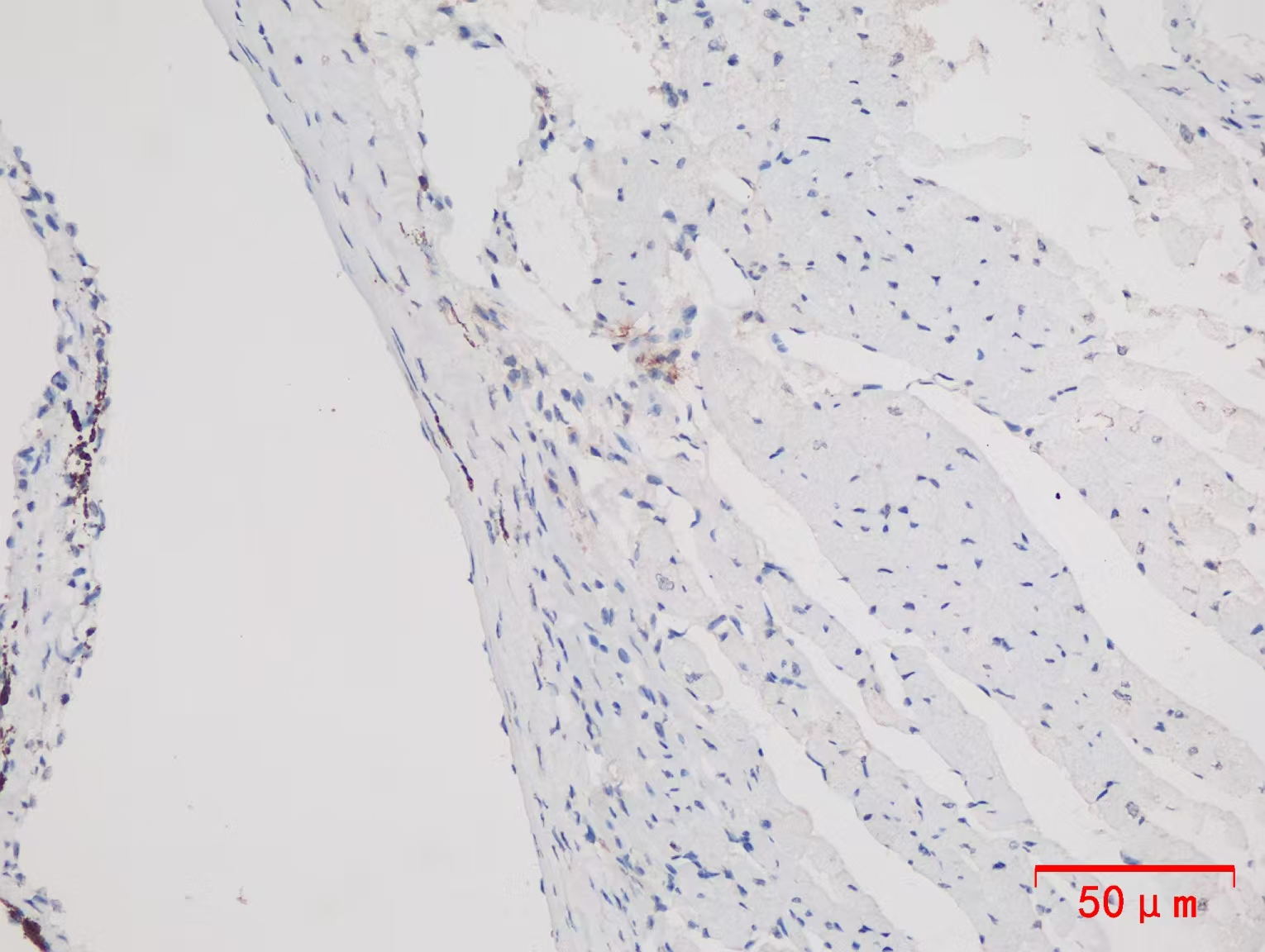

Supplement: Multimedia component 4 [file mmc4.zip › Original Figures/Figure 7. IHC and WB original images/Figure 7F. IHC/Figure 7F. Control 1.jpg]

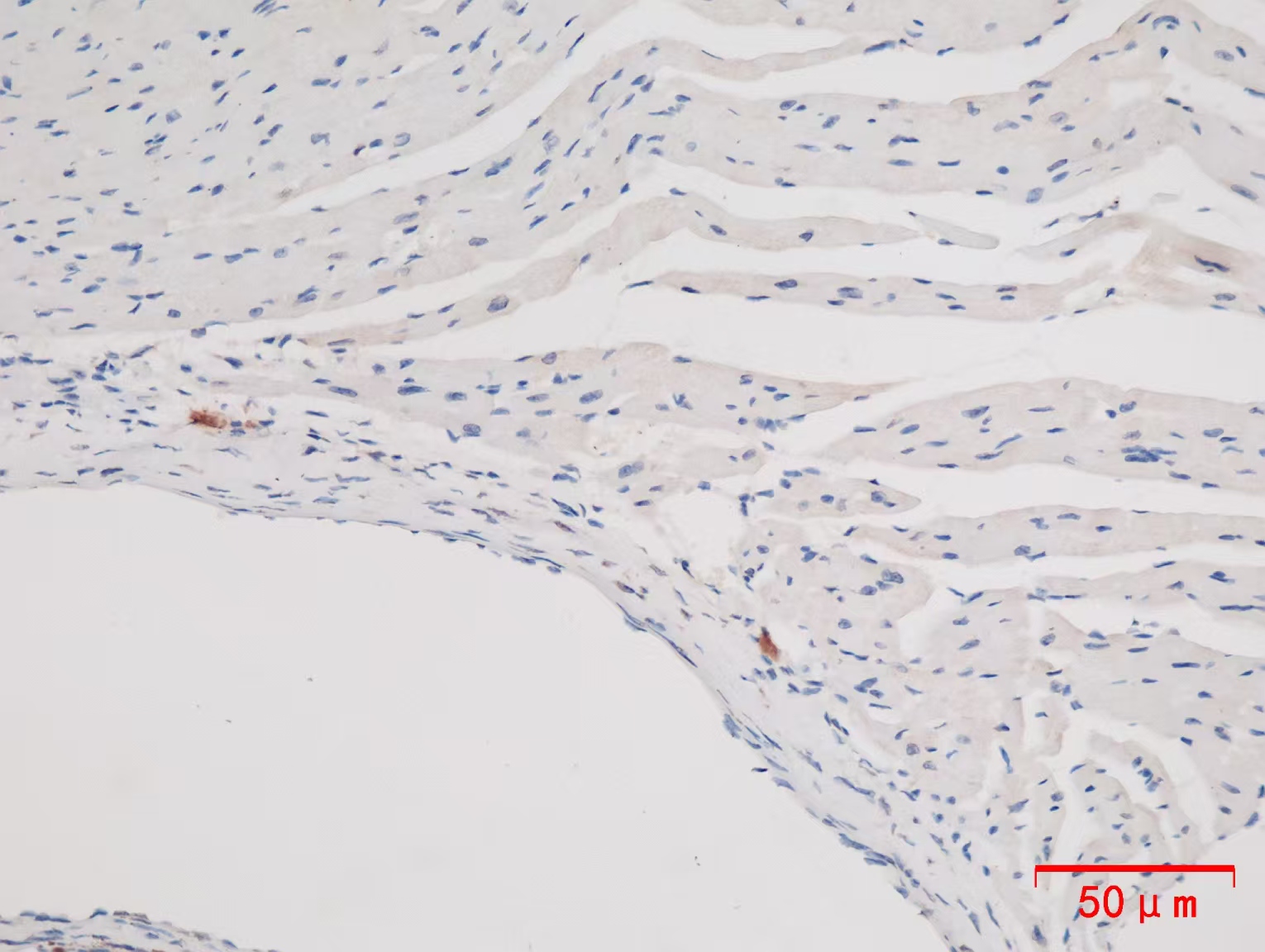

Supplement: Multimedia component 4 [file mmc4.zip › Original Figures/Figure 7. IHC and WB original images/Figure 7F. IHC/Figure 7F. Control 2.jpg]

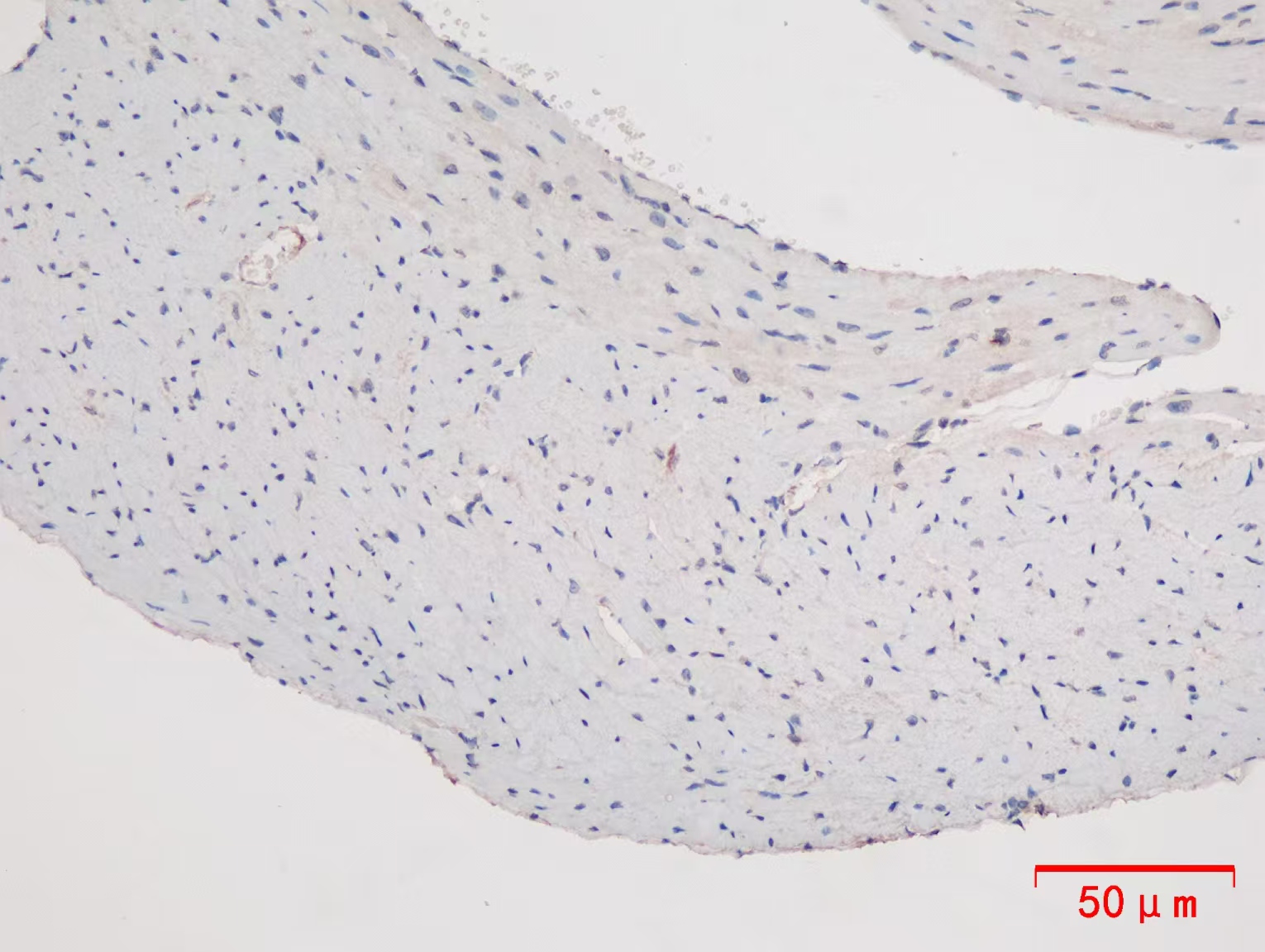

Supplement: Multimedia component 4 [file mmc4.zip › Original Figures/Figure 7. IHC and WB original images/Figure 7F. IHC/Figure 7F. Control 3.jpg]

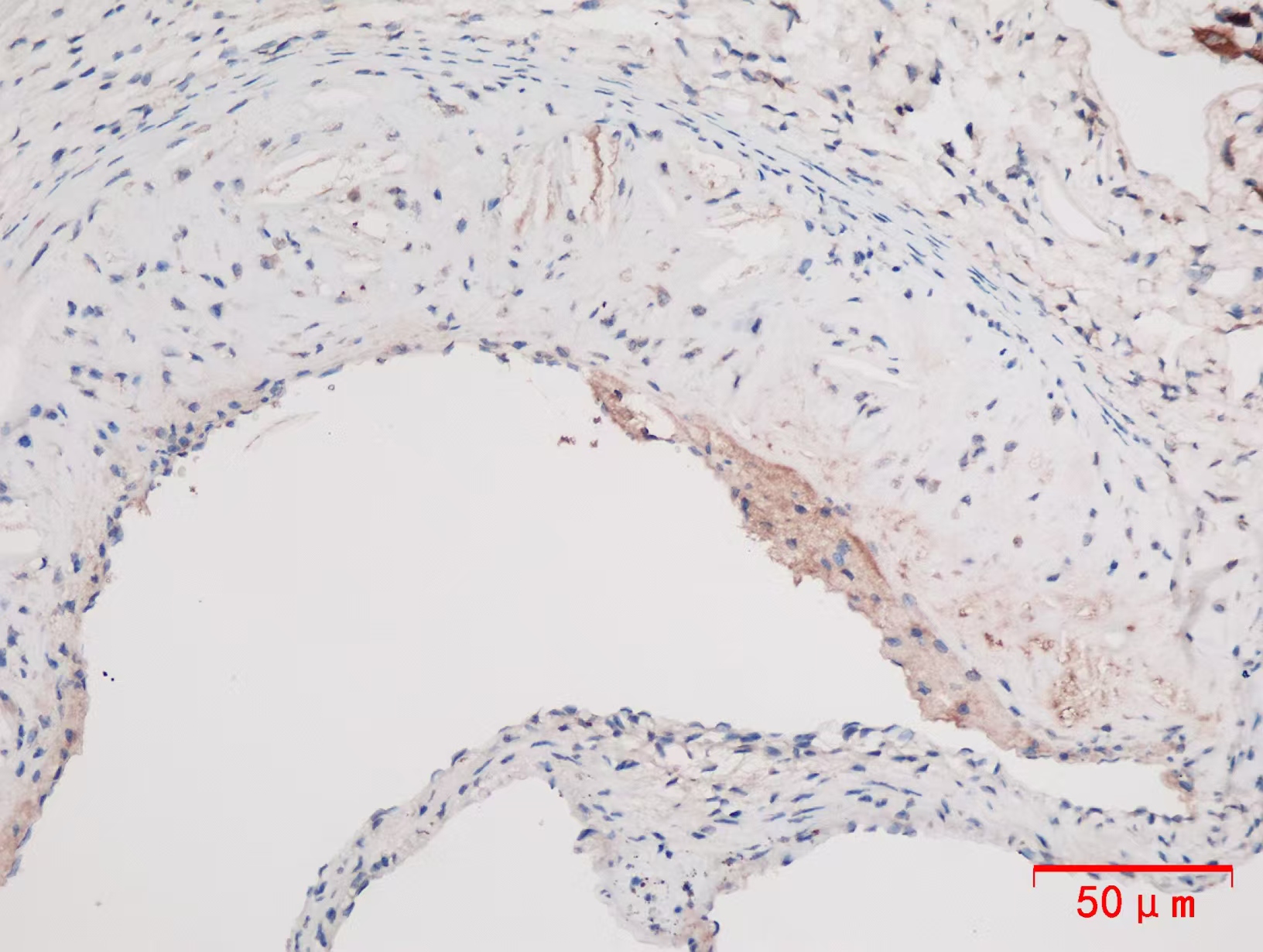

Supplement: Multimedia component 4 [file mmc4.zip › Original Figures/Figure 7. IHC and WB original images/Figure 7F. IHC/Figure 7F. HFD 1.jpg]

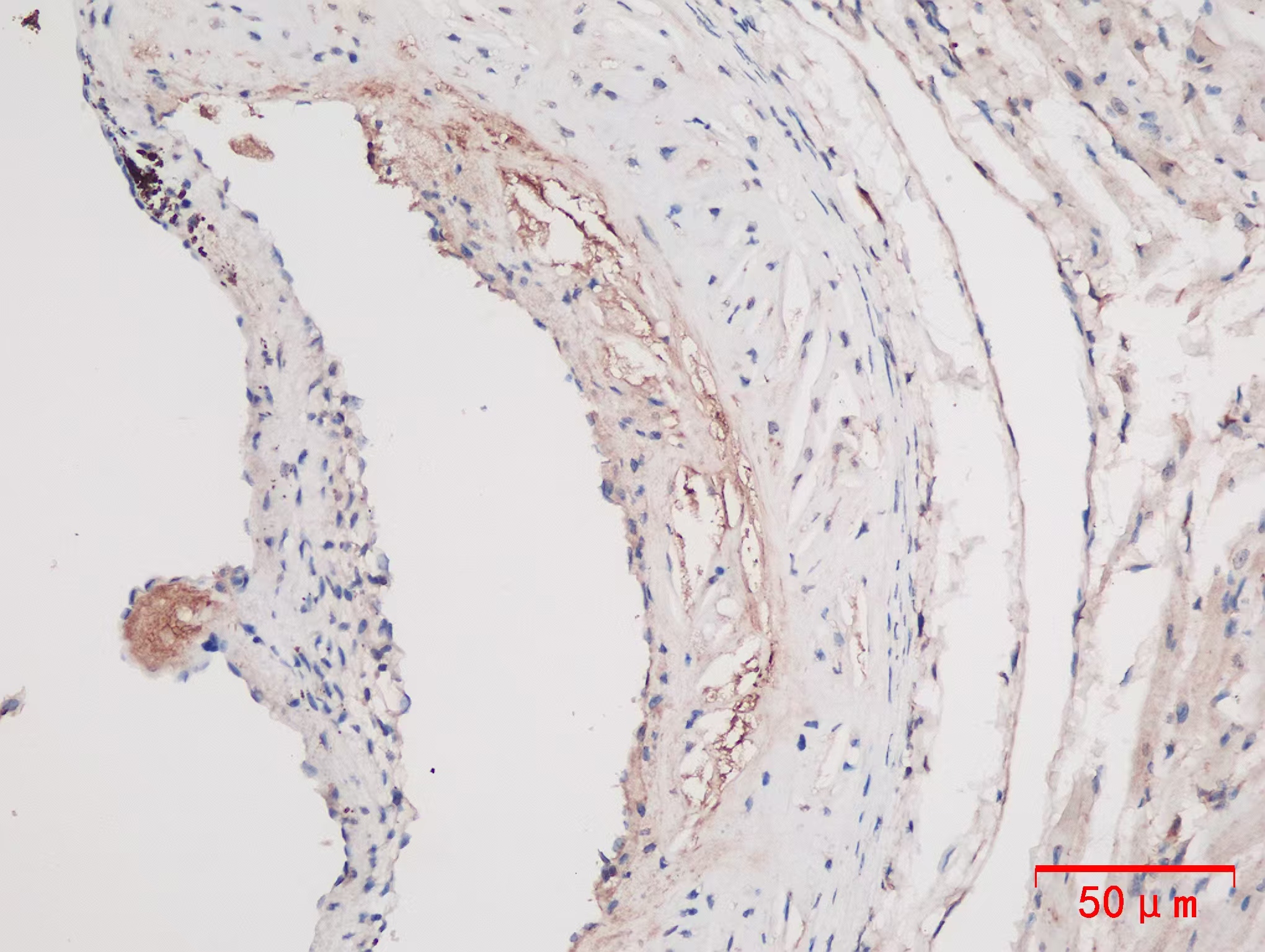

Supplement: Multimedia component 4 [file mmc4.zip › Original Figures/Figure 7. IHC and WB original images/Figure 7F. IHC/Figure 7F. HFD 2.jpg]

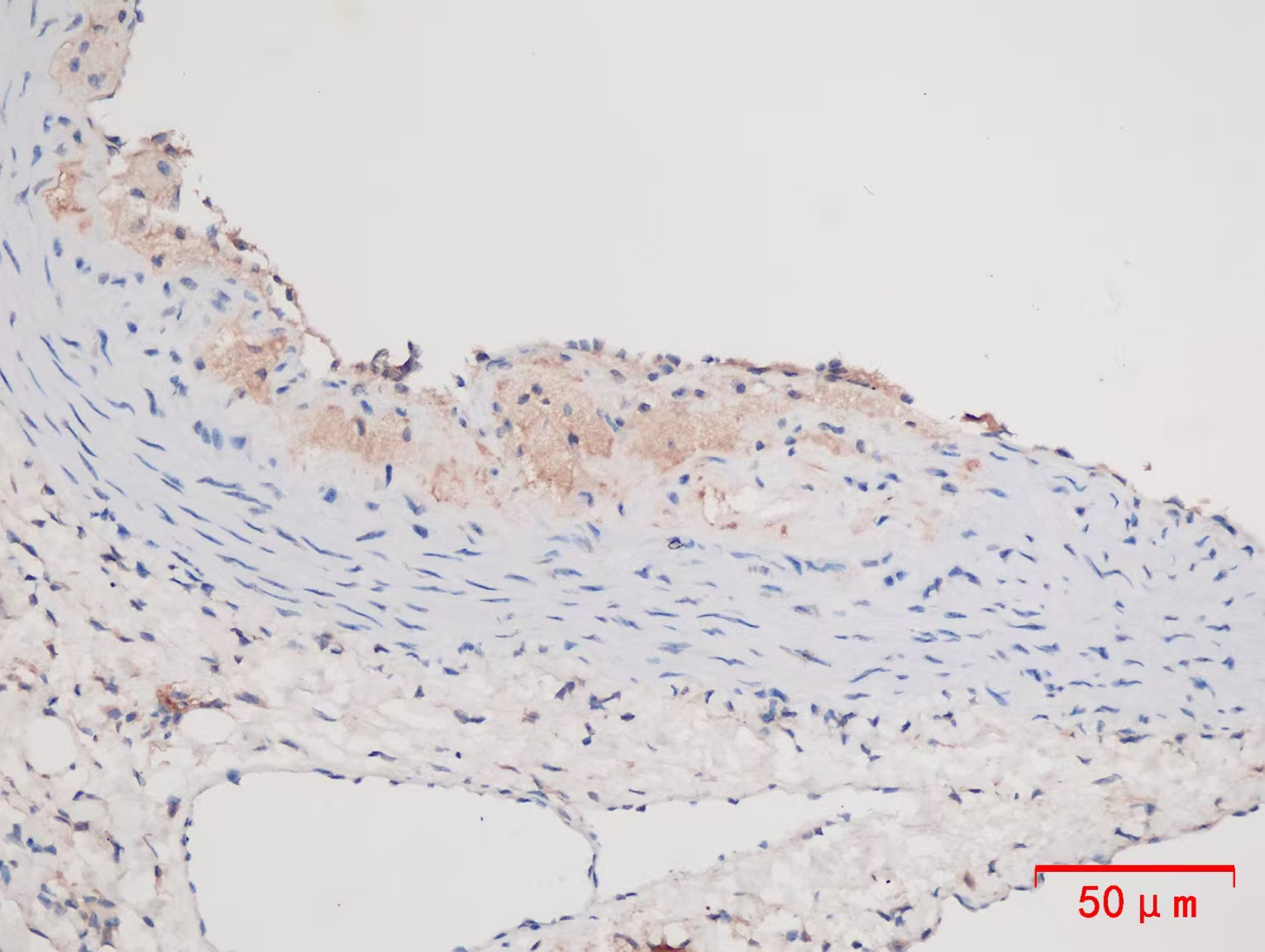

Supplement: Multimedia component 4 [file mmc4.zip › Original Figures/Figure 7. IHC and WB original images/Figure 7F. IHC/Figure 7F. HFD 3.jpg]
